# Data on the fungal species consumed by mammal species in Australia

**DOI:** 10.1016/j.dib.2017.03.053

**Published:** 2017-04-12

**Authors:** S.J. Nuske, K. Vernes, T.W. May, A.W. Claridge, B.C. Congdon, A. Krockenberger, S.E. Abell

**Affiliations:** a College of Science and Engineering, Centre for Tropical Environmental and Sustainability Science, James Cook University, Cairns, QLD 4878, Australia; b Ecosystem Management, University of New England, Armidale, NSW 2351, Australia; c Royal Botanic Gardens Victoria, Melbourne, VIC 3004, Australia; d Office of Environment and Heritage, NSW National Parks and Wildlife Service, Nature Conservation Section, P.O. Box 733, Queanbeyan, NSW 2620, Australia; e School of Physical, Environmental and Mathematical Sciences, The University of New South Wales at Canberra, Northcott Drive, Canberra, ACT 2600, Australia; f Division of Research and Innovation, James Cook University, Cairns, QLD 4878, Australia

**Keywords:** Mammal, Diet, Fungi, Spore dispersal, Mycophagy, Mycorrhizal

## Abstract

The data reported here support the manuscript Nuske et al. (2017) [1]. Searches were made for quantitative data on the occurrence of fungi within dietary studies of Australian mammal species. The original location reported in each study was used as the lowest grouping variable within the dataset. To standardise the data and compare dispersal events from populations of different mammal species that might overlap, data from locations were further pooled and averaged across sites if they occurred within 100 km of a random central point. Three locations in Australia contained data on several (>7) mycophagous mammals, all other locations had data on 1–3 mammal species. Within these three locations, the identity of the fungi species was compared between mammal species’ diets. A list of all fungi species found in Australian mammalian diets is also provide along with the original reference and fungal synonym names.

**Specifications Table**

**Table:** 

Subject area	*Ecology*
More specific subject area	*Mycophagy, consumption and dispersal of fungi by mammals*
Type of data	*Tables*
How data was acquired	*Literature search*
Data format	*Raw; summarized*
Experimental factors	*n/a*
Experimental features	*n/a*
Data source location	*Nuske et al.*[1]*; see reference list in*Table 4.
Data accessibility	*Summarized data are available with this article. All data are from published articles or from unpublished data outlined in Nuske et al.*[1].

**Value of the data**

• This data shows the differences in dietary fungal species of different mammals and hence their relative contribution to the dispersal of these species. Future studies can confirm these trends with targeted sampling of both mammalian fungal specialists and generalists.
• This data lists fungal species which only occur in endangered *Bettongia tropica* and *Potorous longipes* diets; further studies can target these species to confirm whether the absence of fungal specialists results in lower dispersal rates.
• Further studies can also target the listed fungal species in the data for the development of genetic markers or reference libraries to study gene flow and population genetic diversity in relation to different dispersal modes.

## 1 Data

Table 1, Table 2, Table 3 list fungal species recorded within mammal species diets within 100 km of the three locations; Table 1: North Queensland on Atherton Tablelands (17° 16′ 15.99′ S, 145° 38′ 2.00″ E); Table 2: Northern New South Wales on Gibraltar Range (29° 32′ 59.17″ S, 152° 16′ 0.50″ E); and Table 3: South Eastern NSW near Victorian border (37° 23′ 30.00″ S, 149° 49′ 19.99″ E). Fungal names are categorized into truffle-like or not and their mycorrhizal status using lists from [2], [3]. Table 4 lists fungal species recorded within Australian mammal species diets, per reference. Synonyms of fungal taxon names are also listed in Table 4, if appropriate.

## 2 Experimental design, materials and methods

Data were gathered from literature (references in Table 4). Methods for the development of the selection criteria for including the data is outlined in Nuske et al. [1]. Briefly, dietary studies of Australian mammals were searched from Web of Science and Google Scholar. Relevant theses and books were searched also. Because fungal spores are smaller than many other common dietary materials and spores are needed for identification of fungal taxa consumed, only studies that used conservative methods for collecting and examining dietary material were used in the dataset. Specially, these methods were the examination of fine fraction material (no material discarded), the use of 100× or greater magnification, and spores must have been identified by use of mycological literature and/or a mycological expert.

For each data point in each study, the location of the study was used as the lowest grouping variable. Data across studies were compared by pooling data together if they occurred within 100 km from a random central point. In comparisons, fungal names included both formally published and as yet unpublished names, identified at least to genus (value=1 in ‘Cf’ column of Table 4), but not taxa in the form ׳Unknown sp. 1׳ that were not identified to at least genus level (value=0) nor a few taxa (such as *Endoptychum* sp.) that could not be equated to modern genera.

## Figures and Tables

**Table 1:** Fungal species consumed by mammal species in North Queensland on the Atherton Tablelands. The first letter in parentheses after the fungal taxa name refers to whether the taxa are truffle-like (y), not truffle-like (n), or with taxa either truffle-like or not truffle-like (n/y). The second letter refers to whether the taxa are ectomycorrhizal (y), putatively ectomycorrhizal (y?), arbuscular mycorrhizal (AM), has other functional modes (n) or has unknown functional modes (?). These values are applied to the genera as a whole and/or species listed under a genus, unless otherwise specified. Fungal taxon names in bold are only in the diet of the fungal specialist, Northern Bettong (*Bettongia tropica*).

Fungal taxa	*Bettongia tropica*	*Aepyprymnus rufescens*	*Isoodon macrourus*	*Isoodon obesulus peninsulae*	*Perameles nasuta*	*Thylogale stigmatica*	*Uromys caudimaculatus*	Total
***Amylascus sp. (y, y?)***	**1**							**1**
*Aroramyces sp. (y, y?)*	1			1				2
***Aroramyces queenslandica***	**1**							**1**
*Austrogautieria sp. (y, y)*				1				1
*Austrogautieria amara*							1	1
***Austrogautieria chlorospora***	**1**							**1**
***Austrogautieria longispora nom. ined.***	**1**							**1**
***Beatonia sp. (y,?)***	**1**							**1**
***Castoreum sp. (y, y)***	**1**							**1**
*Chondrogaster sp. (y, y)*	1		1					2
*Cortinarius sp. (n/y, y)*	1			1				2
*Cribbea sp. (y, n)*						1		1
***Descomyces sp. (y, y)***	**1**							**1**
*Elaphomyces sp. (y, y)*	1	1	1					3
*Endogone sp. (y, y)*	1	1	1		1		1	5
***Gallacea sp. (y, y?)***	**1**							**1**
*Gautieria sp. (y, y)*	1			1		1		3
*Glomus sp. (y, AM)*	1			1			1	3
*Gummiglobus sp. (y, y)*	1	1	1		1		1	5
*Gymnohydnotrya sp. (y, y)*						1		1
***Hydnangium sp. (y, y)***	**1**							**1**
*Hydnoplicata sp. (y, y)*						1		1
*Hymenogaster sp. (y, y)*							1	1
*Hysterangium sp. (y, y)*	1	1	1	1	1	1	1	7
***Hysterogaster sp. (y, y?)***	**1**							**1**
*Hysterogaster sp. (y, y?)*				1				1
*Mesophellia sp. (y, y)*	1						1	2
*Mycoamaranthus auriorbis (y, y?)*	1	1						2
*Pogisperma sp. (y,?)*				1				1
*Pseudohysterangium sp. (y,?)*	1	1	1		1		1	5
*Rossbeevera sp. (y, y)*	1					1		2
***Royoungia boletoides (y, y?)***	**1**							**1**
*Scleroderma sp. (n/y, y)*	1		1				1	3
*Sclerogaster sp. (y,?)*	1	1	1					3
*Sphaerodes beatonii (n, n)*						1		1
*Sphaerosoma sp. (y, y?)*						1		1
*Stephanospora flava (y, n)*						1		1
***Timgrovea sp. (y, y?)***	**1**							**1**
*Zelleromyces sp. (y, y)*	1						1	2
Total	28	7	8	8	4	9	10	

**Table 2:** Fungal species consumed by mammal species in Northern New South Wales on the Gibraltar Range. Refer to Table 1 for annotation.

Fungal taxa	*Potorous tridactylus*		*Antechinus stuartii*	*Macropus parma*	*Melomys cervinipes*	*Perameles nasuta*	*Pseudomys novaehollandiae*	*Rattus fuscipes*	*Thylogale thetis*	*Trichosurus caninus*	*Wallabia bicolor*	Total
*Agaricus sp. (n, n)*			1	1	1				1		1	5
*Amylascus sp. (y, y?)*			1		1	1		1				4
*Arcangeliella sp. (y, y)*								1			1	2
*Aroramyces sp. (y, y?)*	1		1	1	1	1		1			1	7
*Austrogautieria sp. (y, y)*			1	1	1	1		1	1		1	7
*Boletellus sp. (n, y)*				1					1		1	3
*Chondrogaster sp. (y, y)*								1			1	2
*Cortinarius sp. (n/y, y)*	1		1	1	1	1		1	1	1	1	9
*Densospora sp. (y, y)*											1	1
*Descomyces sp. (y, y)*			1		1	1		1	1			5
*Descomyces stolatus*						1		1	1			3
*Dingleya sp. (y, y)*	1			1				1				3
*Elaphomyces sp. (y, y)*	1			1		1		1	1	1	1	7
*Endogone sp. (y, y)*							1	1				2
*Gautieria sp. (y, y)*				1		1					1	3
*Gautieria monospora*											1	1
*Glomus sp. (y, AM)*					1	1		1	1			4
*Hydnangium sp. (y, y)*					1	1		1				3
*Hydnoplicata sp. (y, y)*								1	1			2
*Hydnoplicata convoluta*			1			1		1			1	4
*Hysterangium sp. (y, y)*	1		1	1	1	1		1	1		1	8
*Hysterangium inflatum*								1				1
*Hysterogaster sp. (y, y?)*			1	1	1	1		1	1		1	7
*Labyrinthomyces sp. (y, y)*	1		1	1				1	1		1	6
*Leucogaster sp. (y, y)*					1	1		1				3
*Leucogaster meridionalis*	1										1	2
*Mesophellia sp. (y, y)*				1				1			1	3
*Octaviania sp. (y, y)*				1				1	1		1	4
*Pogisperma sp. (y,?)*								1			1	2
*Protubera sp. (y, y?)*						1		1				2
*Rossbeevera sp. (y, y)*			1	1	1	1		1	1		1	7
*Rossbeevera vittatispora*											1	1
*Scleroderma sp. (n/y, y)*			1	1	1	1		1	1	1	1	8
*Scleroderma tommayi*			1	1				1			1	4
*Sclerogaster sp. (y,?)*								1	1		1	3
*Sphaerosoma sp. (y, y?)*								1			1	2
*Stephanospora sp. (y, n)*								1				1
*Timgrovea sp. (y, y?)*								1				1
Total	7		13	16	13	17	1	31	16	3	25	

**Table 3:** Fungal species consumed by mammal species in South Eastern NSW near the Victorian border. Refer to Table 1 for annotation. Fungal species in bold are only in the diet of fungal specialists, *Potorous* spp.

Fungal taxa	*Potorous longipes*	*Potorous tridactylus*	*Isoodon obesulus*	*Perameles nasuta*	*Pseudomys fumeus*	*Rattus fuscipes*	*Trichosurus caninus*	*Wallabia bicolor*	Total
***Acaulospora sp. (?, AM)***	**1**								**1**
***Aleuria aurantia (n, n)***	**1**								**1**
***Aleurina calospora (n, y)***	**1**								**1**
***Amanita sp. (n, y)***	**1**								**1**
***Amanita grandispora***		**1**							**1**
***Amarrendia lignicolor (y, y?)***	**1**								**1**
***Amylascus tasmanicus (y, y?)***	**1**	**1**							**2**
***Andebbia pachythrix (y, y?)***		**1**							**1**
***Aroramyces gelatinosporus (y, y?)***		**1**							**1**
***Austrogautieria costata (y, y?)***	**1**	**1**							**2**
*Castoreum sp. (y, y?)*		1		1					2
***Castoreum tasmanicum***		**1**							**1**
*Cortinarius sp. (n/y, y)*	1	1					1		3
*Cortinarius atratus (y, y)*		1		1					2
***Cortinarius leucocephalus (y, y)***		**1**							**1**
***Cortinarius levisporus (y, y)***		**1**							**1**
***Cortinarius oblongisporus (y, y)***	**1**	**1**							**2**
***Cortinarius oleosus (y, y)***		**1**							**1**
***Cortinarius piriformis (y, y)***	**1**								**1**
*Cortinarius scabrosus (y, y)*		1		1					2
***Cortinarius subviolaceus (y, y)***	**1**								**1**
***Cystangium sp. (y, y?)***	**1**								**1**
***Cystangium phymatodisporum***	**1**								**1**
***Cystangium rodwayi***	**1**								**1**
*Descomyces albellus (y, y)*		1		1					2
*Descomyces albus (y, y)*	1	1		1					3
***Dingleya tessellata (y, y)***	**1**	**1**							**2**
*Endogone sp. (y, y)*	1	1		1	1		1		5
*Entoloma gasteromycetoides (n, y)*		1		1					2
*Gautieria sp. (y, y)*		1		1			1		3
***Gautieria albida***	**1**	**1**							**2**
*Gautieria monospora*		1		1					2
***Geoglossum sp. sens. Lat. (n, n)***	**1**								**1**
***Gymnohydnotrya echinulata (y, y)***		**1**							**1**
*Gymnomyces sp. (y, y)*								1	1
***Gymnomyces pallidus***	**1**								**1**
***Gymnomyces redolens***	**1**	**1**							**2**
***Gymnomyces seminudus***	**1**								**1**
*Hydnangium sp. (y, y)*				1					1
***Hydnangium archeri***	**1**	**1**							**2**
*Hydnangium carneum*							1	1	2
***Hydnoplicata convoluta (y, y)***		**1**							**1**
*Hymenangium album (y,?)*						1			1
*Hymenogaster sp. (y, y)*	1			1	1		1		4
***Hymenogaster aureus***	**1**	**1**							**2**
*Hymenogaster inflatum*				1					1
*Hymenogaster nanus*		1		1					2
*Hysterangium sp. (y, y)*	1	1			1			1	4
***Hysterangium affine***	**1**								**1**
***Hysterangium aggregatum***	**1**	**1**							**2**
*Hysterangium inflatum*	1	1				1			3
***Hysterangium salmonaceum***		**1**							**1**
***Hysterogaster fusisporus (y, y?)***		**1**							**1**
*Jafneadelphus sp. (n, y?)*		1		1			1		3
*Labyrinthomyces sp. (y, y)*							1		1
*Labyrinthomyces varius*	1	1		1					3
***Lamprospora sp. (n, n)***	**1**								**1**
***Lamprospora crechqueraultii***	**1**								**1**
***Leucogaster sp. (y, y)***	**1**								**1**
***Leucogaster meridionalis***		**1**							**1**
*Mesophellia sp. (y, y)*	1	1	1	1	1		1		6
*Octaviania sp. (y, y)*					1		1		2
*Octaviania tasmanica*	1	1		1					3
*Podohydnangium sp. (y, y?)*	1						1		2
***Richoniella sp. (y, y?)***	**1**								**1**
*Rossbeevera sp. (y, y)*	1	1		1				1	4
***Rossbeevera mucosa***	**1**								**1**
***Rossbeevera pachydermis***		**1**							**1**
*Rossbeevera vittatispora*	1	1		1			1		4
*Scleroderma sp. (n/y, y)*	1							1	2
***Scleroderma paradoxum (y, y)***	**1**	**1**							**2**
***Sphaerodes beatonii (n, n)***	**1**								**1**
*Stephanospora flava (y, n)*	1	1					1		3
***Timgrovea macrospora (y, y?)***		**1**							**1**
***Timgrovea reticulata (y, y?)***		**1**							**1**
*Zelleromyces sp. (y, y)*	1	1		1			1	1	5
***Zelleromyces australiensis***		**1**							**1**
*Zelleromyces daucinus*		1		1					2
***Zelleromyces malaiensis***		**1**							**1**
***Zelleromyces striatus***	**1**	**1**							**2**
Total	46	50	1	21	5	2	13	6	
