# Supplementary material for: Data on the fungal species consumed by mammal species in Australia
Source: Data Brief. 2017 Apr 12;12:251–60. doi: 10.1016/j.dib.2017.03.053 (PMC5402625; doi:10.1016/j.dib.2017.03.053)
Supplement: Supplementary file 1 — Supplementary material [file mmc1.pdf]

Conflict of Interest Form

I wish to confirm that there are no known conflicts of interest associated with this publication (Manuscript number: DIB-D-17-00180) and there has been no significant financial support for this work that could have influenced its outcome.

I confirm that I have given due consideration to the protection of intellectual property associated with this work and that there are no impediments to publication, including the timing of publication, with respect to intellectual property. In so doing we confirm that we have followed the regulations of our institutions concerning intellectual property.

Sincerely,

Susan Nuske

Susan Nuske

Digitally signed by Susan Nuske
Date: 2017.03.20 16:25:15 +01'00'
