# Supplementary material for: Data on the fungal species consumed by mammal species in Australia
Source: Data Brief. 2017 Apr 12;12:251–60. doi: 10.1016/j.dib.2017.03.053 (PMC5402625; doi:10.1016/j.dib.2017.03.053)
Supplement: Supplementary file 2 — Supplementary material [file mmc2.docx]

Table 4: List of all fungal species found in Australian mammalian diets, with the original reference and fungal synonym names. Fungal phylums are A: Ascomycota, B: Basidiomycota, G: Glomeromycota, u: Unknown, and Z: Zygomycota. Cf: Binary notation for the fungal names that are used in comparisons between different mammal species diets (1) or not (0). Ref: see full references in Nuske et al. (2017).

| Mammal Species | Phylum | Fungal Order | Fungal Family | Fungal Genus | Fungal Species (original) | Synonym | Cf | Ref |
| --- | --- | --- | --- | --- | --- | --- | --- | --- |
| *Aepyprymnus rufescens* | B | Agaricales | Agaricaceae | *Agaricus* | *Agaricus sp.1 Vernes, K* | *Agaricus* | 1 | Vernes 2010 |
| *Aepyprymnus rufescens* | B | Agaricales | Agaricaceae | *Agaricus* | *Agaricus sp.2 Vernes, K* | *Agaricus* | 1 | Vernes 2010 |
| *Aepyprymnus rufescens* | B | Agaricales | Cortinariaceae | *Aroramyces* | *Aroramyces sp.1 Vernes, K* | *Aroramyces* | 1 | Vernes 2010 |
| *Aepyprymnus rufescens* | B | Agaricales | Cortinariaceae | *Aroramyces* | *Aroramyces sp.2 Vernes, K* | *Aroramyces* | 1 | Vernes 2010 |
| *Aepyprymnus rufescens* | B | Hysterangiales | Gallaceaceae | *Austrogautieria* | *Austrogautieria* | *Austrogautieria* | 1 | Vernes 2010 |
| *Aepyprymnus rufescens* | B | Boletales | Boletaceae | *Boletellus* | *Boletellus* | *Boletellus* | 1 | Vernes 2010 |
| *Aepyprymnus rufescens* | B | Boletales | Boletaceae | *Chamonixia* | *Chamonixia* | *Rossbeevera* | 1 | Vernes 2010 |
| *Aepyprymnus rufescens* | B | Agaricales | Cortinariaceae | *Descomyces* | *Descomyces sp.2 Vernes, K* | *Descomyces* | 1 | Vernes 2010 |
| *Aepyprymnus rufescens* | A | Pezizales | Tuberaceae | *Dingleya* | *Dingleya* | *Dingleya* | 1 | Vernes 2010 |
| *Aepyprymnus rufescens* | A | Elaphomycetales | Elaphomycetaceae | *Elaphomyces* | *Elaphomyces* | *Elaphomyces* | 1 | Reddell et al. 1997 |
| *Aepyprymnus rufescens* | A | Elaphomycetales | Elaphomycetaceae | *Elaphomyces* | *Elaphomyces* | *Elaphomyces* | 1 | Vernes 2010 |
| *Aepyprymnus rufescens* | Z | Endogonales | Endogonaceae | *Endogone* | *Endogone* | *Endogone* | 1 | Reddell et al. 1997 |
| *Aepyprymnus rufescens* | B | Hysterangiales | Mesophelliaceae | *Gummiglobus* | *Gummiglobus* | *Gummiglobus* | 1 | Reddell et al. 1997 |
| *Aepyprymnus rufescens* | A | Pezizales | Pezizaceae | *Hydnoplicata* | *Hydnoplicata convoluta* | *Hydnoplicata convoluta* | 1 | Vernes 2010 |
| *Aepyprymnus rufescens* | B | Hysterangiales | Hysterangiaceae | *Hysterangium* | *Hysterangium* | *Hysterangium* | 1 | Reddell et al. 1997 |
| *Aepyprymnus rufescens* | B | Hysterangiales | Hysterangiaceae | *Hysterangium* | *Hysterangium* | *Hysterangium* | 1 | Vernes 2010 |
| *Aepyprymnus rufescens* | B | Hysterangiales | Hysterangiaceae | *Hysterangium* | *Hysterangium inflatum* | *Hysterangium inflatum* | 1 | Vernes 2010 |
| *Aepyprymnus rufescens* | B | Russulales | Albatrellaceae | *Leucogaster* | *Leucogaster meridionalis* | *Leucogaster meridionalis* | 1 | Vernes 2010 |
| *Aepyprymnus rufescens* | B | Boletales | Boletaceae | *Mycoamaranthus* | *Mycoamaranthus auriorbis* | *Mycoamaranthus auriorbis* | 1 | Reddell et al. 1997 |
| *Aepyprymnus rufescens* | B | Boletales | Octavianiaceae | *Octaviania* | *Octaviania* | *Octaviania* | 1 | Vernes 2010 |
| *Aepyprymnus rufescens* | B | Agaricales | Cortinariaceae | *Protoglossum* | *Protoglossum* | *Cortinarius* | 1 | Vernes 2010 |
| *Aepyprymnus rufescens* | B | unknown | unknown | *Pseudohysterangium* | *Pseudohysterangium* | *Pseudohysterangium* | 1 | Reddell et al. 1997 |
| *Aepyprymnus rufescens* | B | Agaricales | Cortinariaceae | *Quadrispora* | *Quadrispora oblongispora* | *Cortinarius oblongisporus* | 1 | Vernes 2010 |
| *Aepyprymnus rufescens* | B | Boletales | Sclerodermataceae | *Scleroderma* | *Scleroderma sp.2 Vernes, K* | *Scleroderma* | 1 | Vernes 2010 |
| *Aepyprymnus rufescens* | B | Boletales | Sclerodermataceae | *Scleroderma* | *Scleroderma sp.3 Vernes, K* | *Scleroderma* | 1 | Vernes 2010 |
| *Aepyprymnus rufescens* | B | Boletales | Sclerodermataceae | *Scleroderma* | *Scleroderma tommayi* | *Scleroderma tommayi* | 1 | Vernes 2010 |
| *Aepyprymnus rufescens* | B | Geastrales | Geastraceae | *Sclerogaster* | *Sclerogaster* | *Sclerogaster* | 1 | Reddell et al. 1997 |
| *Aepyprymnus rufescens* | B | Agaricales | Cortinariaceae | *Thaxterogaster* | *Thaxterogaster* | *Cortinarius* | 1 | Vernes 2010 |
| *Aepyprymnus rufescens* | B | Agaricales | unknown | *Timgrovea* | *Timgrovea* | *Timgrovea* | 1 | Vernes 2010 |
| *Aepyprymnus rufescens* | B | unknown | unknown | *Unknown* | *Unknown* |  | 0 | Reddell et al. 1997 |
| *Aepyprymnus rufescens* | B | Agaricales | Cortinariaceae | *Unknown* | *Unknown* |  | 0 | Reddell et al. 1997 |
| *Aepyprymnus rufescens* | B | Hysterangiales | Mesophelliaceae | *Unknown* | *Unknown* |  | 0 | Reddell et al. 1997 |
| *Aepyprymnus rufescens* | B | Agaricales | Russulaceae | *Unknown* | *Unknown* |  | 0 | Reddell et al. 1997 |
| *Aepyprymnus rufescens* | B | Boletales | unknown | *Unknown* | *Unknown sp.1 Vernes, K* |  | 0 | Vernes 2010 |
| *Aepyprymnus rufescens* | u | unknown | unknown | *Unknown* | *Unknown sp.1 Vernes, K* |  | 0 | Vernes 2010 |
| *Aepyprymnus rufescens* | B | Russulales | unknown | *Unknown* | *Unknown sp.2 Vernes, K* |  | 0 | Vernes 2010 |
| *Aepyprymnus rufescens* | u | unknown | unknown | *Unknown* | *Unknown sp.2 Vernes, K* |  | 0 | Vernes 2010 |
| *Aepyprymnus rufescens* | u | unknown | unknown | *Unknown* | *Unknown sp.5 Vernes, K* |  | 0 | Vernes 2010 |
| *Aepyprymnus rufescens* | B | Russulales | unknown | *Unknown* | *Unknown sp.6 Vernes, K* |  | 0 | Vernes 2010 |
| *Aepyprymnus rufescens* | u | unknown | unknown | *Unknown* | *Unknown sp.6 Vernes, K* |  | 0 | Vernes 2010 |
| *Aepyprymnus rufescens* | u | unknown | unknown | *Unknown* | *Unknown sp.7 Vernes, K* |  | 0 | Vernes 2010 |
| *Antechinus stuartii* | B | Agaricales | Agaricaceae | *Agaricus* | *Agaricus* | *Agaricus* | 1 | Vernes et al. 2015 |
| *Antechinus stuartii* | A | Pezizales | Pezizaceae | *Amylascus* | *Amylascus* | *Amylascus* | 1 | Vernes et al. 2015 |
| *Antechinus stuartii* | B | Agaricales | Cortinariaceae | *Aroramyces* | *Aroramyces sp.1 Vernes, K* | *Aroramyces* | 1 | Vernes et al. 2015 |
| *Antechinus stuartii* | B | Hysterangiales | Gallaceaceae | *Austrogautieria* | *Austrogautieria* | *Austrogautieria* | 1 | Vernes et al. 2015 |
| *Antechinus stuartii* | B | Boletales | Boletaceae | *Chamonixia* | *Chamonixia* | *Rossbeevera* | 1 | Vernes et al. 2015 |
| *Antechinus stuartii* | B | Agaricales | Cortinariaceae | *Descomyces* | *Descomyces sp.2 Vernes, K* | *Descomyces* | 1 | Vernes et al. 2015 |
| *Antechinus stuartii* | B | Agaricales | Cortinariaceae | *Descomyces* | *Descomyces sp.3 Vernes, K* | *Descomyces* | 1 | Vernes et al. 2015 |
| *Antechinus stuartii* | B | Agaricales | Agaricaceae | *Endoptychum* | *Endoptychum* |  | 0 | Vernes et al. 2015 |
| *Antechinus stuartii* | A | Pezizales | Pezizaceae | *Hydnoplicata* | *Hydnoplicata convoluta* | *Hydnoplicata convoluta* | 1 | Vernes et al. 2015 |
| *Antechinus stuartii* | B | Hysterangiales | Hysterangiaceae | *Hysterangium* | *Hysterangium* | *Hysterangium* | 1 | Vernes et al. 2015 |
| *Antechinus stuartii* | B | Agaricales | Strophariaceae | *Hysterogaster* | *Hysterogaster sp.3 Vernes, K* | *Hysterogaster* | 1 | Vernes et al. 2015 |
| *Antechinus stuartii* | A | Pezizales | Tuberaceae | *Labyrinthomyces* | *Labyrinthomyces sp.2 Vernes, K* | *Labyrinthomyces* | 1 | Vernes et al. 2015 |
| *Antechinus stuartii* | B | Boletales | Sclerodermataceae | *Scleroderma* | *Scleroderma* | *Scleroderma* | 1 | Vernes et al. 2015 |
| *Antechinus stuartii* | B | Boletales | Sclerodermataceae | *Scleroderma* | *Scleroderma tommayi* | *Scleroderma tommayi* | 1 | Vernes et al. 2015 |
| *Antechinus stuartii* | B | Agaricales | Cortinariaceae | *Thaxterogaster* | *Thaxterogaster sp.1 Vernes, K* | *Cortinarius* | 1 | Vernes et al. 2015 |
| *Antechinus stuartii* | u | unknown | unknown | *Unknown* | *Unknown* |  | 0 | Vernes et al. 2015 |
| *Antechinus stuartii* | A | unknown | unknown | *Unknown* | *Unknown sp.1 Vernes, K* |  | 0 | Vernes et al. 2015 |
| *Antechinus stuartii* | B | Russulales | unknown | *Unknown* | *Unknown sp.1 Vernes, K* |  | 0 | Vernes et al. 2015 |
| *Antechinus stuartii* | u | unknown | unknown | *Unknown* | *Unknown sp.13 Vernes, K* |  | 0 | Vernes et al. 2015 |
| *Antechinus stuartii* | A | unknown | unknown | *Unknown* | *Unknown sp.2 Vernes, K* |  | 0 | Vernes et al. 2015 |
| *Antechinus stuartii* | B | Russulales | unknown | *Unknown* | *Unknown sp.2 Vernes, K* |  | 0 | Vernes et al. 2015 |
| *Antechinus stuartii* | B | Russulales | unknown | *Unknown* | *Unknown sp.3 Vernes, K* |  | 0 | Vernes et al. 2015 |
| *Antechinus stuartii* | u | unknown | unknown | *Unknown* | *Unknown sp.3 Vernes, K* |  | 0 | Vernes et al. 2015 |
| *Bettongia gaimardi* | A | Pezizales | Pyronemataceae | *Aleuria* | *Aleuria aurantia* | *Aleuria aurantia* | 1 | Taylor 1992 |
| *Bettongia gaimardi* | A | Pezizales | Pyronemataceae | *Aleuria* | *Aleuria aurantia* | *Aleuria aurantia* | 1 | Taylor 1988 |
| *Bettongia gaimardi* | B | Boletales | Melanogastraceae | *Alpova* | *Alpova* | *Amanita* | 1 | Johnson 1994b |
| *Bettongia gaimardi* | B | Boletales | Melanogastraceae | *Alpova* | *Alpova* | *Amanita* | 1 | Taylor 1992 |
| *Bettongia gaimardi* | B | Boletales | Melanogastraceae | *Alpova* | *Alpova* | *Amanita* | 1 | C. Johnson |
| *Bettongia gaimardi* | B | Boletales | Melanogastraceae | *Alpova* | *Alpova clelandii* |  | 0 | Taylor 1988 |
| *Bettongia gaimardi* | B | Boletales | Melanogastraceae | *Alpova* | *Alpova grandisporus* | *Amanita grandispora* | 1 | Johnson 1994b |
| *Bettongia gaimardi* | B | Boletales | Melanogastraceae | *Alpova* | *Alpova lignicolor* | *Amarrendia lignicolor* | 1 | Taylor 1988 |
| *Bettongia gaimardi* | A | Pezizales | Pezizaceae | *Amylascus* | *Amylascus tasmanicus* | *Amylascus tasmanicus* | 1 | Taylor 1992 |
| *Bettongia gaimardi* | B | Hysterangiales | Gallaceaceae | *Austrogautieria* | *Austrogautieria costata* | *Austrogautieria costata* | 1 | C. Johnson |
| *Bettongia gaimardi* | B | Hysterangiales | Mesophelliaceae | *Castoreum* | *Castoreum* | *Castoreum* | 1 | Johnson 1994b |
| *Bettongia gaimardi* | B | Boletales | Boletaceae | *Chamonixia* | *Chamonixia* | *Rossbeevera* | 1 | Johnson 1994b |
| *Bettongia gaimardi* | B | Boletales | Boletaceae | *Chamonixia* | *Chamonixia* | *Rossbeevera* | 1 | Taylor 1992 |
| *Bettongia gaimardi* | B | Boletales | Boletaceae | *Chamonixia* | *Chamonixia mucosa* | *Rossbeevera mucosa* | 1 | Taylor 1988 |
| *Bettongia gaimardi* | B | Boletales | Boletaceae | *Chamonixia* | *Chamonixia vittatispora* | *Rossbeevera vittatispora* | 1 | Taylor 1988 |
| *Bettongia gaimardi* | B | Russulales | Russulaceae | *Cystangium* | *Cystangium phymatodisporum* | *Cystangium phymatodisporum* | 1 | Johnson 1994b |
| *Bettongia gaimardi* | B | Russulales | Russulaceae | *Cystangium* | *Cystangium phymatodisporum* | *Cystangium phymatodisporum* | 1 | Taylor 1988 |
| *Bettongia gaimardi* | B | Russulales | Russulaceae | *Cystangium* | *Cystangium rodwayi* | *Cystangium rodwayi* | 1 | C. Johnson |
| *Bettongia gaimardi* | A | Elaphomycetales | Elaphomycetaceae | *Elaphomyces* | *Elaphomyces* | *Elaphomyces* | 1 | C. Johnson |
| *Bettongia gaimardi* | Z | Endogonales | Endogonaceae | *Endogone* | *Endogone* | *Endogone* | 1 | Taylor 1992 |
| *Bettongia gaimardi* | Z | Endogonales | Endogonaceae | *Endogone* | *Endogone* | *Endogone* | 1 | Taylor 1988 |
| *Bettongia gaimardi* | B | Gomphales | Gomphaceae | *Gautieria* | *Gautieria* | *Gautieria* | 1 | Johnson 1994b |
| *Bettongia gaimardi* | B | Gomphales | Gomphaceae | *Gautieria* | *Gautieria albida* | *Gautieria albida* | 1 | Taylor 1988 |
| *Bettongia gaimardi* | B | Gomphales | Gomphaceae | *Gautieria* | *Gautieria costata* | *Austrogautieria costata* | 1 | Taylor 1988 |
| *Bettongia gaimardi* | B | Gomphales | Gomphaceae | *Gautieria* | *Gautieria macrospora* | *Austrogautieria macrospora* | 1 | Johnson 1994b |
| *Bettongia gaimardi* | B | Gomphales | Gomphaceae | *Gautieria* | *Gautieria monospora* | *Gautieria monospora* | 1 | Johnson 1994b |
| *Bettongia gaimardi* | B | Gomphales | Gomphaceae | *Gautieria* | *Gautieria* | *Gautieria* | 1 | Taylor 1992 |
| *Bettongia gaimardi* | B | Geastrales | Geastraceae | *Geastrum* | *Geastrum* | *Geastrum* | 1 | Taylor 1992 |
| *Bettongia gaimardi* | B | Russulales | Russulaceae | *Gymnomyces* | *Gymnomyces* | *Gymnomyces* | 1 | C. Johnson |
| *Bettongia gaimardi* | B | Russulales | Russulaceae | *Gymnomyces* | *Gymnomyces pallidus* | *Gymnomyces pallidus* | 1 | Taylor 1988 |
| *Bettongia gaimardi* | B | Agaricales | Hydnangiaceae | *Hydnangium* | *Hydnangium* | *Hydnangium* | 1 | Johnson 1994b |
| *Bettongia gaimardi* | B | Agaricales | Hydnangiaceae | *Hydnangium* | *Hydnangium archeri* | *Hydnangium archeri* | 1 | Taylor 1992 |
| *Bettongia gaimardi* | B | Agaricales | Hydnangiaceae | *Hydnangium* | *Hydnangium archeri* | *Hydnangium archeri* | 1 | Taylor 1988 |
| *Bettongia gaimardi* | B | Agaricales | Strophariaceae | *Hymenogaster* | *Hymenogaster* | *Hymenogaster* | 1 | Taylor 1992 |
| *Bettongia gaimardi* | B | Agaricales | Strophariaceae | *Hymenogaster* | *Hymenogaster* | *Hymenogaster* | 1 | Taylor 1988 |
| *Bettongia gaimardi* | B | Agaricales | Strophariaceae | *Hymenogaster* | *Hymenogaster atratus* | *Cortinarius atratus* | 1 | Johnson 1994b |
| *Bettongia gaimardi* | B | Agaricales | Strophariaceae | *Hymenogaster* | *Hymenogaster atratus* | *Cortinarius atratus* | 1 | Taylor 1988 |
| *Bettongia gaimardi* | B | Agaricales | Strophariaceae | *Hymenogaster* | *Hymenogaster oblongisporus* | *Cortinarius oblongisporus* | 1 | Johnson 1994b |
| *Bettongia gaimardi* | B | Agaricales | Strophariaceae | *Hymenogaster* | *Hymenogaster oblongisporus* | *Cortinarius oblongisporus* | 1 | Taylor 1988 |
| *Bettongia gaimardi* | B | Agaricales | Strophariaceae | *Hymenogaster* | *Hymenogaster violaceus* | *Cortinarius subviolaceus* | 1 | Johnson 1994b |
| *Bettongia gaimardi* | B | Agaricales | Strophariaceae | *Hymenogaster* | *Hymenogaster violaceus* | *Cortinarius subviolaceus* | 1 | Taylor 1988 |
| *Bettongia gaimardi* | B | Agaricales | Strophariaceae | *Hymenogaster* | *Hymenogaster zeylanicus* | *Descomyces albellus* | 1 | Johnson 1994b |
| *Bettongia gaimardi* | B | Agaricales | Strophariaceae | *Hymenogaster* | *Hymenogaster zeylanicus* | *Descomyces albellus* | 1 | Taylor 1988 |
| *Bettongia gaimardi* | B | Hysterangiales | Hysterangiaceae | *Hysterangium* | *Hysterangium* | *Hysterangium* | 1 | Taylor 1992 |
| *Bettongia gaimardi* | B | Hysterangiales | Hysterangiaceae | *Hysterangium* | *Hysterangium* | *Hysterangium* | 1 | Taylor 1988 |
| *Bettongia gaimardi* | B | Hysterangiales | Hysterangiaceae | *Hysterangium* | *Hysterangium affine* | *Hysterangium affine* | 1 | Taylor 1988 |
| *Bettongia gaimardi* | B | Hysterangiales | Hysterangiaceae | *Hysterangium* | *Hysterangium affine* | *Hysterangium affine* | 1 | C. Johnson |
| *Bettongia gaimardi* | B | Hysterangiales | Hysterangiaceae | *Hysterangium* | *Hysterangium aggregatum* | *Hysterangium aggregatum* | 1 | C. Johnson |
| *Bettongia gaimardi* | B | Hysterangiales | Hysterangiaceae | *Hysterangium* | *Hysterangium gelatinosporum* | *Aroramyces gelatinosporus* | 1 | Johnson 1994b |
| *Bettongia gaimardi* | B | Hysterangiales | Hysterangiaceae | *Hysterangium* | *Hysterangium inflatum* | *Hysterangium inflatum* | 1 | Taylor 1992 |
| *Bettongia gaimardi* | B | Hysterangiales | Hysterangiaceae | *Hysterangium* | *Hysterangium inflatum* | *Hysterangium inflatum* | 1 | Taylor 1988 |
| *Bettongia gaimardi* | B | Hysterangiales | Hysterangiaceae | *Hysterangium* | *Hysterangium inflatum* | *Hysterangium inflatum* | 1 | C. Johnson |
| *Bettongia gaimardi* | B | Agaricales | Strophariaceae | *Hysterogaster* | *Hysterogaster* | *Hysterogaster* | 1 | Taylor 1992 |
| *Bettongia gaimardi* | B | Agaricales | Strophariaceae | *Hysterogaster* | *Hysterogaster* | *Hysterogaster* | 1 | C. Johnson |
| *Bettongia gaimardi* | A | Pezizales | Tuberaceae | *Labyrinthomyces* | *Labyrinthomyces* | *Labyrinthomyces* | 1 | C. Johnson |
| *Bettongia gaimardi* | A | Pezizales | Tuberaceae | *Labyrinthomyces* | *Labyrinthomyces varius* | *Labyrinthomyces varius* | 1 | Taylor 1992 |
| *Bettongia gaimardi* | A | Pezizales | Tuberaceae | *Labyrinthomyces* | *Labyrinthomyces varius* | *Labyrinthomyces varius* | 1 | Taylor 1988 |
| *Bettongia gaimardi* | B | Hysterangiales | Mesophelliaceae | *Mesophellia* | *Mesophellia* | *Mesophellia* | 1 | Johnson 1994b |
| *Bettongia gaimardi* | B | Hysterangiales | Mesophelliaceae | *Mesophellia* | *Mesophellia* | *Mesophellia* | 1 | Taylor 1992 |
| *Bettongia gaimardi* | B | Hysterangiales | Mesophelliaceae | *Mesophellia* | *Mesophellia* | *Mesophellia* | 1 | Taylor 1988 |
| *Bettongia gaimardi* | B | Hysterangiales | Mesophelliaceae | *Mesophellia* | *Mesophellia pachythrix* | *Andebbia pachythrix* | 1 | Taylor 1988 |
| *Bettongia gaimardi* | B | Hysterangiales | Mesophelliaceae | *Mesophellia* | *Mesophellia tasmanica* | *Mesophellia tasmanica* | 1 | Taylor 1988 |
| *Bettongia gaimardi* | A | Hypocreales | Ceratostomataceae | *Microthecium* | *Microthecium beatonii* | *Sphaerodes beatonii* | 1 | Taylor 1988 |
| *Bettongia gaimardi* | A | Hypocreales | Ceratostomataceae | *Microthecium* | *Microthecium beatonii* | *Sphaerodes beatonii* | 1 | Taylor 1992 |
| *Bettongia gaimardi* | B | Hysterangiales | Mesophelliaceae | *Nothocastoreum* | *Nothocastoreum cretaceum* | *Nothocastoreum cretaceum* | 1 | C. Johnson |
| *Bettongia gaimardi* | B | Boletales | Octavianiaceae | *Octaviania* | *Octaviania* | *Octaviania* | 1 | Johnson 1994b |
| *Bettongia gaimardi* | A | Pezizales | Pezizaceae | *Peziza* | *Peziza whitei* | *Hydnoplicata convoluta* | 1 | Taylor 1988 |
| *Bettongia gaimardi* | B | Agaricales | Hydnangiaceae | *Podohydnangium* | *Podohydnangium australe* | *Podohydnangium australe* | 1 | Johnson 1994b |
| *Bettongia gaimardi* | B | Agaricales | Entolomataceae | *Richoniella* | *Richoniella* | *Richoniella* | 1 | Taylor 1988 |
| *Bettongia gaimardi* | B | Agaricales | Entolomataceae | *Richoniella* | *Richoniella macrosporus* | *Entoloma macrosporum* | 1 | Taylor 1992 |
| *Bettongia gaimardi* | B | Russulales | Russulaceae | *Russula* | *Russula* | *Russula* | 1 | Taylor 1992 |
| *Bettongia gaimardi* | B | Russulales | Russulaceae | *Russula* | *Russula* | *Russula* | 1 | Taylor 1988 |
| *Bettongia gaimardi* | B | Boletales | Sclerodermataceae | *Scleroderma* | *Scleroderma* | *Scleroderma* | 1 | Taylor 1988 |
| *Bettongia gaimardi* | B | Boletales | Sclerodermataceae | *Scleroderma* | *Scleroderma paradoxum* | *Scleroderma paradoxum* | 1 | Taylor 1992 |
| *Bettongia gaimardi* | B | Agaricales | Cortinariaceae | *Thaxterogaster* | *Thaxterogaster* | *Cortinarius* | 1 | Johnson 1994b |
| *Bettongia gaimardi* | B | Agaricales | Cortinariaceae | *Thaxterogaster* | *Thaxterogaster campbellae* | *Cortinarius campbelliae* | 1 | Taylor 1988 |
| *Bettongia gaimardi* | B | Agaricales | Cortinariaceae | *Thaxterogaster* | *Thaxterogaster leucocephalus* | *Cortinarius leucocephalus* | 1 | Johnson 1994b |
| *Bettongia gaimardi* | B | Agaricales | Cortinariaceae | *Thaxterogaster* | *Thaxterogaster piriformis* | *Cortinarius piriforme* | 1 | Taylor 1992 |
| *Bettongia gaimardi* | B | Agaricales | Cortinariaceae | *Thaxterogaster* | *Thaxterogaster piriformis* | *Cortinarius piriforme* | 1 | Taylor 1988 |
| *Bettongia gaimardi* | B | Agaricales | Cortinariaceae | *Thaxterogaster* | *Thaxterogaster scabrosus* | *Cortinarius scabrosus* | 1 | Johnson 1994b |
| *Bettongia gaimardi* | B | Russulales | Russulaceae | *Unknown* | *Unknown* |  | 0 | Johnson 1994b |
| *Bettongia gaimardi* | B | Hysterangiales | Mesophelliaceae | *Unknown* | *Unknown* |  | 0 | Taylor 1992 |
| *Bettongia gaimardi* | B | Geastrales | Geastraceae | *Unknown* | *Unknown* |  | 0 | Taylor 1988 |
| *Bettongia gaimardi* | G | Glomerales | Glomeraceae | *Unknown* | *Unknown* |  | 0 | Taylor 1988 |
| *Bettongia gaimardi* | B | Russulales | Russulaceae | *Zelleromyces* | *Zelleromyces* | *Zelleromyces* | 1 | Johnson 1994b |
| *Bettongia gaimardi* | B | Russulales | Russulaceae | *Zelleromyces* | *Zelleromyces australiensis* | *Zelleromyces australiensis* | 1 | Taylor 1988 |
| *Bettongia gaimardi* | B | Russulales | Russulaceae | *Zelleromyces* | *Zelleromyces glabrellus* | *Zelleromyces glabrellus* | 1 | Taylor 1992 |
| *Bettongia gaimardi* | B | Russulales | Russulaceae | *Zelleromyces* | *Zelleromyces glabrellus* | *Zelleromyces glabrellus* | 1 | Taylor 1988 |
| *Bettongia gaimardi* | B | Russulales | Russulaceae | *Zelleromyces* | *Zelleromyces majus* | *Zelleromyces majus* | 1 | Taylor 1988 |
| *Bettongia gaimardi* | B | Russulales | Russulaceae | *Zelleromyces* | *Zelleromyces malaiensis* | *Zelleromyces malaiensis* | 1 | Johnson 1994b |
| *Bettongia gaimardi* | B | Russulales | Russulaceae | *Zelleromyces* | *Zelleromyces malaiensis* | *Zelleromyces malaiensis* | 1 | Taylor 1988 |
| *Bettongia lesueur* | u | unknown | unknown | *Unknown* | *Unknown [hypogeal fungi 1] Robley et al. 2001* |  | 0 | Robley et al. 2001 |
| *Bettongia lesueur* | u | unknown | unknown | *Unknown* | *Unknown [hypogeal fungi 2] Robley et al. 2001* |  | 0 | Robley et al. 2001 |
| *Bettongia lesueur* | u | unknown | unknown | *Unknown* | *Unknown [hypogeal fungi 3] Robley et al. 2001* |  | 0 | Robley et al. 2001 |
| *Bettongia lesueur* | u | unknown | unknown | *Unknown* | *Unknown [hypogeal fungi 4] Robley et al. 2001* |  | 0 | Robley et al. 2001 |
| *Bettongia lesueur* | u | unknown | unknown | *Unknown* | *Unknown [hypogeal fungi 5] Robley et al. 2001* |  | 0 | Robley et al. 2001 |
| *Bettongia penicillata* | B | Hysterangiales | Gallaceaceae | *Australasia* | *Australasia chlorospora* | *Australasia chlorospora* | 1 | Christensen 1980 |
| *Bettongia penicillata* | B | Hysterangiales | Gallaceaceae | *Austrogautieria* | *Austrogautieria chlorospora* | *Austrogautieria chlorospora* | 1 | Lamont et al. 1985 |
| *Bettongia penicillata* | A | Pezizales | Tuberaceae | *Labyrinthomyces* | *Labyrinthomyces varius* | *Labyrinthomyces varius* | 1 | Christensen 1980 |
| *Bettongia penicillata* | A | Pezizales | Tuberaceae | *Labyrinthomyces* | *Labyrinthomyces varius* | *Labyrinthomyces varius* | 1 | Lamont et al. 1985 |
| *Bettongia penicillata* | B | Russulales | Russulaceae | *Martellia* | *Martellia* |  | 0 | Christensen 1980 |
| *Bettongia penicillata* | B | Hysterangiales | Mesophelliaceae | *Mesophellia* | *Mesophellia arenia* | *Mesophellia arenia* | 1 | Lamont et al. 1985 |
| *Bettongia penicillata* | B | Hysterangiales | Mesophelliaceae | *Mesophellia* | *Mesophellia labyrinthina* | *Mesophellia labyrinthina* | 1 | Lamont et al. 1985 |
| *Bettongia penicillata* | B | Hysterangiales | Mesophelliaceae | *Mesophellia* | *Mesophellia sp.2 [7-8 x 5 µm, hyaline and smooth]* | *Mesophellia* | 1 | Christensen 1980 |
| *Bettongia penicillata* | B | Hysterangiales | Mesophelliaceae | *Mesophellia* | *Mesophellia trabalis* | *Mesophellia trabalis* | 1 | Lamont et al. 1985 |
| *Bettongia penicillata* | B | Russulales | Russulaceae | *Unknown* | *Unknown* |  | 0 | Lamont et al. 1985 |
| *Bettongia penicillata* | u | unknown | unknown | *Unknown* | *Unknown [see paper for spore description] Lamont et al. 1985* |  | 0 | Lamont et al. 1985 |
| *Bettongia penicillata* | B | Russulales | Russulaceae | *Unknown* | *Unknown sp. nov. 1 Lamont et al. 1985* |  | 0 | Lamont et al. 1985 |
| *Bettongia penicillata* | B | Russulales | Russulaceae | *Unknown* | *Unknown sp. nov. 2 Lamont et al. 1985* |  | 0 | Lamont et al. 1985 |
| *Bettongia penicillata* | u | unknown | unknown | *Unknown* | *Unknown sp.15 Lamont et al. 1985* |  | 0 | Lamont et al. 1985 |
| *Bettongia penicillata* | u | unknown | unknown | *Unknown* | *Unknown sp.17 Lamont et al. 1985* |  | 0 | Lamont et al. 1985 |
| *Bettongia penicillata* | u | unknown | unknown | *Unknown* | *Unknown sp.37 Lamont et al. 1985* |  | 0 | Lamont et al. 1985 |
| *Bettongia penicillata* | u | unknown | unknown | *Unknown* | *Unknown sp.38 Lamont et al. 1985* |  | 0 | Lamont et al. 1985 |
| *Bettongia penicillata* | u | unknown | unknown | *Unknown* | *Unknown sp.39 Lamont et al. 1985* |  | 0 | Lamont et al. 1985 |
| *Bettongia penicillata* | u | unknown | unknown | *Unknown* | *Unknown sp.40 Lamont et al. 1985* |  | 0 | Lamont et al. 1985 |
| *Bettongia penicillata* | u | unknown | unknown | *Unknown* | *Unknown sp.41 Lamont et al. 1985* |  | 0 | Lamont et al. 1985 |
| *Bettongia penicillata* | u | unknown | unknown | *Unknown* | *Unknown sp.42 Lamont et al. 1985* |  | 0 | Lamont et al. 1985 |
| *Bettongia penicillata* | u | unknown | unknown | *Unknown* | *Unknown spore type 1 Christensen 1980* |  | 0 | Christensen 1980 |
| *Bettongia penicillata* | u | unknown | unknown | *Unknown* | *Unknown spore type 11 Christensen 1980* |  | 0 | Christensen 1980 |
| *Bettongia penicillata* | u | unknown | unknown | *Unknown* | *Unknown spore type 12 Christensen 1980* |  | 0 | Christensen 1980 |
| *Bettongia penicillata* | u | unknown | unknown | *Unknown* | *Unknown spore type 14 Christensen 1980* |  | 0 | Christensen 1980 |
| *Bettongia penicillata* | u | unknown | unknown | *Unknown* | *Unknown spore type 15 Christensen 1980* |  | 0 | Christensen 1980 |
| *Bettongia penicillata* | u | unknown | unknown | *Unknown* | *Unknown spore type 17 Christensen 1980* |  | 0 | Christensen 1980 |
| *Bettongia penicillata* | u | unknown | unknown | *Unknown* | *Unknown spore type 28 Christensen 1980* |  | 0 | Christensen 1980 |
| *Bettongia penicillata* | u | unknown | unknown | *Unknown* | *Unknown spore type 29 Christensen 1980* |  | 0 | Christensen 1980 |
| *Bettongia penicillata* | u | unknown | unknown | *Unknown* | *Unknown spore type 32 Christensen 1980* |  | 0 | Christensen 1980 |
| *Bettongia penicillata* | u | unknown | unknown | *Unknown* | *Unknown spore type 33 Christensen 1980* |  | 0 | Christensen 1980 |
| *Bettongia penicillata* | u | unknown | unknown | *Unknown* | *Unknown spore type 37 Christensen 1980* |  | 0 | Christensen 1980 |
| *Bettongia penicillata* | u | unknown | unknown | *Unknown* | *Unknown spore type 38 Christensen 1980* |  | 0 | Christensen 1980 |
| *Bettongia penicillata* | u | unknown | unknown | *Unknown* | *Unknown spore type 39 Christensen 1980* |  | 0 | Christensen 1980 |
| *Bettongia penicillata* | u | unknown | unknown | *Unknown* | *Unknown spore type 40 Christensen 1980* |  | 0 | Christensen 1980 |
| *Bettongia penicillata* | u | unknown | unknown | *Unknown* | *Unknown spore type 41 Christensen 1980* |  | 0 | Christensen 1980 |
| *Bettongia penicillata* | u | unknown | unknown | *Unknown* | *Unknown spore type 42 Christensen 1980* |  | 0 | Christensen 1980 |
| *Bettongia penicillata* | u | unknown | unknown | *Unknown* | *Unknown spore type 44 Christensen 1980* |  | 0 | Christensen 1980 |
| *Bettongia penicillata* | u | unknown | unknown | *Unknown* | *Unknown spore type 45 Christensen 1980* |  | 0 | Christensen 1980 |
| *Bettongia penicillata* | u | unknown | unknown | *Unknown* | *Unknown spore type 46 Christensen 1980* |  | 0 | Christensen 1980 |
| *Bettongia penicillata* | B | Boletales | Octavianiaceae | *Wakefieldia* | *Wakefieldia* | *Wakefieldia* | 1 | Lamont et al. 1985 |
| *Bettongia tropica* | A | Pezizales | Pezizaceae | *Amylascus* | *Amylascus* | *Amylascus* | 1 | Vernes et al. 2001 |
| *Bettongia tropica* | B | Agaricales | Cortinariaceae | *Aroramyces* | *Aroramyces queenslandica* | *Aroramyces queenslandica* | 1 | Vernes et al. 2001 |
| *Bettongia tropica* | B | Agaricales | Cortinariaceae | *Aroramyces* | *Aroramyces sp.1 Vernes, K* | *Aroramyces* | 1 | Vernes et al. 2001 |
| *Bettongia tropica* | B | Agaricales | Cortinariaceae | *Aroramyces* | *Aroramyces sp.2 Vernes, K* | *Aroramyces* | 1 | Vernes et al. 2001 |
| *Bettongia tropica* | B | Hysterangiales | Gallaceaceae | *Austrogautieria* | *Austrogautieria chlorospora* | *Austrogautieria chlorospora* | 1 | Vernes et al. 2001 |
| *Bettongia tropica* | B | Hysterangiales | Gallaceaceae | *Austrogautieria* | *Austrogautieria longispora nom. ined.* | *Austrogautieria longispora nom. ined.* | 1 | Vernes et al. 2001 |
| *Bettongia tropica* | u | unknown | unknown | *Beatonia* | *Beatonia* | *Beatonia* | 1 | Vernes et al. 2001 |
| *Bettongia tropica* | B | Hysterangiales | Mesophelliaceae | *Castoreum* | *Castoreum* | *Castoreum* | 1 | Reddell et al. 1997 |
| *Bettongia tropica* | B | Boletales | Boletaceae | *Chamonixia* | *Chamonixia* | *Rossbeevera* | 1 | Reddell et al. 1997 |
| *Bettongia tropica* | B | Hysterangiales | Mesophelliaceae | *Chondrogaster* | *Chondrogaster* | *Chondrogaster* | 1 | Reddell et al. 1997 |
| *Bettongia tropica* | B | Agaricales | Cortinariaceae | *Descomyces* | *Descomyces* | *Descomyces* | 1 | Vernes et al. 2001 |
| *Bettongia tropica* | A | Elaphomycetales | Elaphomycetaceae | *Elaphomyces* | *Elaphomyces* | *Elaphomyces* | 1 | Reddell et al. 1997 |
| *Bettongia tropica* | A | Elaphomycetales | Elaphomycetaceae | *Elaphomyces* | *Elaphomyces sp.1/10 Vernes, K* | *Elaphomyces* | 1 | Vernes et al. 2001 |
| *Bettongia tropica* | A | Elaphomycetales | Elaphomycetaceae | *Elaphomyces* | *Elaphomyces sp.11 Vernes, K* | *Elaphomyces* | 1 | Vernes et al. 2001 |
| *Bettongia tropica* | A | Elaphomycetales | Elaphomycetaceae | *Elaphomyces* | *Elaphomyces sp.2/7 Vernes, K* | *Elaphomyces* | 1 | Vernes et al. 2001 |
| *Bettongia tropica* | A | Elaphomycetales | Elaphomycetaceae | *Elaphomyces* | *Elaphomyces sp.3 Vernes, K* | *Elaphomyces* | 1 | Vernes et al. 2001 |
| *Bettongia tropica* | A | Elaphomycetales | Elaphomycetaceae | *Elaphomyces* | *Elaphomyces sp.4 Vernes, K* | *Elaphomyces* | 1 | Vernes et al. 2001 |
| *Bettongia tropica* | A | Elaphomycetales | Elaphomycetaceae | *Elaphomyces* | *Elaphomyces sp.5/6 Vernes, K* | *Elaphomyces* | 1 | Vernes et al. 2001 |
| *Bettongia tropica* | A | Elaphomycetales | Elaphomycetaceae | *Elaphomyces* | *Elaphomyces sp.8/9 Vernes, K* | *Elaphomyces* | 1 | Vernes et al. 2001 |
| *Bettongia tropica* | Z | Endogonales | Endogonaceae | *Endogone* | *Endogone* | *Endogone* | 1 | Reddell et al. 1997 |
| *Bettongia tropica* | B | Hysterangiales | Gallaceaceae | *Gallacea* | *Gallacea* | *Gallacea* | 1 | Vernes et al. 2001 |
| *Bettongia tropica* | B | Gomphales | Gomphaceae | *Gautieria* | *Gautieria sp. nov.1 Vernes, K* | *Gautieria* | 1 | Vernes et al. 2001 |
| *Bettongia tropica* | B | Gomphales | Gomphaceae | *Gautieria* | *Gautieria sp. nov.2 Vernes, K* | *Gautieria* | 1 | Vernes et al. 2001 |
| *Bettongia tropica* | G | Glomerales | Glomeraceae | *Glomus* | *Glomus* | *Glomus* | 1 | Vernes et al. 2001 |
| *Bettongia tropica* | B | Hysterangiales | Mesophelliaceae | *Gummiglobus* | *Gummiglobus* | *Gummiglobus* | 1 | Reddell et al. 1997 |
| *Bettongia tropica* | B | Agaricales | Hydnangiaceae | *Hydnangium* | *Hydnangium* | *Hydnangium* | 1 | Vernes et al. 2001 |
| *Bettongia tropica* | B | Hysterangiales | Hysterangiaceae | *Hysterangium* | *Hysterangium* | *Hysterangium* | 1 | Reddell et al. 1997 |
| *Bettongia tropica* | B | Hysterangiales | Hysterangiaceae | *Hysterangium* | *Hysterangium* | *Hysterangium* | 1 | Vernes et al. 2001 |
| *Bettongia tropica* | B | Hysterangiales | Hysterangiaceae | *Hysterangium* | *Hysterangium sp.2 Vernes, K* | *Hysterangium* | 1 | Vernes et al. 2001 |
| *Bettongia tropica* | B | Agaricales | Strophariaceae | *Hysterogaster* | *Hysterogaster* | *Hysterogaster* | 1 | Vernes et al. 2001 |
| *Bettongia tropica* | B | Hysterangiales | Mesophelliaceae | *Mesophellia* | *Mesophellia [large spore] Vernes, K* | *Mesophellia* | 1 | Vernes et al. 2001 |
| *Bettongia tropica* | B | Boletales | Boletaceae | *Mycoamaranthus* | *Mycoamaranthus auriorbis* | *Mycoamaranthus auriorbis* | 1 | Reddell et al. 1997 |
| *Bettongia tropica* | B | unknown | unknown | *Pseudohysterangium* | *Pseudohysterangium* | *Pseudohysterangium* | 1 | Reddell et al. 1997 |
| *Bettongia tropica* | B | Boletales | Boletaceae | *Royoungia* | *Royoungia boletoides* | *Royoungia boletoides* | 1 | Reddell et al. 1997 |
| *Bettongia tropica* | B | Boletales | Sclerodermataceae | *Scleroderma* | *Scleroderma* | *Scleroderma* | 1 | Reddell et al. 1997 |
| *Bettongia tropica* | B | Boletales | Sclerodermataceae | *Scleroderma* | *Scleroderma* | *Scleroderma* | 1 | Vernes et al. 2001 |
| *Bettongia tropica* | B | Geastrales | Geastraceae | *Sclerogaster* | *Sclerogaster* | *Sclerogaster* | 1 | Reddell et al. 1997 |
| *Bettongia tropica* | B | Agaricales | Cortinariaceae | *Thaxterogaster* | *Thaxterogaster sp.1 Vernes, K* | *Cortinarius* | 1 | Vernes et al. 2001 |
| *Bettongia tropica* | B | Agaricales | Cortinariaceae | *Thaxterogaster* | *Thaxterogaster sp.2 Vernes, K* | *Cortinarius* | 1 | Vernes et al. 2001 |
| *Bettongia tropica* | B | Agaricales | Cortinariaceae | *Thaxterogaster* | *Thaxterogaster sp.3 Vernes, K* | *Cortinarius* | 1 | Vernes et al. 2001 |
| *Bettongia tropica* | B | Agaricales | unknown | *Timgrovea* | *Timgrovea* | *Timgrovea* | 1 | Vernes et al. 2001 |
| *Bettongia tropica* | A | Pezizales | Pezizaceae | *Unknown* | *Unknown* |  | 0 | Reddell et al. 1997 |
| *Bettongia tropica* | A | Pezizales | Pyronemataceae | *Unknown* | *Unknown* |  | 0 | Reddell et al. 1997 |
| *Bettongia tropica* | B | unknown | unknown | *Unknown* | *Unknown* |  | 0 | Reddell et al. 1997 |
| *Bettongia tropica* | B | Agaricales | Cortinariaceae | *Unknown* | *Unknown* |  | 0 | Reddell et al. 1997 |
| *Bettongia tropica* | B | Agaricales | Gautieriaceae | *Unknown* | *Unknown* |  | 0 | Reddell et al. 1997 |
| *Bettongia tropica* | B | Hysterangiales | Mesophelliaceae | *Unknown* | *Unknown* |  | 0 | Reddell et al. 1997 |
| *Bettongia tropica* | B | Agaricales | Russulaceae | *Unknown* | *Unknown* |  | 0 | Reddell et al. 1997 |
| *Bettongia tropica* | A | Pezizales | Tuberaceae | *Unknown* | *Unknown* |  | 0 | Vernes et al. 2001 |
| *Bettongia tropica* | B | Russulales | unknown | *Unknown* | *Unknown* |  | 0 | Vernes et al. 2001 |
| *Bettongia tropica* | B | Hysterangiales | Mesophelliaceae | *Unknown* | *Unknown* |  | 0 | Vernes et al. 2001 |
| *Bettongia tropica* | u | unknown | unknown | *Unknown* | *Unknown sp.1 Vernes, K* |  | 0 | Vernes et al. 2001 |
| *Bettongia tropica* | B | Russulales | Russulaceae | *Zelleromyces* | *Zelleromyces* | *Zelleromyces* | 1 | Vernes et al. 2001 |
| *Echymipera rufescens australis* | B | Agaricales | Cortinariaceae | *Cortinarius* | *Cortinarius* | *Cortinarius* | 1 | Shevill and Johnson 2008 |
| *Echymipera rufescens australis* | G | Glomerales | Glomeraceae | *Glomus* | *Glomus* | *Glomus* | 1 | Shevill and Johnson 2008 |
| *Echymipera rufescens australis* | B | Hysterangiales | Hysterangiaceae | *Hysterangium* | *Hysterangium* | *Hysterangium* | 1 | Shevill and Johnson 2008 |
| *Hypsiprymnodon moschatus* | B | Agaricales | Russulaceae | *Unknown* | *Unknown* |  | 0 | Reddell et al. 1997 |
| *Isoodon macrourus* | B | Hysterangiales | Mesophelliaceae | *Chondrogaster* | *Chondrogaster* | *Chondrogaster* | 1 | Reddell et al. 1997 |
| *Isoodon macrourus* | A | Elaphomycetales | Elaphomycetaceae | *Elaphomyces* | *Elaphomyces* | *Elaphomyces* | 1 | Reddell et al. 1997 |
| *Isoodon macrourus* | Z | Endogonales | Endogonaceae | *Endogone* | *Endogone* | *Endogone* | 1 | Reddell et al. 1997 |
| *Isoodon macrourus* | B | Hysterangiales | Mesophelliaceae | *Gummiglobus* | *Gummiglobus* | *Gummiglobus* | 1 | Reddell et al. 1997 |
| *Isoodon macrourus* | B | Hysterangiales | Hysterangiaceae | *Hysterangium* | *Hysterangium* | *Hysterangium* | 1 | Reddell et al. 1997 |
| *Isoodon macrourus* | B | unknown | unknown | *Pseudohysterangium* | *Pseudohysterangium* | *Pseudohysterangium* | 1 | Reddell et al. 1997 |
| *Isoodon macrourus* | B | Boletales | Sclerodermataceae | *Scleroderma* | *Scleroderma* | *Scleroderma* | 1 | Reddell et al. 1997 |
| *Isoodon macrourus* | B | Geastrales | Geastraceae | *Sclerogaster* | *Sclerogaster* | *Sclerogaster* | 1 | Reddell et al. 1997 |
| *Isoodon macrourus* | A | Pezizales | Pyronemataceae | *Unknown* | *Unknown* |  | 0 | Reddell et al. 1997 |
| *Isoodon macrourus* | B | unknown | unknown | *Unknown* | *Unknown* |  | 0 | Reddell et al. 1997 |
| *Isoodon macrourus* | B | Agaricales | Cortinariaceae | *Unknown* | *Unknown* |  | 0 | Reddell et al. 1997 |
| *Isoodon macrourus* | B | Agaricales | Gautieriaceae | *Unknown* | *Unknown* |  | 0 | Reddell et al. 1997 |
| *Isoodon macrourus* | B | Hysterangiales | Mesophelliaceae | *Unknown* | *Unknown* |  | 0 | Reddell et al. 1997 |
| *Isoodon macrourus* | B | Agaricales | Russulaceae | *Unknown* | *Unknown* |  | 0 | Reddell et al. 1997 |
| *Isoodon obesulus* | B | Hysterangiales | Gallaceaceae | *Australasia* | *Australasia chlorospora* | *Australasia chlorospora* | 1 | Christensen 1980 |
| *Isoodon obesulus* | B | Boletales | Boletaceae | *Chamonixia* | *Chamonixia mucosa* | *Rossbeevera mucosa* | 1 | T. May |
| *Isoodon obesulus* | B | Agaricales | Strophariaceae | *Galerina* | *Galerina* | *Galerina* | 1 | T. May |
| *Isoodon obesulus* | B | Hysterangiales | Hysterangiaceae | *Hysterangium* | *Hysterangium* | *Hysterangium* | 1 | T. May |
| *Isoodon obesulus* | A | Pezizales | Tuberaceae | *Labyrinthomyces* | *Labyrinthomyces varius* | *Labyrinthomyces varius* | 1 | Christensen 1980 |
| *Isoodon obesulus* | B | Hysterangiales | Mesophelliaceae | *Mesophellia* | *Mesophellia* | *Mesophellia* | 1 | Claridge et al. 1991 |
| *Isoodon obesulus* | B | Hysterangiales | Mesophelliaceae | *Mesophellia* | *Mesophellia sp.2 [7-8 x 5 µm, hyaline and smooth]* | *Mesophellia* | 1 | Christensen 1980 |
| *Isoodon obesulus* | u | unknown | unknown | *Unknown* | *Unknown sp. B Claridge, A W* |  | 0 | Claridge et al. 1991 |
| *Isoodon obesulus* | u | unknown | unknown | *Unknown* | *Unknown sp. C Claridge, A W* |  | 0 | Claridge et al. 1991 |
| *Isoodon obesulus* | u | unknown | unknown | *Unknown* | *Unknown spore type 1 Christensen 1980* |  | 0 | Christensen 1980 |
| *Isoodon obesulus* | u | unknown | unknown | *Unknown* | *Unknown spore type 43 Christensen 1980* |  | 0 | Christensen 1980 |
| *Isoodon obesulus peninsulae* | B | Agaricales | Cortinariaceae | *Aroramyces* | *Aroramyces* | *Aroramyces* | 1 | Keiper and Johnson 2004 |
| *Isoodon obesulus peninsulae* | B | Hysterangiales | Gallaceaceae | *Austrogautieria* | *Austrogautieria* | *Austrogautieria* | 1 | Keiper and Johnson 2004 |
| *Isoodon obesulus peninsulae* | B | Gomphales | Gomphaceae | *Gautieria* | *Gautieria* | *Gautieria* | 1 | Keiper and Johnson 2004 |
| *Isoodon obesulus peninsulae* | G | Glomerales | Glomeraceae | *Glomus* | *Glomus* | *Glomus* | 1 | Keiper and Johnson 2004 |
| *Isoodon obesulus peninsulae* | B | Hysterangiales | Hysterangiaceae | *Hysterangium* | *Hysterangium* | *Hysterangium* | 1 | Keiper and Johnson 2004 |
| *Isoodon obesulus peninsulae* | B | Agaricales | Strophariaceae | *Hysterogaster* | *Hysterogaster* | *Hysterogaster* | 1 | Keiper and Johnson 2004 |
| *Isoodon obesulus peninsulae* | B | unknown | unknown | *Pogisperma* | *Pogisperma* | *Pogisperma* | 1 | Keiper and Johnson 2004 |
| *Isoodon obesulus peninsulae* | B | Agaricales | Cortinariaceae | *Protoglossum* | *Protoglossum* | *Cortinarius* | 1 | Keiper and Johnson 2004 |
| *Isoodon obesulus peninsulae* | B | Agaricales | Cortinariaceae | *Thaxterogaster* | *Thaxterogaster* | *Cortinarius* | 1 | Keiper and Johnson 2004 |
| *Macropus parma* | B | Agaricales | Agaricaceae | *Agaricus* | *Agaricus sp.1 Vernes, K* | *Agaricus* | 1 | Vernes 2010 |
| *Macropus parma* | B | Agaricales | Cortinariaceae | *Aroramyces* | *Aroramyces sp.1 Vernes, K* | *Aroramyces* | 1 | Vernes 2010 |
| *Macropus parma* | B | Agaricales | Cortinariaceae | *Aroramyces* | *Aroramyces sp.2 Vernes, K* | *Aroramyces* | 1 | Vernes 2010 |
| *Macropus parma* | B | Hysterangiales | Gallaceaceae | *Austrogautieria* | *Austrogautieria* | *Austrogautieria* | 1 | Vernes 2010 |
| *Macropus parma* | B | Boletales | Boletaceae | *Boletellus* | *Boletellus* | *Boletellus* | 1 | Vernes 2010 |
| *Macropus parma* | B | Boletales | Boletaceae | *Chamonixia* | *Chamonixia* | *Rossbeevera* | 1 | Vernes 2010 |
| *Macropus parma* | A | Pezizales | Tuberaceae | *Dingleya* | *Dingleya* | *Dingleya* | 1 | Vernes 2010 |
| *Macropus parma* | A | Elaphomycetales | Elaphomycetaceae | *Elaphomyces* | *Elaphomyces* | *Elaphomyces* | 1 | Vernes 2010 |
| *Macropus parma* | B | Gomphales | Gomphaceae | *Gautieria* | *Gautieria* | *Gautieria* | 1 | Vernes 2010 |
| *Macropus parma* | B | Hysterangiales | Hysterangiaceae | *Hysterangium* | *Hysterangium* | *Hysterangium* | 1 | Vernes 2010 |
| *Macropus parma* | B | Agaricales | Strophariaceae | *Hysterogaster* | *Hysterogaster sp.2 Vernes, K* | *Hysterogaster* | 1 | Vernes 2010 |
| *Macropus parma* | A | Pezizales | Tuberaceae | *Labyrinthomyces* | *Labyrinthomyces sp.1 Vernes, K* | *Labyrinthomyces* | 1 | Vernes 2010 |
| *Macropus parma* | A | Pezizales | Tuberaceae | *Labyrinthomyces* | *Labyrinthomyces sp.2 Vernes, K* | *Labyrinthomyces* | 1 | Vernes 2010 |
| *Macropus parma* | B | Hysterangiales | Mesophelliaceae | *Mesophellia* | *Mesophellia* | *Mesophellia* | 1 | Vernes 2010 |
| *Macropus parma* | B | Boletales | Octavianiaceae | *Octaviania* | *Octaviania* | *Octaviania* | 1 | Vernes 2010 |
| *Macropus parma* | B | Boletales | Sclerodermataceae | *Scleroderma* | *Scleroderma sp.1 Vernes, K* | *Scleroderma* | 1 | Vernes 2010 |
| *Macropus parma* | B | Boletales | Sclerodermataceae | *Scleroderma* | *Scleroderma sp.2 Vernes, K* | *Scleroderma* | 1 | Vernes 2010 |
| *Macropus parma* | B | Boletales | Sclerodermataceae | *Scleroderma* | *Scleroderma sp.3 Vernes, K* | *Scleroderma* | 1 | Vernes 2010 |
| *Macropus parma* | B | Boletales | Sclerodermataceae | *Scleroderma* | *Scleroderma tommayi* | *Scleroderma tommayi* | 1 | Vernes 2010 |
| *Macropus parma* | B | Agaricales | Cortinariaceae | *Thaxterogaster* | *Thaxterogaster* | *Cortinarius* | 1 | Vernes 2010 |
| *Macropus parma* | B | Hysterangiales | Mesophelliaceae | *Unknown* | *Unknown* |  | 0 | Vernes 2010 |
| *Macropus parma* | B | Agaricales | Strophariaceae | *Unknown* | *Unknown* |  | 0 | Vernes 2010 |
| *Macropus parma* | B | Boletales | unknown | *Unknown* | *Unknown sp.1 Vernes, K* |  | 0 | Vernes 2010 |
| *Macropus parma* | u | unknown | unknown | *Unknown* | *Unknown sp.1 Vernes, K* |  | 0 | Vernes 2010 |
| *Macropus parma* | B | Boletales | unknown | *Unknown* | *Unknown sp.2 Vernes, K* |  | 0 | Vernes 2010 |
| *Macropus parma* | B | Russulales | unknown | *Unknown* | *Unknown sp.2 Vernes, K* |  | 0 | Vernes 2010 |
| *Macropus parma* | u | unknown | unknown | *Unknown* | *Unknown sp.2 Vernes, K* |  | 0 | Vernes 2010 |
| *Macropus parma* | B | Russulales | unknown | *Unknown* | *Unknown sp.3 Vernes, K* |  | 0 | Vernes 2010 |
| *Macropus parma* | B | Russulales | unknown | *Unknown* | *Unknown sp.4 Vernes, K* |  | 0 | Vernes 2010 |
| *Macropus parma* | B | Russulales | unknown | *Unknown* | *Unknown sp.5 Vernes, K* |  | 0 | Vernes 2010 |
| *Macrotis lagotis* | G | Diversisporales | Acaulosporaceae | *Acaulospora* | *Acaulospora laevis* | *Acaulospora laevis* | 1 | Navnith et al. 2009 |
| *Macrotis lagotis* | u | unknown | unknown | *Unknown* | *Unknown* |  | 0 | Navnith et al. 2009 |
| *Melomys cervinipes* | B | Agaricales | Agaricaceae | *Agaricus* | *Agaricus* | *Agaricus* | 1 | Vernes et al. 2015 |
| *Melomys cervinipes* | A | Pezizales | Pezizaceae | *Amylascus* | *Amylascus* | *Amylascus* | 1 | Vernes et al. 2015 |
| *Melomys cervinipes* | B | Agaricales | Cortinariaceae | *Aroramyces* | *Aroramyces sp.1 Vernes, K* | *Aroramyces* | 1 | Vernes et al. 2015 |
| *Melomys cervinipes* | B | Hysterangiales | Gallaceaceae | *Austrogautieria* | *Austrogautieria* | *Austrogautieria* | 1 | Vernes et al. 2015 |
| *Melomys cervinipes* | B | Boletales | Boletaceae | *Chamonixia* | *Chamonixia* | *Rossbeevera* | 1 | Vernes et al. 2015 |
| *Melomys cervinipes* | B | Agaricales | Cortinariaceae | *Cortinarius* | *Cortinarius* | *Cortinarius* | 1 | Vernes et al. 2015 |
| *Melomys cervinipes* | B | Agaricales | Cortinariaceae | *Descomyces* | *Descomyces sp.2 Vernes, K* | *Descomyces* | 1 | Vernes et al. 2015 |
| *Melomys cervinipes* | B | Agaricales | Cortinariaceae | *Descomyces* | *Descomyces sp.3 Vernes, K* | *Descomyces* | 1 | Vernes et al. 2015 |
| *Melomys cervinipes* | G | Glomerales | Glomeraceae | *Glomus* | *Glomus* | *Glomus* | 1 | Vernes et al. 2015 |
| *Melomys cervinipes* | B | Agaricales | Hydnangiaceae | *Hydnangium* | *Hydnangium* | *Hydnangium* | 1 | Vernes et al. 2015 |
| *Melomys cervinipes* | B | Hysterangiales | Hysterangiaceae | *Hysterangium* | *Hysterangium* | *Hysterangium* | 1 | Vernes et al. 2015 |
| *Melomys cervinipes* | B | Agaricales | Strophariaceae | *Hysterogaster* | *Hysterogaster sp.2 Vernes, K* | *Hysterogaster* | 1 | Vernes et al. 2015 |
| *Melomys cervinipes* | B | Agaricales | Strophariaceae | *Hysterogaster* | *Hysterogaster sp.3 Vernes, K* | *Hysterogaster* | 1 | Vernes et al. 2015 |
| *Melomys cervinipes* | B | Russulales | Albatrellaceae | *Leucogaster* | *Leucogaster* | *Leucogaster* | 1 | Vernes et al. 2015 |
| *Melomys cervinipes* | B | Boletales | Sclerodermataceae | *Scleroderma* | *Scleroderma* | *Scleroderma* | 1 | Vernes et al. 2015 |
| *Melomys cervinipes* | B | Agaricales | Cortinariaceae | *Thaxterogaster* | *Thaxterogaster sp.3 Vernes, K* | *Cortinarius* | 1 | Vernes et al. 2015 |
| *Melomys cervinipes* | u | unknown | unknown | *Unknown* | *Unknown* |  | 0 | Vernes et al. 2015 |
| *Melomys cervinipes* | B | Russulales | unknown | *Unknown* | *Unknown sp.1 Vernes, K* |  | 0 | Vernes et al. 2015 |
| *Melomys cervinipes* | u | unknown | unknown | *Unknown* | *Unknown sp.1 Vernes, K* |  | 0 | Vernes et al. 2015 |
| *Melomys cervinipes* | u | unknown | unknown | *Unknown* | *Unknown sp.11 Vernes, K* |  | 0 | Vernes et al. 2015 |
| *Melomys cervinipes* | u | unknown | unknown | *Unknown* | *Unknown sp.12 Vernes, K* |  | 0 | Vernes et al. 2015 |
| *Melomys cervinipes* | A | unknown | unknown | *Unknown* | *Unknown sp.2 Vernes, K* |  | 0 | Vernes et al. 2015 |
| *Melomys cervinipes* | B | Russulales | unknown | *Unknown* | *Unknown sp.2 Vernes, K* |  | 0 | Vernes et al. 2015 |
| *Melomys cervinipes* | u | unknown | unknown | *Unknown* | *Unknown sp.3 Vernes, K* |  | 0 | Vernes et al. 2015 |
| *Mus musculus* | G | Glomerales | Glomeraceae | *Glomus* | *Glomus* | *Glomus* | 1 | Tann et al. 1991 |
| *Mus musculus* | u | unknown | unknown | *Unknown* | *Unknown [ectotropic mycorrhizal fungus] Tann et al. 1991* |  | 0 | Tann et al. 1991 |
| *Perameles nasuta* | A | Pezizales | Pezizaceae | *Amylascus* | *Amylascus* | *Amylascus* | 1 | Vernes 2014 |
| *Perameles nasuta* | B | Agaricales | Cortinariaceae | *Aroramyces* | *Aroramyces sp.1 Vernes, K* | *Aroramyces* | 1 | Vernes 2014 |
| *Perameles nasuta* | B | Hysterangiales | Gallaceaceae | *Austrogautieria* | *Austrogautieria* | *Austrogautieria* | 1 | Vernes 2014 |
| *Perameles nasuta* | B | Hysterangiales | Mesophelliaceae | *Castoreum* | *Castoreum* | *Castoreum* | 1 | Claridge 1993 |
| *Perameles nasuta* | B | Boletales | Boletaceae | *Chamonixia* | *Chamonixia* | *Rossbeevera* | 1 | Claridge 1993 |
| *Perameles nasuta* | B | Boletales | Boletaceae | *Chamonixia* | *Chamonixia* | *Rossbeevera* | 1 | Vernes 2014 |
| *Perameles nasuta* | B | Boletales | Boletaceae | *Chamonixia* | *Chamonixia vittatispora* | *Rossbeevera vittatispora* | 1 | Claridge 1993 |
| *Perameles nasuta* | B | Agaricales | Cortinariaceae | *Cortinarius* | *Cortinarius* | *Cortinarius* | 1 | Vernes 2014 |
| *Perameles nasuta* | B | Agaricales | Cortinariaceae | *Descomyces* | *Descomyces sp.2 Vernes, K* | *Descomyces* | 1 | Vernes 2014 |
| *Perameles nasuta* | B | Agaricales | Cortinariaceae | *Descomyces* | *Descomyces sp.3 Vernes, K* | *Descomyces* | 1 | Vernes 2014 |
| *Perameles nasuta* | B | Agaricales | Cortinariaceae | *Descomyces* | *Descomyces sp.5 Vernes, K* | *Descomyces* | 1 | Vernes 2014 |
| *Perameles nasuta* | B | Agaricales | Cortinariaceae | *Descomyces* | *Descomyces stolatus* | *Descomyces stolatus* | 1 | Vernes 2014 |
| *Perameles nasuta* | A | Elaphomycetales | Elaphomycetaceae | *Elaphomyces* | *Elaphomyces* | *Elaphomyces* | 1 | Vernes 2014 |
| *Perameles nasuta* | Z | Endogonales | Endogonaceae | *Endogone* | *Endogone* | *Endogone* | 1 | Reddell et al. 1997 |
| *Perameles nasuta* | Z | Endogonales | Endogonaceae | *Endogone* | *Endogone [spore walls single-layered] Claridge, A W* | *Endogone* | 1 | Claridge 1993 |
| *Perameles nasuta* | Z | Endogonales | Endogonaceae | *Endogone* | *Endogone aggregata* | *Endogone aggregata* | 1 | Thums et al. 2005 |
| *Perameles nasuta* | B | Agaricales | Agaricaceae | *Endoptychum* | *Endoptychum* |  | 0 | Vernes 2014 |
| *Perameles nasuta* | B | Gomphales | Gomphaceae | *Gautieria* | *Gautieria* | *Gautieria* | 1 | Vernes 2014 |
| *Perameles nasuta* | B | Gomphales | Gomphaceae | *Gautieria* | *Gautieria monospora* | *Gautieria monospora* | 1 | Claridge 1993 |
| *Perameles nasuta* | B | Gomphales | Gomphaceae | *Gautieria* | *Gautieria sp.1 Claridge, A W* | *Gautieria* | 1 | Claridge 1993 |
| *Perameles nasuta* | B | Gomphales | Gomphaceae | *Gautieria* | *Gautieria sp.2 Claridge, A W* | *Gautieria* | 1 | Claridge 1993 |
| *Perameles nasuta* | G | Glomerales | Glomeraceae | *Glomus* | *Glomus* | *Glomus* | 1 | Vernes 2014 |
| *Perameles nasuta* | G | Glomerales | Glomeraceae | *Glomus* | *Glomus fuegianum* | *Glomus fuegianum* | 1 | McGee and Baczocha 1994 |
| *Perameles nasuta* | G | Glomerales | Glomeraceae | *Glomus* | *Glomus fuegianum* | *Glomus fuegianum* | 1 | Scott et al. 1999 |
| *Perameles nasuta* | G | Glomerales | Glomeraceae | *Glomus* | *Glomus macrocarpus* | *Glomus macrocarpum* | 1 | McGee and Baczocha 1994 |
| *Perameles nasuta* | G | Glomerales | Glomeraceae | *Glomus* | *Glomus nigrauva* | *Glomus nigrauva* | 1 | Thums et al. 2005 |
| *Perameles nasuta* | B | Hysterangiales | Mesophelliaceae | *Gummiglobus* | *Gummiglobus* | *Gummiglobus* | 1 | Reddell et al. 1997 |
| *Perameles nasuta* | B | Agaricales | Hydnangiaceae | *Hydnangium* | *Hydnangium* | *Hydnangium* | 1 | Claridge 1993 |
| *Perameles nasuta* | B | Agaricales | Hydnangiaceae | *Hydnangium* | *Hydnangium* | *Hydnangium* | 1 | Vernes 2014 |
| *Perameles nasuta* | A | Pezizales | Pezizaceae | *Hydnoplicata* | *Hydnoplicata convoluta* | *Hydnoplicata convoluta* | 1 | Vernes 2014 |
| *Perameles nasuta* | B | Agaricales | Strophariaceae | *Hymenogaster* | *Hymenogaster albus* | *Descomyces albus* | 1 | Claridge 1993 |
| *Perameles nasuta* | B | Agaricales | Strophariaceae | *Hymenogaster* | *Hymenogaster atratus* | *Cortinarius atratus* | 1 | Claridge 1993 |
| *Perameles nasuta* | B | Agaricales | Strophariaceae | *Hymenogaster* | *Hymenogaster inflatum* | *Hymenogaster inflatum* | 1 | Claridge 1993 |
| *Perameles nasuta* | B | Agaricales | Strophariaceae | *Hymenogaster* | *Hymenogaster nanus* | *Hymenogaster nanus* | 1 | Claridge 1993 |
| *Perameles nasuta* | B | Agaricales | Strophariaceae | *Hymenogaster* | *Hymenogaster sp.1 Claridge, A W* | *Hymenogaster* | 1 | Claridge 1993 |
| *Perameles nasuta* | B | Agaricales | Strophariaceae | *Hymenogaster* | *Hymenogaster sp.2 Claridge, A W* | *Hymenogaster* | 1 | Claridge 1993 |
| *Perameles nasuta* | B | Agaricales | Strophariaceae | *Hymenogaster* | *Hymenogaster zeylanicus* | *Descomyces albellus* | 1 | Claridge 1993 |
| *Perameles nasuta* | B | Hysterangiales | Hysterangiaceae | *Hysterangium* | *Hysterangium* | *Hysterangium* | 1 | Reddell et al. 1997 |
| *Perameles nasuta* | B | Hysterangiales | Hysterangiaceae | *Hysterangium* | *Hysterangium* | *Hysterangium* | 1 | Vernes 2014 |
| *Perameles nasuta* | B | Agaricales | Strophariaceae | *Hysterogaster* | *Hysterogaster sp.2 Vernes, K* | *Hysterogaster* | 1 | Vernes 2014 |
| *Perameles nasuta* | B | Agaricales | Strophariaceae | *Hysterogaster* | *Hysterogaster sp.3 Vernes, K* | *Hysterogaster* | 1 | Vernes 2014 |
| *Perameles nasuta* | A | Pezizales | Pyronemataceae | *Jafneadelphus* | *Jafneadelphus* | *Jafneadelphus* | 1 | Claridge 1993 |
| *Perameles nasuta* | A | Pezizales | Tuberaceae | *Labyrinthomyces* | *Labyrinthomyces varius* | *Labyrinthomyces varius* | 1 | Claridge 1993 |
| *Perameles nasuta* | B | Russulales | Albatrellaceae | *Leucogaster* | *Leucogaster* | *Leucogaster* | 1 | Vernes 2014 |
| *Perameles nasuta* | B | Hysterangiales | Mesophelliaceae | *Mesophellia* | *Mesophellia* | *Mesophellia* | 1 | Claridge 1993 |
| *Perameles nasuta* | B | Hysterangiales | Mesophelliaceae | *Mesophellia* | *Mesophellia* | *Mesophellia* | 1 | Claridge et al. 1991 |
| *Perameles nasuta* | B | Boletales | Octavianiaceae | *Octaviania* | *Octaviania tasmanica* | *Octaviania tasmanica* | 1 | Claridge 1993 |
| *Perameles nasuta* | B | Phallales | Protophallaceae | *Protubera* | *Protubera* | *Protubera* | 1 | Vernes 2014 |
| *Perameles nasuta* | B | unknown | unknown | *Pseudohysterangium* | *Pseudohysterangium* | *Pseudohysterangium* | 1 | Reddell et al. 1997 |
| *Perameles nasuta* | B | Agaricales | Entolomataceae | *Richoniella* | *Richoniella pumila* | *Entoloma gasteromycetoides* | 1 | Claridge 1993 |
| *Perameles nasuta* | B | Boletales | Sclerodermataceae | *Scleroderma* | *Scleroderma* | *Scleroderma* | 1 | Vernes 2014 |
| *Perameles nasuta* | B | Agaricales | Cortinariaceae | *Thaxterogaster* | *Thaxterogaster scabrosus* | *Cortinarius scabrosus* | 1 | Claridge 1993 |
| *Perameles nasuta* | B | Agaricales | Cortinariaceae | *Thaxterogaster* | *Thaxterogaster sp.1 Vernes, K* | *Cortinarius* | 1 | Vernes 2014 |
| *Perameles nasuta* | B | Agaricales | Cortinariaceae | *Thaxterogaster* | *Thaxterogaster sp.2 Vernes, K* | *Cortinarius* | 1 | Vernes 2014 |
| *Perameles nasuta* | B | Agaricales | Cortinariaceae | *Thaxterogaster* | *Thaxterogaster sp.3 Vernes, K* | *Cortinarius* | 1 | Vernes 2014 |
| *Perameles nasuta* | B | unknown | Gasteromycetes | *Unknown* | *Unknown* |  | 0 | Claridge 1993 |
| *Perameles nasuta* | Z | Endogonales | Endogonaceae | *Unknown* | *Unknown* |  | 0 | Claridge 1993 |
| *Perameles nasuta* | B | unknown | unknown | *Unknown* | *Unknown* |  | 0 | Reddell et al. 1997 |
| *Perameles nasuta* | B | Agaricales | Cortinariaceae | *Unknown* | *Unknown* |  | 0 | Reddell et al. 1997 |
| *Perameles nasuta* | B | Hysterangiales | Mesophelliaceae | *Unknown* | *Unknown* |  | 0 | Reddell et al. 1997 |
| *Perameles nasuta* | B | Agaricales | Russulaceae | *Unknown* | *Unknown* |  | 0 | Reddell et al. 1997 |
| *Perameles nasuta* | B | Agaricales | Coprinaceae | *Unknown* | *Unknown* |  | 0 | Vernes 2014 |
| *Perameles nasuta* | B | Hysterangiales | Mesophelliaceae | *Unknown* | *Unknown* |  | 0 | Vernes 2014 |
| *Perameles nasuta* | u | unknown | unknown | *Unknown* | *Unknown* |  | 0 | Vernes 2014 |
| *Perameles nasuta* | u | unknown | unknown | *Unknown* | *Unknown [Opaque black, spherical spore] Claridge, A W* |  | 0 | Claridge 1993 |
| *Perameles nasuta* | u | unknown | unknown | *Unknown* | *Unknown sp. B Claridge, A W* |  | 0 | Claridge et al. 1991 |
| *Perameles nasuta* | u | unknown | unknown | *Unknown* | *Unknown sp. C Claridge, A W* |  | 0 | Claridge et al. 1991 |
| *Perameles nasuta* | A | unknown | unknown | *Unknown* | *Unknown sp.1 Vernes, K* |  | 0 | Vernes 2014 |
| *Perameles nasuta* | B | Boletales | unknown | *Unknown* | *Unknown sp.1 Vernes, K* |  | 0 | Vernes 2014 |
| *Perameles nasuta* | B | Russulales | unknown | *Unknown* | *Unknown sp.1 Vernes, K* |  | 0 | Vernes 2014 |
| *Perameles nasuta* | u | unknown | unknown | *Unknown* | *Unknown sp.1 Vernes, K* |  | 0 | Vernes 2014 |
| *Perameles nasuta* | u | unknown | unknown | *Unknown* | *Unknown sp.10 Vernes, K* |  | 0 | Vernes 2014 |
| *Perameles nasuta* | A | unknown | unknown | *Unknown* | *Unknown sp.2 Vernes, K* |  | 0 | Vernes 2014 |
| *Perameles nasuta* | B | Russulales | unknown | *Unknown* | *Unknown sp.3 Vernes, K* |  | 0 | Vernes 2014 |
| *Perameles nasuta* | B | Russulales | Russulaceae | *Zelleromyces* | *Zelleromyces* | *Zelleromyces* | 1 | Claridge 1993 |
| *Perameles nasuta* | B | Russulales | Russulaceae | *Zelleromyces* | *Zelleromyces daucinus* | *Zelleromyces daucinus* | 1 | Claridge 1993 |
| *Petrogale penicillata* | B | Agaricales | Agaricaceae | *Agaricus* | *Agaricus sp.1 Vernes, K* | *Agaricus* | 1 | Vernes 2010 |
| *Petrogale penicillata* | G | Glomerales | Glomeraceae | *Glomus* | *Glomus* | *Glomus* | 1 | Vernes 2010 |
| *Petrogale penicillata* | B | Boletales | Sclerodermataceae | *Scleroderma* | *Scleroderma sp.2 Vernes, K* | *Scleroderma* | 1 | Vernes 2010 |
| *Petrogale penicillata* | B | Agaricales | Cortinariaceae | *Thaxterogaster* | *Thaxterogaster* | *Cortinarius* | 1 | Vernes 2010 |
| *Petrogale penicillata* | B | Boletales | unknown | *Unknown* | *Unknown sp.1 Vernes, K* |  | 0 | Vernes 2010 |
| *Petrogale penicillata* | u | unknown | unknown | *Unknown* | *Unknown sp.2 Vernes, K* |  | 0 | Vernes 2010 |
| *Petrogale penicillata* | B | Russulales | unknown | *Unknown* | *Unknown sp.4 Vernes, K* |  | 0 | Vernes 2010 |
| *Potorous gilbertii* | B | Agaricales | Agaricaceae | *Agaricus* | *Agaricus bisporus* | *Agaricus bisporus* | 1 | Bougher and Friend 2009 |
| *Potorous gilbertii* | B | Russulales | Russulaceae | *Arcangeliella* | *Arcangeliella daucina* | *Zelleromyces daucinus* | 1 | Bougher and Friend 2009 |
| *Potorous gilbertii* | B | Hysterangiales | Gallaceaceae | *Austrogautieria* | *Austrogautieria* | *Austrogautieria* | 1 | Bougher and Friend 2009 |
| *Potorous gilbertii* | B | Boletales | Boletaceae | *Boletus* | *Boletus* | *Boletus sens. Lat.* | 1 | Bougher and Friend 2009 |
| *Potorous gilbertii* | B | Hysterangiales | Mesophelliaceae | *Castoreum* | *Castoreum tasmanicum* | *Castoreum tasmanicum* | 1 | Nguyen et al. 2005 |
| *Potorous gilbertii* | B | Hysterangiales | Mesophelliaceae | *Chondrogaster* | *Chondrogaster* | *Chondrogaster* | 1 | Bougher and Friend 2009 |
| *Potorous gilbertii* | B | Russulales | Russulaceae | *Cystangium* | *Cystangium seminudum* | *Cystangium seminudum* | 1 | Bougher and Friend 2009 |
| *Potorous gilbertii* | B | Agaricales | Cortinariaceae | *Descomyces* | *Descomyces* | *Descomyces* | 1 | Bougher and Friend 2009 |
| *Potorous gilbertii* | B | Agaricales | Cortinariaceae | *Descomyces* | *Descomyces* | *Descomyces* | 1 | Nguyen et al. 2005 |
| *Potorous gilbertii* | A | Elaphomycetales | Elaphomycetaceae | *Elaphomyces* | *Elaphomyces* | *Elaphomyces* | 1 | Bougher and Friend 2009 |
| *Potorous gilbertii* | A | Elaphomycetales | Elaphomycetaceae | *Elaphomyces* | *Elaphomyces* | *Elaphomyces* | 1 | Nguyen et al. 2005 |
| *Potorous gilbertii* | A | Elaphomycetales | Elaphomycetaceae | *Elaphomyces* | *Elaphomyces sp.1 Bougher and Friend 2009* | *Elaphomyces* | 1 | Bougher and Friend 2009 |
| *Potorous gilbertii* | A | Elaphomycetales | Elaphomycetaceae | *Elaphomyces* | *Elaphomyces sp.2 Bougher and Friend 2009* | *Elaphomyces* | 1 | Bougher and Friend 2009 |
| *Potorous gilbertii* | A | Elaphomycetales | Elaphomycetaceae | *Elaphomyces* | *Elaphomyces sp.3 Bougher and Friend 2009* | *Elaphomyces* | 1 | Bougher and Friend 2009 |
| *Potorous gilbertii* | G | Glomerales | Glomeraceae | *Glomus* | *Glomus* | *Glomus* | 1 | Bougher and Friend 2009 |
| *Potorous gilbertii* | G | Glomerales | Glomeraceae | *Glomus* | *Glomus sp.1 Bougher and Friend 2009* | *Glomus* | 1 | Bougher and Friend 2009 |
| *Potorous gilbertii* | G | Glomerales | Glomeraceae | *Glomus* | *Glomus sp.2 Bougher and Friend 2009* | *Glomus* | 1 | Bougher and Friend 2009 |
| *Potorous gilbertii* | B | Russulales | Russulaceae | *Gymnomyces* | *Gymnomyces boranupensis* | *Gymnomyces boranupensis* | 1 | Bougher and Friend 2009 |
| *Potorous gilbertii* | B | Russulales | Russulaceae | *Gymnomyces* | *Gymnomyces sp.1 Bougher and Friend 2009* | *Gymnomyces* | 1 | Bougher and Friend 2009 |
| *Potorous gilbertii* | B | Russulales | Russulaceae | *Gymnomyces* | *Gymnomyces sp.2 Bougher and Friend 2009* | *Gymnomyces* | 1 | Bougher and Friend 2009 |
| *Potorous gilbertii* | B | Russulales | Russulaceae | *Gymnomyces* | *Gymnomyces sp.3 Bougher and Friend 2009* | *Gymnomyces* | 1 | Bougher and Friend 2009 |
| *Potorous gilbertii* | A | Pezizales | Pezizaceae | *Hydnoplicata* | *Hydnoplicata convoluta* | *Hydnoplicata convoluta* | 1 | Bougher and Friend 2009 |
| *Potorous gilbertii* | B | Hysterangiales | Hysterangiaceae | *Hysterangium* | *Hysterangium* | *Hysterangium* | 1 | Bougher and Friend 2009 |
| *Potorous gilbertii* | B | Hysterangiales | Hysterangiaceae | *Hysterangium* | *Hysterangium* | *Hysterangium* | 1 | Nguyen et al. 2005 |
| *Potorous gilbertii* | B | Hysterangiales | Hysterangiaceae | *Hysterangium* | *Hysterangium cf. Affine* | *Hysterangium* | 1 | Bougher and Friend 2009 |
| *Potorous gilbertii* | B | Hysterangiales | Hysterangiaceae | *Hysterangium* | *Hysterangium inflatum* | *Hysterangium inflatum* | 1 | Bougher and Friend 2009 |
| *Potorous gilbertii* | B | Hysterangiales | Hysterangiaceae | *Hysterangium* | *Hysterangium sp. cystidioid Bougher and Friend 2009* | *Hysterangium* | 1 | Bougher and Friend 2009 |
| *Potorous gilbertii* | B | Hysterangiales | Hysterangiaceae | *Hysterangium* | *Hysterangium sp.1 Bougher and Friend 2009* | *Hysterangium* | 1 | Bougher and Friend 2009 |
| *Potorous gilbertii* | B | Hysterangiales | Hysterangiaceae | *Hysterangium* | *Hysterangium sp.2 Bougher and Friend 2009* | *Hysterangium* | 1 | Bougher and Friend 2009 |
| *Potorous gilbertii* | B | Agaricales | Strophariaceae | *Hysterogaster* | *Hysterogaster* | *Hysterogaster* | 1 | Bougher and Friend 2009 |
| *Potorous gilbertii* | B | Hysterangiales | Mesophelliaceae | *Mesophellia* | *Mesophellia* | *Mesophellia* | 1 | Bougher and Friend 2009 |
| *Potorous gilbertii* | B | Hysterangiales | Mesophelliaceae | *Mesophellia* | *Mesophellia* | *Mesophellia* | 1 | Nguyen et al. 2005 |
| *Potorous gilbertii* | B | unknown | unknown | *Pogisperma* | *Pogisperma sp.1 Bougher and Friend 2009* | *Pogisperma* | 1 | Bougher and Friend 2009 |
| *Potorous gilbertii* | B | unknown | unknown | *Pogisperma* | *Pogisperma sp.1(.2) Bougher and Friend 2009* | *Pogisperma* | 1 | Bougher and Friend 2009 |
| *Potorous gilbertii* | B | unknown | unknown | *Pogisperma* | *Pogisperma sp.2 Bougher and Friend 2009* | *Pogisperma* | 1 | Bougher and Friend 2009 |
| *Potorous gilbertii* | B | Agaricales | Cortinariaceae | *Protoglossum* | *Protoglossum sp.1 Bougher and Friend 2009* | *Cortinarius* | 1 | Bougher and Friend 2009 |
| *Potorous gilbertii* | B | Agaricales | Cortinariaceae | *Protoglossum* | *Protoglossum sp.2 Bougher and Friend 2009* | *Cortinarius* | 1 | Bougher and Friend 2009 |
| *Potorous gilbertii* | B | Agaricales | Cortinariaceae | *Protoglossum* | *Protoglossum sp.3 Bougher and Friend 2009* | *Cortinarius* | 1 | Bougher and Friend 2009 |
| *Potorous gilbertii* | B | Agaricales | Cortinariaceae | *Protoglossum* | *Protoglossum sp.4 Bougher and Friend 2009* | *Cortinarius* | 1 | Bougher and Friend 2009 |
| *Potorous gilbertii* | B | Agaricales | Cortinariaceae | *Protoglossum* | *Protoglossum sp.5 Bougher and Friend 2009* | *Cortinarius* | 1 | Bougher and Friend 2009 |
| *Potorous gilbertii* | B | Agaricales | Cortinariaceae | *Quadrispora* | *Quadrispora tubercularis* | *Cortinarius tubercularis* | 1 | Bougher and Friend 2009 |
| *Potorous gilbertii* | A | unknown | unknown | *Unknown* | *Unknown* |  | 0 | Bougher and Friend 2009 |
| *Potorous gilbertii* | B | Hysterangiales | unknown | *Unknown* | *Unknown* |  | 0 | Bougher and Friend 2009 |
| *Potorous gilbertii* | B | Russulales | Russulaceae | *Unknown* | *Unknown* |  | 0 | Bougher and Friend 2009 |
| *Potorous gilbertii* | B | Boletales | Sclerodermataceae | *Unknown* | *Unknown* |  | 0 | Bougher and Friend 2009 |
| *Potorous gilbertii* | B | unknown | unknown | *Unknown* | *Unknown* |  | 0 | Bougher and Friend 2009 |
| *Potorous gilbertii* | B | Agaricales | Cortinariaceae | *Unknown* | *Unknown* |  | 0 | Bougher and Friend 2009 |
| *Potorous gilbertii* | u | unknown | unknown | *Unknown* | *Unknown* |  | 0 | Bougher and Friend 2009 |
| *Potorous gilbertii* | B | Agaricales | Cortinariaceae | *Unknown* | *Unknown* |  | 0 | Nguyen et al. 2005 |
| *Potorous gilbertii* | u | unknown | unknown | *Unknown* | *Unknown [small cyclindrical spore] Nguyen et al. 2005* |  | 0 | Nguyen et al. 2005 |
| *Potorous gilbertii* | u | unknown | unknown | *Unknown* | *Unknown sp.1 Bougher and Friend 2009* |  | 0 | Bougher and Friend 2009 |
| *Potorous gilbertii* | u | unknown | unknown | *Unknown* | *Unknown sp.1 Nguyen et al. 2005* |  | 0 | Nguyen et al. 2005 |
| *Potorous gilbertii* | u | unknown | unknown | *Unknown* | *Unknown sp.10 Nguyen et al. 2005* |  | 0 | Nguyen et al. 2005 |
| *Potorous gilbertii* | u | unknown | unknown | *Unknown* | *Unknown sp.11 Nguyen et al. 2005* |  | 0 | Nguyen et al. 2005 |
| *Potorous gilbertii* | u | unknown | unknown | *Unknown* | *Unknown sp.12 Nguyen et al. 2005* |  | 0 | Nguyen et al. 2005 |
| *Potorous gilbertii* | u | unknown | unknown | *Unknown* | *Unknown sp.13 Nguyen et al. 2005* |  | 0 | Nguyen et al. 2005 |
| *Potorous gilbertii* | u | unknown | unknown | *Unknown* | *Unknown sp.14 Nguyen et al. 2005* |  | 0 | Nguyen et al. 2005 |
| *Potorous gilbertii* | u | unknown | unknown | *Unknown* | *Unknown sp.15 Nguyen et al. 2005* |  | 0 | Nguyen et al. 2005 |
| *Potorous gilbertii* | u | unknown | unknown | *Unknown* | *Unknown sp.16 Nguyen et al. 2005* |  | 0 | Nguyen et al. 2005 |
| *Potorous gilbertii* | u | unknown | unknown | *Unknown* | *Unknown sp.17 Nguyen et al. 2005* |  | 0 | Nguyen et al. 2005 |
| *Potorous gilbertii* | u | unknown | unknown | *Unknown* | *Unknown sp.18 Nguyen et al. 2005* |  | 0 | Nguyen et al. 2005 |
| *Potorous gilbertii* | u | unknown | unknown | *Unknown* | *Unknown sp.19 Nguyen et al. 2005* |  | 0 | Nguyen et al. 2005 |
| *Potorous gilbertii* | u | unknown | unknown | *Unknown* | *Unknown sp.2 Bougher and Friend 2009* |  | 0 | Bougher and Friend 2009 |
| *Potorous gilbertii* | u | unknown | unknown | *Unknown* | *Unknown sp.2 Nguyen et al. 2005* |  | 0 | Nguyen et al. 2005 |
| *Potorous gilbertii* | u | unknown | unknown | *Unknown* | *Unknown sp.20 Nguyen et al. 2005* |  | 0 | Nguyen et al. 2005 |
| *Potorous gilbertii* | u | unknown | unknown | *Unknown* | *Unknown sp.21 Nguyen et al. 2005* |  | 0 | Nguyen et al. 2005 |
| *Potorous gilbertii* | u | unknown | unknown | *Unknown* | *Unknown sp.22 Nguyen et al. 2005* |  | 0 | Nguyen et al. 2005 |
| *Potorous gilbertii* | u | unknown | unknown | *Unknown* | *Unknown sp.23 Nguyen et al. 2005* |  | 0 | Nguyen et al. 2005 |
| *Potorous gilbertii* | u | unknown | unknown | *Unknown* | *Unknown sp.24 Nguyen et al. 2005* |  | 0 | Nguyen et al. 2005 |
| *Potorous gilbertii* | u | unknown | unknown | *Unknown* | *Unknown sp.25 Nguyen et al. 2005* |  | 0 | Nguyen et al. 2005 |
| *Potorous gilbertii* | u | unknown | unknown | *Unknown* | *Unknown sp.26 et al. 1997 Nguyen et al. 2005* |  | 0 | Nguyen et al. 2005 |
| *Potorous gilbertii* | u | unknown | unknown | *Unknown* | *Unknown sp.27 Nguyen et al. 2005* |  | 0 | Nguyen et al. 2005 |
| *Potorous gilbertii* | u | unknown | unknown | *Unknown* | *Unknown sp.28 Nguyen et al. 2005* |  | 0 | Nguyen et al. 2005 |
| *Potorous gilbertii* | u | unknown | unknown | *Unknown* | *Unknown sp.29 Nguyen et al. 2005* |  | 0 | Nguyen et al. 2005 |
| *Potorous gilbertii* | u | unknown | unknown | *Unknown* | *Unknown sp.3 Bougher and Friend 2009* |  | 0 | Bougher and Friend 2009 |
| *Potorous gilbertii* | u | unknown | unknown | *Unknown* | *Unknown sp.3 Nguyen et al. 2005* |  | 0 | Nguyen et al. 2005 |
| *Potorous gilbertii* | u | unknown | unknown | *Unknown* | *Unknown sp.30 Nguyen et al. 2005* |  | 0 | Nguyen et al. 2005 |
| *Potorous gilbertii* | u | unknown | unknown | *Unknown* | *Unknown sp.31 Nguyen et al. 2005* |  | 0 | Nguyen et al. 2005 |
| *Potorous gilbertii* | u | unknown | unknown | *Unknown* | *Unknown sp.32 Nguyen et al. 2005* |  | 0 | Nguyen et al. 2005 |
| *Potorous gilbertii* | u | unknown | unknown | *Unknown* | *Unknown sp.33 Nguyen et al. 2005* |  | 0 | Nguyen et al. 2005 |
| *Potorous gilbertii* | u | unknown | unknown | *Unknown* | *Unknown sp.34 Nguyen et al. 2005* |  | 0 | Nguyen et al. 2005 |
| *Potorous gilbertii* | u | unknown | unknown | *Unknown* | *Unknown sp.35 Nguyen et al. 2005* |  | 0 | Nguyen et al. 2005 |
| *Potorous gilbertii* | u | unknown | unknown | *Unknown* | *Unknown sp.36 Nguyen et al. 2005* |  | 0 | Nguyen et al. 2005 |
| *Potorous gilbertii* | u | unknown | unknown | *Unknown* | *Unknown sp.37 Nguyen et al. 2005* |  | 0 | Nguyen et al. 2005 |
| *Potorous gilbertii* | B | Russulales | Russulaceae | *Unknown* | *Unknown sp.4 Bougher and Friend 2009* |  | 0 | Bougher and Friend 2009 |
| *Potorous gilbertii* | u | unknown | unknown | *Unknown* | *Unknown sp.4 Nguyen et al. 2005* |  | 0 | Nguyen et al. 2005 |
| *Potorous gilbertii* | B | Russulales | Russulaceae | *Unknown* | *Unknown sp.5 Bougher and Friend 2009* |  | 0 | Bougher and Friend 2009 |
| *Potorous gilbertii* | u | unknown | unknown | *Unknown* | *Unknown sp.5 Nguyen et al. 2005* |  | 0 | Nguyen et al. 2005 |
| *Potorous gilbertii* | B | Russulales | Russulaceae | *Unknown* | *Unknown sp.6 Bougher and Friend 2009* |  | 0 | Bougher and Friend 2009 |
| *Potorous gilbertii* | u | unknown | unknown | *Unknown* | *Unknown sp.6 Nguyen et al. 2005* |  | 0 | Nguyen et al. 2005 |
| *Potorous gilbertii* | u | unknown | unknown | *Unknown* | *Unknown sp.7 Nguyen et al. 2005* |  | 0 | Nguyen et al. 2005 |
| *Potorous gilbertii* | u | unknown | unknown | *Unknown* | *Unknown sp.8 Nguyen et al. 2005* |  | 0 | Nguyen et al. 2005 |
| *Potorous gilbertii* | u | unknown | unknown | *Unknown* | *Unknown sp.9 Nguyen et al. 2005* |  | 0 | Nguyen et al. 2005 |
| *Potorous longipes* | G | Diversisporales | Acaulosporaceae | *Acaulospora* | *Acaulospora cf scrobiculata* | *Acaulospora* | 1 | Hill and Triggs 1985 |
| *Potorous longipes* | A | Pezizales | Pyronemataceae | *Aleuria* | *Aleuria* | *Aleuria* | 1 | T. May |
| *Potorous longipes* | A | Pezizales | Pyronemataceae | *Aleurina* | *Aleurina aurantia* | *Aleuria aurantia* | 1 | Scotts and Seebeck 1989 |
| *Potorous longipes* | A | Pezizales | Pyronemataceae | *Aleurina* | *Aleurina calospora* | *Aleurina calospora* | 1 | Scotts and Seebeck 1989 |
| *Potorous longipes* | B | Boletales | Melanogastraceae | *Alpova* | *Alpova* | *Amanita* | 1 | Hill and Triggs 1985 |
| *Potorous longipes* | B | Boletales | Melanogastraceae | *Alpova* | *Alpova lignicolor* | *Amarrendia lignicolor* | 1 | Scotts and Seebeck 1989 |
| *Potorous longipes* | A | Pezizales | Pezizaceae | *Amylascus* | *Amylascus tasmanicus* | *Amylascus tasmanicus* | 1 | Scotts and Seebeck 1989 |
| *Potorous longipes* | A | Pezizales | Pezizaceae | *Amylascus* | *Amylascus tasmanicus* | *Amylascus tasmanicus* | 1 | T. May |
| *Potorous longipes* | B | Boletales | Boletaceae | *Boletellus* | *Boletellus* | *Boletellus* | 1 | T. May |
| *Potorous longipes* | B | Boletales | Boletaceae | *Chamonixia* | *Chamonixia* | *Rossbeevera* | 1 | Hill and Triggs 1985 |
| *Potorous longipes* | B | Boletales | Boletaceae | *Chamonixia* | *Chamonixia mucosa* | *Rossbeevera mucosa* | 1 | Hill and Triggs 1985 |
| *Potorous longipes* | B | Boletales | Boletaceae | *Chamonixia* | *Chamonixia mucosa* | *Rossbeevera mucosa* | 1 | Scotts and Seebeck 1989 |
| *Potorous longipes* | B | Boletales | Boletaceae | *Chamonixia* | *Chamonixia vittatispora* | *Rossbeevera vittatispora* | 1 | Hill and Triggs 1985 |
| *Potorous longipes* | B | Boletales | Boletaceae | *Chamonixia* | *Chamonixia vittatispora* | *Rossbeevera vittatispora* | 1 | Scotts and Seebeck 1989 |
| *Potorous longipes* | B | Russulales | Russulaceae | *Cystangium* | *Cystangium* | *Cystangium* | 1 | Hill and Triggs 1985 |
| *Potorous longipes* | B | Russulales | Russulaceae | *Cystangium* | *Cystangium cf laevis* | *Cystangium* | 1 | Hill and Triggs 1985 |
| *Potorous longipes* | B | Russulales | Russulaceae | *Cystangium* | *Cystangium phymatodisporum* | *Cystangium phymatodisporum* | 1 | Scotts and Seebeck 1989 |
| *Potorous longipes* | B | Russulales | Russulaceae | *Cystangium* | *Cystangium rodwayi* | *Cystangium rodwayi* | 1 | Hill and Triggs 1985 |
| *Potorous longipes* | B | Russulales | Russulaceae | *Cystangium* | *Cystangium rodwayi* | *Cystangium rodwayi* | 1 | Scotts and Seebeck 1989 |
| *Potorous longipes* | Z | Endogonales | Endogonaceae | *Endogone* | *Endogone* | *Endogone* | 1 | Hill and Triggs 1985 |
| *Potorous longipes* | Z | Endogonales | Endogonaceae | *Endogone* | *Endogone [double spore wall] Scotts and Seebeck 1989* | *Endogone* | 1 | Scotts and Seebeck 1989 |
| *Potorous longipes* | Z | Endogonales | Endogonaceae | *Endogone* | *Endogone [simple spore wall] Scotts and Seebeck 1989* | *Endogone* | 1 | Scotts and Seebeck 1989 |
| *Potorous longipes* | B | Gomphales | Gomphaceae | *Gautieria* | *Gautieria* | *Gautieria* | 1 | T. May |
| *Potorous longipes* | B | Gomphales | Gomphaceae | *Gautieria* | *Gautieria albida* | *Gautieria albida* | 1 | Scotts and Seebeck 1989 |
| *Potorous longipes* | B | Gomphales | Gomphaceae | *Gautieria* | *Gautieria costata* | *Austrogautieria costata* | 1 | Hill and Triggs 1985 |
| *Potorous longipes* | B | Gomphales | Gomphaceae | *Gautieria* | *Gautieria costata* | *Austrogautieria costata* | 1 | Scotts and Seebeck 1989 |
| *Potorous longipes* | A | Geoglossales | Geoglossaceae | *Geoglossum* | *Geoglossum [spores 15-septate] Scotts and Seebeck 1989* | *Geoglossum sens. Lat.* | 1 | Scotts and Seebeck 1989 |
| *Potorous longipes* | A | Geoglossales | Geoglossaceae | *Geoglossum* | *Geoglossum [spores 7-septate] Scotts and Seebeck 1989* | *Geoglossum sens. Lat.* | 1 | Scotts and Seebeck 1989 |
| *Potorous longipes* | B | Russulales | Russulaceae | *Gymnomyces* | *Gymnomyces pallidus* | *Gymnomyces pallidus* | 1 | Scotts and Seebeck 1989 |
| *Potorous longipes* | B | Russulales | Russulaceae | *Gymnomyces* | *Gymnomyces seminudus* | *Gymnomyces seminudus* | 1 | Hill and Triggs 1985 |
| *Potorous longipes* | B | Agaricales | Hydnangiaceae | *Hydnangium* | *Hydnangium archeri* | *Hydnangium archeri* | 1 | Scotts and Seebeck 1989 |
| *Potorous longipes* | B | Agaricales | Strophariaceae | *Hymenogaster* | *Hymenogaster* | *Hymenogaster* | 1 | Hill and Triggs 1985 |
| *Potorous longipes* | B | Agaricales | Strophariaceae | *Hymenogaster* | *Hymenogaster* | *Hymenogaster* | 1 | T. May |
| *Potorous longipes* | B | Agaricales | Strophariaceae | *Hymenogaster* | *Hymenogaster albus* | *Descomyces albus* | 1 | Scotts and Seebeck 1989 |
| *Potorous longipes* | B | Agaricales | Strophariaceae | *Hymenogaster* | *Hymenogaster aureus* | *Hymenogaster aureus* | 1 | Scotts and Seebeck 1989 |
| *Potorous longipes* | B | Agaricales | Strophariaceae | *Hymenogaster* | *Hymenogaster oblongisporus* | *Cortinarius oblongisporus* | 1 | Hill and Triggs 1985 |
| *Potorous longipes* | B | Agaricales | Strophariaceae | *Hymenogaster* | *Hymenogaster oblongisporus* | *Cortinarius oblongisporus* | 1 | Scotts and Seebeck 1989 |
| *Potorous longipes* | B | Agaricales | Strophariaceae | *Hymenogaster* | *Hymenogaster oblongisporus* | *Cortinarius oblongisporus* | 1 | T. May |
| *Potorous longipes* | B | Agaricales | Strophariaceae | *Hymenogaster* | *Hymenogaster violaceus* | *Cortinarius subviolaceus* | 1 | Scotts and Seebeck 1989 |
| *Potorous longipes* | B | Hysterangiales | Hysterangiaceae | *Hysterangium* | *Hysterangium* | *Hysterangium* | 1 | Hill and Triggs 1985 |
| *Potorous longipes* | B | Hysterangiales | Hysterangiaceae | *Hysterangium* | *Hysterangium affine* | *Hysterangium affine* | 1 | Scotts and Seebeck 1989 |
| *Potorous longipes* | B | Hysterangiales | Hysterangiaceae | *Hysterangium* | *Hysterangium aggregatum* | *Hysterangium aggregatum* | 1 | Scotts and Seebeck 1989 |
| *Potorous longipes* | B | Hysterangiales | Hysterangiaceae | *Hysterangium* | *Hysterangium inflatum* | *Hysterangium inflatum* | 1 | Scotts and Seebeck 1989 |
| *Potorous longipes* | B | Hysterangiales | Hysterangiaceae | *Hysterangium* | *Hysterangium inflatum* | *Hysterangium inflatum* | 1 | T. May |
| *Potorous longipes* | A | Pezizales | Pyronemataceae | *Jafneadelphus* | *Jafneadelphus* | *Jafneadelphus* | 1 | T. May |
| *Potorous longipes* | A | Pezizales | Tuberaceae | *Labyrinthomyces* | *Labyrinthomyces tessellatus* | *Dingleya tessellata* | 1 | Hill and Triggs 1985 |
| *Potorous longipes* | A | Pezizales | Tuberaceae | *Labyrinthomyces* | *Labyrinthomyces varius* | *Labyrinthomyces varius* | 1 | Hill and Triggs 1985 |
| *Potorous longipes* | A | Pezizales | Tuberaceae | *Labyrinthomyces* | *Labyrinthomyces varius* | *Labyrinthomyces varius* | 1 | Scotts and Seebeck 1989 |
| *Potorous longipes* | A | Pezizales | Tuberaceae | *Labyrinthomyces* | *Labyrinthomyces* | *Labyrinthomyces* | 1 | T. May |
| *Potorous longipes* | A | Pezizales | Pyronemataceae | *Lamprospora* | *Lamprospora* | *Lamprospora* | 1 | Scotts and Seebeck 1989 |
| *Potorous longipes* | A | Pezizales | Pyronemataceae | *Lamprospora* | *Lamprospora crec'hqueraultii* | *Lamprospora crechqueraultii* | 1 | Scotts and Seebeck 1989 |
| *Potorous longipes* | B | Russulales | Albatrellaceae | *Leucogaster* | *Leucogaster* | *Leucogaster* | 1 | Hill and Triggs 1985 |
| *Potorous longipes* | B | Russulales | Russulaceae | *Martellia* | *Martellia redolens* | *Gymnomyces redolens* | 1 | Scotts and Seebeck 1989 |
| *Potorous longipes* | B | Hysterangiales | Mesophelliaceae | *Mesophellia* | *Mesophellia* | *Mesophellia* | 1 | Scotts and Seebeck 1989 |
| *Potorous longipes* | B | Hysterangiales | Mesophelliaceae | *Mesophellia* | *Mesophellia* | *Mesophellia* | 1 | T. May |
| *Potorous longipes* | B | Hysterangiales | Mesophelliaceae | *Mesophellia* | *Mesophellia [spore class 49] Green et al 1999* | *Mesophellia* | 1 | Green et al. 1999 |
| *Potorous longipes* | A | Hypocreales | Ceratostomataceae | *Microthecium* | *Microthecium beatonii* | *Sphaerodes beatonii* | 1 | Hill and Triggs 1985 |
| *Potorous longipes* | B | Boletales | Octavianiaceae | *Octaviania* | *Octaviania tasmanica* | *Octaviania tasmanica* | 1 | Hill and Triggs 1985 |
| *Potorous longipes* | B | Boletales | Octavianiaceae | *Octaviania* | *Octaviania tasmanica* | *Octaviania tasmanica* | 1 | Scotts and Seebeck 1989 |
| *Potorous longipes* | B | Boletales | Octavianiaceae | *Octaviania* | *Octaviania tasmanica* | *Octaviania tasmanica* | 1 | T. May |
| *Potorous longipes* | A | Pezizales | Pezizaceae | *Peziza* | *Peziza whitei* | *Hydnoplicata convoluta* | 1 | T. May |
| *Potorous longipes* | B | Agaricales | Hydnangiaceae | *Podohydnangium* | *Podohydnangium* | *Podohydnangium* | 1 | Scotts and Seebeck 1989 |
| *Potorous longipes* | B | Agaricales | Entolomataceae | *Richoniella* | *Richoniella* | *Richoniella* | 1 | Hill and Triggs 1985 |
| *Potorous longipes* | B | Boletales | Sclerodermataceae | *Scleroderma* | *Scleroderma* | *Scleroderma* | 1 | Scotts and Seebeck 1989 |
| *Potorous longipes* | B | Boletales | Sclerodermataceae | *Scleroderma* | *Scleroderma paradoxum* | *Scleroderma paradoxum* | 1 | Scotts and Seebeck 1989 |
| *Potorous longipes* | B | Agaricales | Stephanosporaceae | *Stephanospora* | *Stephanospora flava* | *Stephanospora flava* | 1 | Scotts and Seebeck 1989 |
| *Potorous longipes* | B | Agaricales | Stephanosporaceae | *Stephanospora* | *Stephanospora flava* | *Stephanospora flava* | 1 | T. May |
| *Potorous longipes* | B | Agaricales | Cortinariaceae | *Thaxterogaster* | *Thaxterogaster* | *Cortinarius* | 1 | Hill and Triggs 1985 |
| *Potorous longipes* | B | Agaricales | Cortinariaceae | *Thaxterogaster* | *Thaxterogaster piriformis* | *Cortinarius piriforme* | 1 | Scotts and Seebeck 1989 |
| *Potorous longipes* | B | Agaricales | Entolomataceae | *Unknown* | *Unknown* |  | 0 | Scotts and Seebeck 1989 |
| *Potorous longipes* | B | Boletales | Boletaceae | *Unknown* | *Unknown* |  | 0 | T. May |
| *Potorous longipes* | Z | Endogonales | Endogonaceae | *Unknown* | *Unknown* |  | 0 | T. May |
| *Potorous longipes* | B | unknown | unknown | *Unknown* | *Unknown [spore class 12] Green et al 1999* |  | 0 | Green et al. 1999 |
| *Potorous longipes* | B | unknown | unknown | *Unknown* | *Unknown [spore class 27] Green et al 1999* |  | 0 | Green et al. 1999 |
| *Potorous longipes* | B | unknown | unknown | *Unknown* | *Unknown [spore class 28] Green et al 1999* |  | 0 | Green et al. 1999 |
| *Potorous longipes* | B | unknown | unknown | *Unknown* | *Unknown [spore class 29] Green et al 1999* |  | 0 | Green et al. 1999 |
| *Potorous longipes* | B | unknown | unknown | *Unknown* | *Unknown [spore class 31] Green et al 1999* |  | 0 | Green et al. 1999 |
| *Potorous longipes* | B | unknown | unknown | *Unknown* | *Unknown [spore class 45] Green et al 1999* |  | 0 | Green et al. 1999 |
| *Potorous longipes* | B | unknown | unknown | *Unknown* | *Unknown [spore class 47] Green et al 1999* | *Hysterangium* | 1 | Green et al. 1999 |
| *Potorous longipes* | B | unknown | unknown | *Unknown* | *Unknown [spore class 48] Green et al 1999* |  | 0 | Green et al. 1999 |
| *Potorous longipes* | B | unknown | unknown | *Unknown* | *Unknown [spore class 54] Green et al 1999* |  | 0 | Green et al. 1999 |
| *Potorous longipes* | B | unknown | unknown | *Unknown* | *Unknown [spore class 60] Green et al 1999* |  | 0 | Green et al. 1999 |
| *Potorous longipes* | B | unknown | unknown | *Unknown* | *Unknown radicatum [spore class 46] Green et al 1999* |  | 0 | Green et al. 1999 |
| *Potorous longipes* | B | Russulales | Russulaceae | *Zelleromyces* | *Zelleromyces* | *Zelleromyces* | 1 | Hill and Triggs 1985 |
| *Potorous longipes* | B | Russulales | Russulaceae | *Zelleromyces* | *Zelleromyces* | *Zelleromyces* | 1 | Scotts and Seebeck 1989 |
| *Potorous longipes* | B | Russulales | Russulaceae | *Zelleromyces* | *Zelleromyces striatus* | *Zelleromyces striatus* | 1 | Hill and Triggs 1985 |
| *Potorous tridactylus* | B | Boletales | Melanogastraceae | *Alpova* | *Alpova clelandii* |  | 0 | Bennett and Baxter 1989 |
| *Potorous tridactylus* | B | Boletales | Melanogastraceae | *Alpova* | *Alpova grandisporus* | *Amanita grandispora* | 1 | Bennett and Baxter 1989 |
| *Potorous tridactylus* | B | Boletales | Melanogastraceae | *Alpova* | *Alpova grandisporus* | *Amanita grandispora* | 1 | Claridge et al. 1992 |
| *Potorous tridactylus* | B | Boletales | Melanogastraceae | *Alpova* | *Alpova grandisporus* | *Amanita grandispora* | 1 | Claridge et al. 1993 |
| *Potorous tridactylus* | B | Boletales | Melanogastraceae | *Alpova* | *Alpova lignicolor* | *Amarrendia lignicolor* | 1 | Bennett and Baxter 1989 |
| *Potorous tridactylus* | A | Pezizales | Pezizaceae | *Amylascus* | *Amylascus tasmanicus* | *Amylascus tasmanicus* | 1 | Claridge et al. 1993 |
| *Potorous tridactylus* | B | Agaricales | Cortinariaceae | *Aroramyces* | *Aroramyces sp.1 Vernes, K* | *Aroramyces* | 1 | Vernes 2010 |
| *Potorous tridactylus* | B | Hysterangiales | Mesophelliaceae | *Castoreum* | *Castoreum* | *Castoreum* | 1 | Claridge et al. 1992 |
| *Potorous tridactylus* | B | Hysterangiales | Mesophelliaceae | *Castoreum* | *Castoreum tasmanicum* | *Castoreum tasmanicum* | 1 | Claridge et al. 1993 |
| *Potorous tridactylus* | B | Boletales | Boletaceae | *Chamonixia* | *Chamonixia* | *Rossbeevera* | 1 | Bennett and Baxter 1989 |
| *Potorous tridactylus* | B | Boletales | Boletaceae | *Chamonixia* | *Chamonixia* | *Rossbeevera* | 1 | Claridge et al. 1992 |
| *Potorous tridactylus* | B | Boletales | Boletaceae | *Chamonixia* | *Chamonixia* | *Rossbeevera* | 1 | Claridge et al. 1993 |
| *Potorous tridactylus* | B | Boletales | Boletaceae | *Chamonixia* | *Chamonixia* | *Rossbeevera* | 1 | Tory et al. 1997 |
| *Potorous tridactylus* | B | Boletales | Boletaceae | *Chamonixia* | *Chamonixia mucosa* | *Rossbeevera mucosa* | 1 | Bennett and Baxter 1989 |
| *Potorous tridactylus* | B | Boletales | Boletaceae | *Chamonixia* | *Chamonixia pachydermis* | *Rossbeevera pachydermis* | 1 | Claridge et al. 1993 |
| *Potorous tridactylus* | B | Boletales | Boletaceae | *Chamonixia* | *Chamonixia vittatispora* | *Rossbeevera vittatispora* | 1 | Bennett and Baxter 1989 |
| *Potorous tridactylus* | B | Boletales | Boletaceae | *Chamonixia* | *Chamonixia vittatispora* | *Rossbeevera vittatispora* | 1 | Claridge et al. 1993 |
| *Potorous tridactylus* | B | Russulales | Russulaceae | *Cystangium* | *Cystangium phymatodisporum* | *Cystangium phymatodisporum* | 1 | Bennett and Baxter 1989 |
| *Potorous tridactylus* | B | Russulales | Russulaceae | *Cystangium* | *Cystangium rodwayi* | *Cystangium rodwayi* | 1 | Bennett and Baxter 1989 |
| *Potorous tridactylus* | B | Agaricales | Cortinariaceae | *Descomyces* | *Descomyces zeylanicus* | *Descomyces zeylanicus* | 1 | Tory et al. 1997 |
| *Potorous tridactylus* | A | Pezizales | Tuberaceae | *Dingleya* | *Dingleya* | *Dingleya* | 1 | Vernes 2010 |
| *Potorous tridactylus* | A | Elaphomycetales | Elaphomycetaceae | *Elaphomyces* | *Elaphomyces* | *Elaphomyces* | 1 | Vernes 2010 |
| *Potorous tridactylus* | Z | Endogonales | Endogonaceae | *Endogone* | *Endogone* | *Endogone* | 1 | Claridge et al. 1992 |
| *Potorous tridactylus* | Z | Endogonales | Endogonaceae | *Endogone* | *Endogone [spore walls double-layered] Claridge, A W* | *Endogone* | 1 | Claridge et al. 1993 |
| *Potorous tridactylus* | Z | Endogonales | Endogonaceae | *Endogone* | *Endogone [spore walls single-layered] Claridge, A W* | *Endogone* | 1 | Claridge et al. 1993 |
| *Potorous tridactylus* | Z | Endogonales | Endogonaceae | *Endogone* | *Endogone sp. [spore walls complex] Bennett and Baxter 1989* | *Endogone* | 1 | Bennett and Baxter 1989 |
| *Potorous tridactylus* | Z | Endogonales | Endogonaceae | *Endogone* | *Endogone sp. [spore walls simple] Bennett and Baxter 1989* | *Endogone* | 1 | Bennett and Baxter 1989 |
| *Potorous tridactylus* | B | Gomphales | Gomphaceae | *Gautieria* | *Gautieria* | *Gautieria* | 1 | Bennett and Baxter 1989 |
| *Potorous tridactylus* | B | Gomphales | Gomphaceae | *Gautieria* | *Gautieria albida* | *Gautieria albida* | 1 | Claridge et al. 1993 |
| *Potorous tridactylus* | B | Gomphales | Gomphaceae | *Gautieria* | *Gautieria costata* | *Austrogautieria costata* | 1 | Bennett and Baxter 1989 |
| *Potorous tridactylus* | B | Gomphales | Gomphaceae | *Gautieria* | *Gautieria costata* | *Austrogautieria costata* | 1 | Claridge et al. 1992 |
| *Potorous tridactylus* | B | Gomphales | Gomphaceae | *Gautieria* | *Gautieria monospora* | *Gautieria monospora* | 1 | Claridge et al. 1992 |
| *Potorous tridactylus* | B | Gomphales | Gomphaceae | *Gautieria* | *Gautieria monospora* | *Gautieria monospora* | 1 | Claridge et al. 1993 |
| *Potorous tridactylus* | B | Gomphales | Gomphaceae | *Gautieria* | *Gautieria monospora* | *Gautieria monospora* | 1 | Tory et al. 1997 |
| *Potorous tridactylus* | B | Gomphales | Gomphaceae | *Gautieria* | *Gautieria sp.1 Claridge, A W* | *Gautieria* | 1 | Claridge et al. 1993 |
| *Potorous tridactylus* | B | Gomphales | Gomphaceae | *Gautieria* | *Gautieria sp.2 Claridge, A W* | *Gautieria* | 1 | Claridge et al. 1993 |
| *Potorous tridactylus* | A | Geoglossales | Geoglossaceae | *Geoglossum* | *Geoglossum* | *Geoglossum sens. Lat.* | 1 | Bennett and Baxter 1989 |
| *Potorous tridactylus* | B | Russulales | Russulaceae | *Gymnomyces* | *Gymnomyces eildonensis* | *Gymnomyces eildonensis* | 1 | Bennett and Baxter 1989 |
| *Potorous tridactylus* | B | Russulales | Russulaceae | *Gymnomyces* | *Gymnomyces pallidus* | *Gymnomyces pallidus* | 1 | Bennett and Baxter 1989 |
| *Potorous tridactylus* | B | Russulales | Russulaceae | *Gymnomyces* | *Gymnomyces seminudus* | *Gymnomyces seminudus* | 1 | Bennett and Baxter 1989 |
| *Potorous tridactylus* | B | Agaricales | Hydnangiaceae | *Hydnangium* | *Hydnangium archeri* | *Hydnangium archeri* | 1 | Bennett and Baxter 1989 |
| *Potorous tridactylus* | B | Agaricales | Hydnangiaceae | *Hydnangium* | *Hydnangium archeri* | *Hydnangium archeri* | 1 | Claridge et al. 1992 |
| *Potorous tridactylus* | B | Agaricales | Hydnangiaceae | *Hydnangium* | *Hydnangium carneum* | *Hydnangium carneum* | 1 | Bennett and Baxter 1989 |
| *Potorous tridactylus* | B | Agaricales | Strophariaceae | *Hymenogaster* | *Hymenogaster* | *Hymenogaster* | 1 | Bennett and Baxter 1989 |
| *Potorous tridactylus* | B | Agaricales | Strophariaceae | *Hymenogaster* | *Hymenogaster albus* | *Descomyces albus* | 1 | Bennett and Baxter 1989 |
| *Potorous tridactylus* | B | Agaricales | Strophariaceae | *Hymenogaster* | *Hymenogaster albus* | *Descomyces albus* | 1 | Claridge et al. 1992 |
| *Potorous tridactylus* | B | Agaricales | Strophariaceae | *Hymenogaster* | *Hymenogaster albus* | *Descomyces albus* | 1 | Claridge et al. 1993 |
| *Potorous tridactylus* | B | Agaricales | Strophariaceae | *Hymenogaster* | *Hymenogaster atratus* | *Cortinarius atratus* | 1 | Bennett and Baxter 1989 |
| *Potorous tridactylus* | B | Agaricales | Strophariaceae | *Hymenogaster* | *Hymenogaster atratus* | *Cortinarius atratus* | 1 | Claridge et al. 1992 |
| *Potorous tridactylus* | B | Agaricales | Strophariaceae | *Hymenogaster* | *Hymenogaster atratus* | *Cortinarius atratus* | 1 | Claridge et al. 1993 |
| *Potorous tridactylus* | B | Agaricales | Strophariaceae | *Hymenogaster* | *Hymenogaster aureus* | *Hymenogaster aureus* | 1 | Claridge et al. 1992 |
| *Potorous tridactylus* | B | Agaricales | Strophariaceae | *Hymenogaster* | *Hymenogaster fusisporus* | *Hysterogaster fusisporus* | 1 | Claridge et al. 1992 |
| *Potorous tridactylus* | B | Agaricales | Strophariaceae | *Hymenogaster* | *Hymenogaster macrosporus* | *Timgrovea macrospora* | 1 | Claridge et al. 1993 |
| *Potorous tridactylus* | B | Agaricales | Strophariaceae | *Hymenogaster* | *Hymenogaster nanus* | *Hymenogaster nanus* | 1 | Claridge et al. 1993 |
| *Potorous tridactylus* | B | Agaricales | Strophariaceae | *Hymenogaster* | *Hymenogaster oblongisporus* | *Cortinarius oblongisporus* | 1 | Bennett and Baxter 1989 |
| *Potorous tridactylus* | B | Agaricales | Strophariaceae | *Hymenogaster* | *Hymenogaster oblongisporus* | *Cortinarius oblongisporus* | 1 | Claridge et al. 1993 |
| *Potorous tridactylus* | B | Agaricales | Strophariaceae | *Hymenogaster* | *Hymenogaster reticulatus* | *Timgrovea reticulata* | 1 | Claridge et al. 1993 |
| *Potorous tridactylus* | B | Agaricales | Strophariaceae | *Hymenogaster* | *Hymenogaster violaceus* | *Cortinarius subviolaceus* | 1 | Bennett and Baxter 1989 |
| *Potorous tridactylus* | B | Agaricales | Strophariaceae | *Hymenogaster* | *Hymenogaster viscidus* | *Cortinarius oleosus* | 1 | Bennett and Baxter 1989 |
| *Potorous tridactylus* | B | Agaricales | Strophariaceae | *Hymenogaster* | *Hymenogaster viscidus* | *Cortinarius oleosus* | 1 | Claridge et al. 1993 |
| *Potorous tridactylus* | B | Agaricales | Strophariaceae | *Hymenogaster* | *Hymenogaster zeylanicus* | *Descomyces albellus* | 1 | Bennett and Baxter 1989 |
| *Potorous tridactylus* | B | Agaricales | Strophariaceae | *Hymenogaster* | *Hymenogaster zeylanicus* | *Descomyces albellus* | 1 | Claridge et al. 1992 |
| *Potorous tridactylus* | B | Agaricales | Strophariaceae | *Hymenogaster* | *Hymenogaster zeylanicus* | *Descomyces albellus* | 1 | Claridge et al. 1993 |
| *Potorous tridactylus* | B | Hysterangiales | Hysterangiaceae | *Hysterangium* | *Hysterangium* | *Hysterangium* | 1 | Tory et al. 1997 |
| *Potorous tridactylus* | B | Hysterangiales | Hysterangiaceae | *Hysterangium* | *Hysterangium* | *Hysterangium* | 1 | Vernes 2010 |
| *Potorous tridactylus* | B | Hysterangiales | Hysterangiaceae | *Hysterangium* | *Hysterangium affine* | *Hysterangium affine* | 1 | Bennett and Baxter 1989 |
| *Potorous tridactylus* | B | Hysterangiales | Hysterangiaceae | *Hysterangium* | *Hysterangium aggregatum* | *Hysterangium aggregatum* | 1 | Bennett and Baxter 1989 |
| *Potorous tridactylus* | B | Hysterangiales | Hysterangiaceae | *Hysterangium* | *Hysterangium aggregatum* | *Hysterangium aggregatum* | 1 | Claridge et al. 1992 |
| *Potorous tridactylus* | B | Hysterangiales | Hysterangiaceae | *Hysterangium* | *Hysterangium aggregatum* | *Hysterangium aggregatum* | 1 | Claridge et al. 1993 |
| *Potorous tridactylus* | B | Hysterangiales | Hysterangiaceae | *Hysterangium* | *Hysterangium gelatinosporum* | *Aroramyces gelatinosporus* | 1 | Claridge et al. 1992 |
| *Potorous tridactylus* | B | Hysterangiales | Hysterangiaceae | *Hysterangium* | *Hysterangium gelatinosporum* | *Aroramyces gelatinosporus* | 1 | Claridge et al. 1993 |
| *Potorous tridactylus* | B | Hysterangiales | Hysterangiaceae | *Hysterangium* | *Hysterangium inflatum* | *Hysterangium inflatum* | 1 | Bennett and Baxter 1989 |
| *Potorous tridactylus* | B | Hysterangiales | Hysterangiaceae | *Hysterangium* | *Hysterangium inflatum* | *Hysterangium inflatum* | 1 | Claridge et al. 1992 |
| *Potorous tridactylus* | B | Hysterangiales | Hysterangiaceae | *Hysterangium* | *Hysterangium inflatum* | *Hysterangium inflatum* | 1 | Claridge et al. 1993 |
| *Potorous tridactylus* | B | Hysterangiales | Hysterangiaceae | *Hysterangium* | *Hysterangium salmonaceum* | *Hysterangium salmonaceum* | 1 | Claridge et al. 1992 |
| *Potorous tridactylus* | B | Hysterangiales | Hysterangiaceae | *Hysterangium* | *Hysterangium sp.1 Claridge, A W* | *Hysterangium* | 1 | Claridge et al. 1993 |
| *Potorous tridactylus* | B | Hysterangiales | Hysterangiaceae | *Hysterangium* | *Hysterangium sp.2 Claridge, A W* | *Hysterangium* | 1 | Claridge et al. 1993 |
| *Potorous tridactylus* | A | Pezizales | Pyronemataceae | *Jafneadelphus* | *Jafneadelphus* | *Jafneadelphus* | 1 | Claridge et al. 1992 |
| *Potorous tridactylus* | A | Pezizales | Pyronemataceae | *Jafneadelphus* | *Jafneadelphus* | *Jafneadelphus* | 1 | Claridge et al. 1993 |
| *Potorous tridactylus* | A | Pezizales | Pyronemataceae | *Jafneadelphus* | *Jafneadelphus calosporus* | *Aleurina calospora* | 1 | Bennett and Baxter 1989 |
| *Potorous tridactylus* | A | Pezizales | Tuberaceae | *Labyrinthomyces* | *Labyrinthomyces sp.1 Vernes, K* | *Labyrinthomyces* | 1 | Vernes 2010 |
| *Potorous tridactylus* | A | Pezizales | Tuberaceae | *Labyrinthomyces* | *Labyrinthomyces tessellatus* | *Dingleya tessellata* | 1 | Claridge et al. 1992 |
| *Potorous tridactylus* | A | Pezizales | Tuberaceae | *Labyrinthomyces* | *Labyrinthomyces varius* | *Labyrinthomyces varius* | 1 | Bennett and Baxter 1989 |
| *Potorous tridactylus* | A | Pezizales | Tuberaceae | *Labyrinthomyces* | *Labyrinthomyces varius* | *Labyrinthomyces varius* | 1 | Claridge et al. 1992 |
| *Potorous tridactylus* | A | Pezizales | Tuberaceae | *Labyrinthomyces* | *Labyrinthomyces varius* | *Labyrinthomyces varius* | 1 | Claridge et al. 1993 |
| *Potorous tridactylus* | B | Russulales | Albatrellaceae | *Leucogaster* | *Leucogaster meridionales* | *Leucogaster meridionalis* | 1 | Claridge et al. 1993 |
| *Potorous tridactylus* | B | Russulales | Albatrellaceae | *Leucogaster* | *Leucogaster meridionalis* | *Leucogaster meridionalis* | 1 | Vernes 2010 |
| *Potorous tridactylus* | B | Russulales | Russulaceae | *Martellia* | *Martellia* |  | 0 | Bennett and Baxter 1989 |
| *Potorous tridactylus* | B | Russulales | Russulaceae | *Martellia* | *Martellia redolens* | *Gymnomyces redolens* | 1 | Claridge et al. 1992 |
| *Potorous tridactylus* | B | Hysterangiales | Mesophelliaceae | *Mesophellia* | *Mesophellia* | *Mesophellia* | 1 | Bennett and Baxter 1989 |
| *Potorous tridactylus* | B | Hysterangiales | Mesophelliaceae | *Mesophellia* | *Mesophellia* | *Mesophellia* | 1 | Claridge et al. 1993 |
| *Potorous tridactylus* | B | Hysterangiales | Mesophelliaceae | *Mesophellia* | *Mesophellia pachythrix* | *Andebbia pachythrix* | 1 | Claridge et al. 1992 |
| *Potorous tridactylus* | A | Hypocreales | Ceratostomataceae | *Microthecium* | *Microthecium beatonii* | *Sphaerodes beatonii* | 1 | Bennett and Baxter 1989 |
| *Potorous tridactylus* | B | Boletales | Octavianiaceae | *Octaviania* | *Octaviania tasmanica* | *Octaviania tasmanica* | 1 | Bennett and Baxter 1989 |
| *Potorous tridactylus* | B | Boletales | Octavianiaceae | *Octaviania* | *Octaviania tasmanica* | *Octaviania tasmanica* | 1 | Claridge et al. 1992 |
| *Potorous tridactylus* | B | Boletales | Octavianiaceae | *Octaviania* | *Octaviania tasmanica* | *Octaviania tasmanica* | 1 | Claridge et al. 1993 |
| *Potorous tridactylus* | A | Pezizales | Pezizaceae | *Peziza* | *Peziza whitei* | *Hydnoplicata convoluta* | 1 | Bennett and Baxter 1989 |
| *Potorous tridactylus* | A | Pezizales | Pezizaceae | *Peziza* | *Peziza whitei* | *Hydnoplicata convoluta* | 1 | Claridge et al. 1993 |
| *Potorous tridactylus* | A | Pezizales | Pezizaceae | *Peziza* | *Peziza whitei* | *Hydnoplicata convoluta* | 1 | Tory et al. 1997 |
| *Potorous tridactylus* | A | Pezizales | Pyronemataceae | *Pulvinula* | *Pulvinula archeri* | *Pulvinula archeri* | 1 | Bennett and Baxter 1989 |
| *Potorous tridactylus* | B | Agaricales | Entolomataceae | *Richoniella* | *Richoniella* | *Richoniella* | 1 | Bennett and Baxter 1989 |
| *Potorous tridactylus* | B | Agaricales | Entolomataceae | *Richoniella* | *Richoniella pumila* | *Entoloma gasteromycetoides* | 1 | Claridge et al. 1993 |
| *Potorous tridactylus* | B | Boletales | Sclerodermataceae | *Scleroderma* | *Scleroderma paradoxum* | *Scleroderma paradoxum* | 1 | Claridge et al. 1993 |
| *Potorous tridactylus* | A | Pezizales | Pezizaceae | *Sphaerozone* | *Sphaerozone echinulatum* | *Gymnohydnotrya echinulata* | 1 | Claridge et al. 1993 |
| *Potorous tridactylus* | B | Agaricales | Stephanosporaceae | *Stephanospora* | *Stephanospora* | *Stephanospora* | 1 | Bennett and Baxter 1989 |
| *Potorous tridactylus* | B | Agaricales | Stephanosporaceae | *Stephanospora* | *Stephanospora flava* | *Stephanospora flava* | 1 | Claridge et al. 1993 |
| *Potorous tridactylus* | B | Agaricales | Cortinariaceae | *Thaxterogaster* | *Thaxterogaster* | *Cortinarius* | 1 | Vernes 2010 |
| *Potorous tridactylus* | B | Agaricales | Cortinariaceae | *Thaxterogaster* | *Thaxterogaster campbellae* | *Cortinarius campbelliae* | 1 | Bennett and Baxter 1989 |
| *Potorous tridactylus* | B | Agaricales | Cortinariaceae | *Thaxterogaster* | *Thaxterogaster leucocephalus* | *Cortinarius leucocephalus* | 1 | Claridge et al. 1993 |
| *Potorous tridactylus* | B | Agaricales | Cortinariaceae | *Thaxterogaster* | *Thaxterogaster levisporus* | *Cortinarius levisporus* | 1 | Claridge et al. 1992 |
| *Potorous tridactylus* | B | Agaricales | Cortinariaceae | *Thaxterogaster* | *Thaxterogaster piriformis* | *Cortinarius piriforme* | 1 | Bennett and Baxter 1989 |
| *Potorous tridactylus* | B | Agaricales | Cortinariaceae | *Thaxterogaster* | *Thaxterogaster scabrosus* | *Cortinarius scabrosus* | 1 | Claridge et al. 1992 |
| *Potorous tridactylus* | B | Agaricales | Cortinariaceae | *Thaxterogaster* | *Thaxterogaster scabrosus* | *Cortinarius scabrosus* | 1 | Claridge et al. 1993 |
| *Potorous tridactylus* | B | Agaricales | Cortinariaceae | *Thaxterogaster* | *Thaxterogaster sp.1 Claridge, A W* | *Cortinarius* | 1 | Claridge et al. 1993 |
| *Potorous tridactylus* | B | Agaricales | Cortinariaceae | *Thaxterogaster* | *Thaxterogaster sp.2 Claridge, A W* | *Cortinarius* | 1 | Claridge et al. 1993 |
| *Potorous tridactylus* | B | Agaricales | Cortinariaceae | *Thaxterogaster* | *Thaxterogaster sp.3 Claridge, A W* | *Cortinarius* | 1 | Claridge et al. 1993 |
| *Potorous tridactylus* | B | Agaricales | Cortinariaceae | *Thaxterogaster* | *Thaxterogaster sp.4 Claridge, A W* | *Cortinarius* | 1 | Claridge et al. 1993 |
| *Potorous tridactylus* | A | Pezizales | Pyronemataceae | *Unknown* | *Unknown* |  | 0 | Bennett and Baxter 1989 |
| *Potorous tridactylus* | B | Russulales | Russulaceae | *Unknown* | *Unknown* |  | 0 | Claridge et al. 1993 |
| *Potorous tridactylus* | B | Agaricales | Hydnangiaceae | *Unknown* | *Unknown* |  | 0 | Claridge et al. 1993 |
| *Potorous tridactylus* | u | unknown | unknown | *Unknown* | *Unknown* |  | 0 | Tory et al. 1997 |
| *Potorous tridactylus* | B | Boletales | Boletaceae | *Unknown* | *Unknown [longitudinally striated] Bennett and Baxter 1989* |  | 0 | Bennett and Baxter 1989 |
| *Potorous tridactylus* | u | unknown | unknown | *Unknown* | *Unknown [Opaque black, spherical spore] Claridge, A W* |  | 0 | Claridge et al. 1993 |
| *Potorous tridactylus* | B | Boletales | Boletaceae | *Unknown* | *Unknown [smooth spores] Green et al 1999* |  | 0 | Green et al. 1999 |
| *Potorous tridactylus* | B | Agaricales | unknown | *Unknown* | *Unknown [spore class 1] Tory et al. 1997* |  | 0 | Tory et al. 1997 |
| *Potorous tridactylus* | B | unknown | unknown | *Unknown* | *Unknown [spore class 12] Tory et al. 1997* |  | 0 | Tory et al. 1997 |
| *Potorous tridactylus* | B | Boletales | unknown | *Unknown* | *Unknown [spore class 2] Tory et al. 1997* |  | 0 | Tory et al. 1997 |
| *Potorous tridactylus* | B | Agaricales | unknown | *Unknown* | *Unknown [spore class 26] Tory et al. 1997* |  | 0 | Tory et al. 1997 |
| *Potorous tridactylus* | B | Agaricales | Cortinariaceae | *Unknown* | *Unknown [spore class 27]Tory et al. 1997* |  | 0 | Tory et al. 1997 |
| *Potorous tridactylus* | B | Agaricales | unknown | *Unknown* | *Unknown [spore class 28]Tory et al. 1997* |  | 0 | Tory et al. 1997 |
| *Potorous tridactylus* | B | Russulales | Russulaceae | *Unknown* | *Unknown [spore class 31]Tory et al. 1997* |  | 0 | Tory et al. 1997 |
| *Potorous tridactylus* | B | Russulales | Russulaceae | *Unknown* | *Unknown [spore class 33]Tory et al. 1997* |  | 0 | Tory et al. 1997 |
| *Potorous tridactylus* | A | Pezizales | unknown | *Unknown* | *Unknown [spore class 36]Tory et al. 1997* |  | 0 | Tory et al. 1997 |
| *Potorous tridactylus* | u | unknown | unknown | *Unknown* | *Unknown [spore class 44]Tory et al. 1997* |  | 0 | Tory et al. 1997 |
| *Potorous tridactylus* | B | unknown | unknown | *Unknown* | *Unknown [spore class 48]Tory et al. 1997* |  | 0 | Tory et al. 1997 |
| *Potorous tridactylus* | B | unknown | unknown | *Unknown* | *Unknown [spore class 49]Tory et al. 1997* |  | 0 | Tory et al. 1997 |
| *Potorous tridactylus* | B | Agaricales | Bolbitiaceae | *Unknown* | *Unknown sp.1 Claridge, A W* |  | 0 | Claridge et al. 1993 |
| *Potorous tridactylus* | B | Agaricales | Hydnangiaceae | *Unknown* | *Unknown sp.1 Claridge, A W* |  | 0 | Claridge et al. 1993 |
| *Potorous tridactylus* | B | Russulales | Russulaceae | *Unknown* | *Unknown sp.1 Claridge, A W* |  | 0 | Claridge et al. 1993 |
| *Potorous tridactylus* | B | Agaricales | Strophariaceae | *Unknown* | *Unknown sp.1 Claridge, A W* |  | 0 | Claridge et al. 1993 |
| *Potorous tridactylus* | B | Agaricales | Hydnangiaceae | *Unknown* | *Unknown sp.2 Claridge, A W* |  | 0 | Claridge et al. 1993 |
| *Potorous tridactylus* | B | Russulales | Russulaceae | *Unknown* | *Unknown sp.2 Claridge, A W* |  | 0 | Claridge et al. 1993 |
| *Potorous tridactylus* | B | Agaricales | Strophariaceae | *Unknown* | *Unknown sp.2 Claridge, A W* |  | 0 | Claridge et al. 1993 |
| *Potorous tridactylus* | B | Agaricales | Bolbitiaceae | *Unknown* | *Unknown sp.2 Claridge, A W* |  | 0 | Claridge et al. 1993 |
| *Potorous tridactylus* | u | unknown | unknown | *Unknown* | *Unknown sp.2 Vernes, K* |  | 0 | Vernes 2010 |
| *Potorous tridactylus* | B | Russulales | Russulaceae | *Unknown* | *Unknown sp.3 Claridge, A W* |  | 0 | Claridge et al. 1993 |
| *Potorous tridactylus* | B | Russulales | unknown | *Unknown* | *Unknown sp.4 Vernes, K* |  | 0 | Vernes 2010 |
| *Potorous tridactylus* | A | Pezizales | Pyronemataceae | *Unknown* | *Unknown tuberculatella* |  | 0 | Bennett and Baxter 1989 |
| *Potorous tridactylus* | B | Russulales | Russulaceae | *Zelleromyces* | *Zelleromyces* | *Zelleromyces* | 1 | Bennett and Baxter 1989 |
| *Potorous tridactylus* | B | Russulales | Russulaceae | *Zelleromyces* | *Zelleromyces* | *Zelleromyces* | 1 | Claridge et al. 1992 |
| *Potorous tridactylus* | B | Russulales | Russulaceae | *Zelleromyces* | *Zelleromyces australiensis* | *Zelleromyces australiensis* | 1 | Claridge et al. 1992 |
| *Potorous tridactylus* | B | Russulales | Russulaceae | *Zelleromyces* | *Zelleromyces daucinus* | *Zelleromyces daucinus* | 1 | Bennett and Baxter 1989 |
| *Potorous tridactylus* | B | Russulales | Russulaceae | *Zelleromyces* | *Zelleromyces daucinus* | *Zelleromyces daucinus* | 1 | Claridge et al. 1992 |
| *Potorous tridactylus* | B | Russulales | Russulaceae | *Zelleromyces* | *Zelleromyces daucinus* | *Zelleromyces daucinus* | 1 | Claridge et al. 1993 |
| *Potorous tridactylus* | B | Russulales | Russulaceae | *Zelleromyces* | *Zelleromyces daucinus* | *Zelleromyces daucinus* | 1 | Tory et al. 1997 |
| *Potorous tridactylus* | B | Russulales | Russulaceae | *Zelleromyces* | *Zelleromyces glabrellus* | *Zelleromyces glabrellus* | 1 | Bennett and Baxter 1989 |
| *Potorous tridactylus* | B | Russulales | Russulaceae | *Zelleromyces* | *Zelleromyces malaiensis* | *Zelleromyces malaiensis* | 1 | Bennett and Baxter 1989 |
| *Potorous tridactylus* | B | Russulales | Russulaceae | *Zelleromyces* | *Zelleromyces malaiensis* | *Zelleromyces malaiensis* | 1 | Claridge et al. 1992 |
| *Potorous tridactylus* | B | Russulales | Russulaceae | *Zelleromyces* | *Zelleromyces malaiensis* | *Zelleromyces malaiensis* | 1 | Claridge et al. 1993 |
| *Potorous tridactylus* | B | Russulales | Russulaceae | *Zelleromyces* | *Zelleromyces striatus* | *Zelleromyces striatus* | 1 | Bennett and Baxter 1989 |
| *Potorous tridactylus* | B | Russulales | Russulaceae | *Zelleromyces* | *Zelleromyces striatus* | *Zelleromyces striatus* | 1 | Claridge et al. 1992 |
| *Potorous tridactylus* | B | Russulales | Russulaceae | *Zelleromyces* | *Zelleromyces striatus* | *Zelleromyces striatus* | 1 | Claridge et al. 1993 |
| *Pseudomys fumeus* | B | Boletales | Boletaceae | *Chamonixia* | *Chamonixia mucosa* | *Rossbeevera mucosa* | 1 | T. May |
| *Pseudomys fumeus* | Z | Endogonales | Endogonaceae | *Endogone* | *Endogone* | *Endogone* | 1 | Ford et al. 2003 |
| *Pseudomys fumeus* | B | Agaricales | Entolomataceae | *Entoloma* | *Entoloma* | *Entoloma* | 1 | T. May |
| *Pseudomys fumeus* | B | Agaricales | Strophariaceae | *Hymenogaster* | *Hymenogaster* | *Hymenogaster* | 1 | Ford et al. 2003 |
| *Pseudomys fumeus* | B | Agaricales | Strophariaceae | *Hymenogaster* | *Hymenogaster* | *Hymenogaster* | 1 | T. May |
| *Pseudomys fumeus* | B | Agaricales | Strophariaceae | *Hymenogaster* | *Hymenogaster atratus* | *Cortinarius atratus* | 1 | T. May |
| *Pseudomys fumeus* | B | Agaricales | Strophariaceae | *Hymenogaster* | *Hymenogaster oblongisporus* | *Cortinarius oblongisporus* | 1 | T. May |
| *Pseudomys fumeus* | B | Hysterangiales | Hysterangiaceae | *Hysterangium* | *Hysterangium* | *Hysterangium* | 1 | Ford et al. 2003 |
| *Pseudomys fumeus* | B | Hysterangiales | Hysterangiaceae | *Hysterangium* | *Hysterangium* | *Hysterangium* | 1 | T. May |
| *Pseudomys fumeus* | B | Hysterangiales | Hysterangiaceae | *Hysterangium* | *Hysterangium inflatum* | *Hysterangium inflatum* | 1 | T. May |
| *Pseudomys fumeus* | B | Hysterangiales | Mesophelliaceae | *Mesophellia* | *Mesophellia* | *Mesophellia* | 1 | Ford et al. 2003 |
| *Pseudomys fumeus* | B | Hysterangiales | Mesophelliaceae | *Nothocastoreum* | *Nothocastoreum cretaceum* | *Nothocastoreum cretaceum* | 1 | T. May |
| *Pseudomys fumeus* | B | Boletales | Octavianiaceae | *Octaviania* | *Octaviania* | *Octaviania* | 1 | Ford et al. 2003 |
| *Pseudomys fumeus* | Z | Endogonales | Endogonaceae | *Unknown* | *Unknown* |  | 0 | T. May |
| *Pseudomys fumeus* | B | Russulales | Russulaceae | *Unknown* | *Unknown [hepaticus]* |  | 0 | T. May |
| *Pseudomys gracilicaudatus* | Z | Endogonales | Endogonaceae | *Endogone* | *Endogone* | *Endogone* | 1 | Luo et al. 1994 |
| *Pseudomys higginsii* | B | Hysterangiales | Hysterangiaceae | *Hysterangium* | *Hysterangium inflatum* | *Hysterangium inflatum* | 1 | T. May |
| *Pseudomys novaehollandiae* | Z | Endogonales | Endogonaceae | *Endogone* | *Endogone* | *Endogone* | 1 | Vernes and Dunn 2009 |
| *Pseudomys novaehollandiae* | B | Hysterangiales | Mesophelliaceae | *Mesophellia* | *Mesophellia* | *Mesophellia* | 1 | Wilson and Bradtke 1999 |
| *Pseudomys novaehollandiae* | u | unknown | unknown | *Unknown* | *Unknown* |  | 0 | Vernes and Dunn 2009 |
| *Pseudomys novaehollandiae* | Z | Endogonales | Endogonaceae | *Unknown* | *Unknown* |  | 0 | Wilson and Bradtke 1999 |
| *Pseudomys pilligaensis* | Z | Zygomycetes | unknown | *Densospora* | *Densospora* | *Densospora* | 1 | Tokushima and Jarman 2010 |
| *Pseudomys pilligaensis* | G | Glomerales | Glomeraceae | *Glomus* | *Glomus fulvum* | *Redeckera fulvum* | 1 | Tokushima and Jarman 2010 |
| *Pseudomys pilligaensis* | G | Glomerales | Glomeraceae | *Glomus* | *Glomus pellucidum* | *Glomus pellucidum* | 1 | Tokushima and Jarman 2010 |
| *Pseudomys pilligaensis* | A | Pleosporales | Pleosporaceae | *Stemphylium* | *Stemphylium* | *Stemphylium* | 1 | Tokushima and Jarman 2010 |
| *Pseudomys shortridgei* | A | Pezizales | Pezizaceae | *Amylascus* | *Amylascus* | *Amylascus* | 1 | T. May |
| *Pseudomys shortridgei* | B | Boletales | Boletaceae | *Chamonixia* | *Chamonixia mucosa* | *Rossbeevera mucosa* | 1 | T. May |
| *Pseudomys shortridgei* | B | Russulales | Russulaceae | *Elasmomyces* | *Elasmomyces* | *Elasmomyces* | 1 | T. May |
| *Pseudomys shortridgei* | B | Hysterangiales | Hysterangiaceae | *Hysterangium* | *Hysterangium* | *Hysterangium* | 1 | T. May |
| *Pseudomys shortridgei* | B | Hysterangiales | Hysterangiaceae | *Hysterangium* | *Hysterangium inflatum* | *Hysterangium inflatum* | 1 | T. May |
| *Pseudomys shortridgei* | Z | Endogonales | Endogonaceae | *Unknown* | *Unknown* |  | 0 | T. May |
| *Rattus fuscipes* | A | Pezizales | Pezizaceae | *Amylascus* | *Amylascus* | *Amylascus* | 1 | O’Malley 2012 |
| *Rattus fuscipes* | A | Pezizales | Pezizaceae | *Amylascus* | *Amylascus* | *Amylascus* | 1 | Vernes et al. 2015 |
| *Rattus fuscipes* | B | Russulales | Russulaceae | *Arcangeliella* | *Arcangeliella* | *Arcangeliella* | 1 | O’Malley 2012 |
| *Rattus fuscipes* | B | Agaricales | Cortinariaceae | *Aroramyces* | *Aroramyces* | *Aroramyces* | 1 | O’Malley 2012 |
| *Rattus fuscipes* | B | Agaricales | Cortinariaceae | *Aroramyces* | *Aroramyces sp.1 Vernes, K* | *Aroramyces* | 1 | Vernes et al. 2015 |
| *Rattus fuscipes* | B | Agaricales | Cortinariaceae | *Aroramyces* | *Aroramyces sp.2 Vernes, K* | *Aroramyces* | 1 | Vernes et al. 2015 |
| *Rattus fuscipes* | B | Hysterangiales | Gallaceaceae | *Austrogautieria* | *Austrogautieria* | *Austrogautieria* | 1 | O’Malley 2012 |
| *Rattus fuscipes* | B | Hysterangiales | Gallaceaceae | *Austrogautieria* | *Austrogautieria* | *Austrogautieria* | 1 | Vernes et al. 2015 |
| *Rattus fuscipes* | B | Hysterangiales | Gallaceaceae | *Austrogautieria* | *Austrogautieria macrosporus* | *Austrogautieria macrosporus* | 1 | Tory et al. 1997 |
| *Rattus fuscipes* | B | Boletales | Boletaceae | *Chamonixia* | *Chamonixia* | *Rossbeevera* | 1 | Tory et al. 1997 |
| *Rattus fuscipes* | B | Boletales | Boletaceae | *Chamonixia* | *Chamonixia* | *Rossbeevera* | 1 | Vernes et al. 2015 |
| *Rattus fuscipes* | B | Hysterangiales | Mesophelliaceae | *Chondrogaster* | *Chondrogaster* | *Chondrogaster* | 1 | O’Malley 2012 |
| *Rattus fuscipes* | B | Agaricales | Cortinariaceae | *Cortinarius* | *Cortinarius* | *Cortinarius* | 1 | O’Malley 2012 |
| *Rattus fuscipes* | B | Agaricales | Cortinariaceae | *Cortinarius* | *Cortinarius* | *Cortinarius* | 1 | Vernes et al. 2015 |
| *Rattus fuscipes* | B | Agaricales | Cortinariaceae | *Descomyces* | *Descomyces* | *Descomyces* | 1 | O’Malley 2012 |
| *Rattus fuscipes* | B | Agaricales | Cortinariaceae | *Descomyces* | *Descomyces sp.2 Vernes, K* | *Descomyces* | 1 | Vernes et al. 2015 |
| *Rattus fuscipes* | B | Agaricales | Cortinariaceae | *Descomyces* | *Descomyces sp.3 Vernes, K* | *Descomyces* | 1 | Vernes et al. 2015 |
| *Rattus fuscipes* | B | Agaricales | Cortinariaceae | *Descomyces* | *Descomyces sp.5 Vernes, K* | *Descomyces* | 1 | Vernes et al. 2015 |
| *Rattus fuscipes* | B | Agaricales | Cortinariaceae | *Descomyces* | *Descomyces stolatus* | *Descomyces stolatus* | 1 | Vernes et al. 2015 |
| *Rattus fuscipes* | B | Agaricales | Cortinariaceae | *Descomyces* | *Descomyces zeylanicus* | *Descomyces zeylanicus* | 1 | Tory et al. 1997 |
| *Rattus fuscipes* | A | Pezizales | Tuberaceae | *Dingleya* | *Dingleya* | *Dingleya* | 1 | O’Malley 2012 |
| *Rattus fuscipes* | A | Pezizales | Tuberaceae | *Dingleya* | *Dingleya* | *Dingleya* | 1 | Vernes et al. 2015 |
| *Rattus fuscipes* | A | Elaphomycetales | Elaphomycetaceae | *Elaphomyces* | *Elaphomyces* | *Elaphomyces* | 1 | O’Malley 2012 |
| *Rattus fuscipes* | A | Elaphomycetales | Elaphomycetaceae | *Elaphomyces* | *Elaphomyces* | *Elaphomyces* | 1 | Vernes et al. 2015 |
| *Rattus fuscipes* | Z | Endogonales | Endogonaceae | *Endogone* | *Endogone* | *Endogone* | 1 | McGee and Baczocha 1994 |
| *Rattus fuscipes* | Z | Endogonales | Endogonaceae | *Endogone* | *Endogone* | *Endogone* | 1 | O’Malley 2012 |
| *Rattus fuscipes* | Z | Endogonales | Endogonaceae | *Endogone* | *Endogone aggregata* | *Endogone aggregata* | 1 | McGee and Baczocha 1994 |
| *Rattus fuscipes* | B | Agaricales | Agaricaceae | *Endoptychum* | *Endoptychum* |  | 0 | Vernes et al. 2015 |
| *Rattus fuscipes* | B | Gomphales | Gomphaceae | *Gautieria* | *Gautieria monospora* | *Gautieria monospora* | 1 | Tory et al. 1997 |
| *Rattus fuscipes* | G | Glomerales | Glomeraceae | *Glomus* | *Glomus* | *Glomus* | 1 | McGee and Baczocha 1994 |
| *Rattus fuscipes* | G | Glomerales | Glomeraceae | *Glomus* | *Glomus* | *Glomus* | 1 | O’Malley 2012 |
| *Rattus fuscipes* | G | Glomerales | Glomeraceae | *Glomus* | *Glomus* | *Glomus* | 1 | Vernes and Dunn 2009 |
| *Rattus fuscipes* | G | Glomerales | Glomeraceae | *Glomus* | *Glomus* | *Glomus* | 1 | Vernes et al. 2015 |
| *Rattus fuscipes* | G | Glomerales | Glomeraceae | *Glomus* | *Glomus australe* | *Glomus australe* | 1 | McGee and Baczocha 1994 |
| *Rattus fuscipes* | G | Glomerales | Glomeraceae | *Glomus* | *Glomus fuegianum* | *Glomus fuegianum* | 1 | McGee and Baczocha 1994 |
| *Rattus fuscipes* | G | Glomerales | Glomeraceae | *Glomus* | *Glomus tenerum* | *Glomus tenerum* | 1 | McGee and Baczocha 1994 |
| *Rattus fuscipes* | B | Agaricales | Hydnangiaceae | *Hydnangium* | *Hydnangium* | *Hydnangium* | 1 | O’Malley 2012 |
| *Rattus fuscipes* | B | Agaricales | Hydnangiaceae | *Hydnangium* | *Hydnangium* | *Hydnangium* | 1 | Vernes et al. 2015 |
| *Rattus fuscipes* | B | Agaricales | Hydnangiaceae | *Hydnangium* | *Hydnangium archeri* | *Hydnangium archeri* | 1 | T. May |
| *Rattus fuscipes* | A | Pezizales | Pezizaceae | *Hydnoplicata* | *Hydnoplicata* | *Hydnoplicata* | 1 | O’Malley 2012 |
| *Rattus fuscipes* | A | Pezizales | Pezizaceae | *Hydnoplicata* | *Hydnoplicata convoluta* | *Hydnoplicata convoluta* | 1 | Vernes et al. 2015 |
| *Rattus fuscipes* | B | Agaricales | Strophariaceae | *Hymenangium* | *Hymenangium album* | *Hymenangium album* | 1 | T. May |
| *Rattus fuscipes* | B | Hysterangiales | Hysterangiaceae | *Hysterangium* | *Hysterangium* | *Hysterangium* | 1 | O’Malley 2012 |
| *Rattus fuscipes* | B | Hysterangiales | Hysterangiaceae | *Hysterangium* | *Hysterangium* | *Hysterangium* | 1 | Tory et al. 1997 |
| *Rattus fuscipes* | B | Hysterangiales | Hysterangiaceae | *Hysterangium* | *Hysterangium* | *Hysterangium* | 1 | Vernes et al. 2015 |
| *Rattus fuscipes* | B | Hysterangiales | Hysterangiaceae | *Hysterangium* | *Hysterangium inflatum* | *Hysterangium inflatum* | 1 | Vernes et al. 2015 |
| *Rattus fuscipes* | B | Hysterangiales | Hysterangiaceae | *Hysterangium* | *Hysterangium inflatum* | *Hysterangium inflatum* | 1 | T. May |
| *Rattus fuscipes* | B | Agaricales | Strophariaceae | *Hysterogaster* | *Hysterogaster sp.1 Vernes, K* | *Hysterogaster* | 1 | Vernes et al. 2015 |
| *Rattus fuscipes* | B | Agaricales | Strophariaceae | *Hysterogaster* | *Hysterogaster sp.2 Vernes, K* | *Hysterogaster* | 1 | Vernes et al. 2015 |
| *Rattus fuscipes* | B | Agaricales | Strophariaceae | *Hysterogaster* | *Hysterogaster sp.3 Vernes, K* | *Hysterogaster* | 1 | Vernes et al. 2015 |
| *Rattus fuscipes* | A | Pezizales | Tuberaceae | *Labyrinthomyces* | *Labyrinthomyces* | *Labyrinthomyces* | 1 | O’Malley 2012 |
| *Rattus fuscipes* | A | Pezizales | Tuberaceae | *Labyrinthomyces* | *Labyrinthomyces* | *Labyrinthomyces* | 1 | T. May |
| *Rattus fuscipes* | A | Pezizales | Tuberaceae | *Labyrinthomyces* | *Labyrinthomyces sp.1 Vernes, K* | *Labyrinthomyces* | 1 | Vernes et al. 2015 |
| *Rattus fuscipes* | A | Pezizales | Tuberaceae | *Labyrinthomyces* | *Labyrinthomyces sp.2 Vernes, K* | *Labyrinthomyces* | 1 | Vernes et al. 2015 |
| *Rattus fuscipes* | B | Russulales | Albatrellaceae | *Leucogaster* | *Leucogaster* | *Leucogaster* | 1 | O’Malley 2012 |
| *Rattus fuscipes* | B | Russulales | Albatrellaceae | *Leucogaster* | *Leucogaster* | *Leucogaster* | 1 | Vernes et al. 2015 |
| *Rattus fuscipes* | B | Hysterangiales | Mesophelliaceae | *Mesophellia* | *Mesophellia* | *Mesophellia* | 1 | O’Malley 2012 |
| *Rattus fuscipes* | A | Pezizales | Pezizaceae | *Muciturbo* | *Muciturbo reticulatus* | *Ruhlandiella reticulata* | 1 | Tory et al. 1997 |
| *Rattus fuscipes* | B | Boletales | Octavianiaceae | *Octaviania* | *Octaviania* | *Octaviania* | 1 | O’Malley 2012 |
| *Rattus fuscipes* | B | Boletales | Octavianiaceae | *Octaviania* | *Octaviania* | *Octaviania* | 1 | Vernes et al. 2015 |
| *Rattus fuscipes* | A | Pezizales | Pezizaceae | *Peziza* | *Peziza whitei* | *Hydnoplicata convoluta* | 1 | Tory et al. 1997 |
| *Rattus fuscipes* | B | unknown | unknown | *Pogisperma* | *Pogisperma* | *Pogisperma* | 1 | O’Malley 2012 |
| *Rattus fuscipes* | B | Phallales | Protophallaceae | *Protubera* | *Protubera* | *Protubera* | 1 | Vernes et al. 2015 |
| *Rattus fuscipes* | B | Boletales | Boletaceae | *Rossbeevera* | *Rossbeevera* | *Rossbeevera* | 1 | O’Malley 2012 |
| *Rattus fuscipes* | B | Boletales | Sclerodermataceae | *Scleroderma* | *Scleroderma* | *Scleroderma* | 1 | O’Malley 2012 |
| *Rattus fuscipes* | B | Boletales | Sclerodermataceae | *Scleroderma* | *Scleroderma* | *Scleroderma* | 1 | Vernes et al. 2015 |
| *Rattus fuscipes* | B | Boletales | Sclerodermataceae | *Scleroderma* | *Scleroderma tommayi* | *Scleroderma tommayi* | 1 | Vernes et al. 2015 |
| *Rattus fuscipes* | B | Geastrales | Geastraceae | *Sclerogaster* | *Sclerogaster* | *Sclerogaster* | 1 | O’Malley 2012 |
| *Rattus fuscipes* | A | Pezizales | Pyronemataceae | *Sphaerosoma* | *Sphaerosoma* | *Sphaerosoma* | 1 | Vernes et al. 2015 |
| *Rattus fuscipes* | A | Pezizales | Pezizaceae | *Sphaerozone* | *Sphaerozone echinulatum* | *Gymnohydnotrya echinulata* | 1 | T. May |
| *Rattus fuscipes* | B | Agaricales | Stephanosporaceae | *Stephanospora* | *Stephanospora* | *Stephanospora* | 1 | O’Malley 2012 |
| *Rattus fuscipes* | B | Agaricales | Cortinariaceae | *Thaxterogaster* | *Thaxterogaster sp.1 Vernes, K* | *Cortinarius* | 1 | Vernes et al. 2015 |
| *Rattus fuscipes* | B | Agaricales | Cortinariaceae | *Thaxterogaster* | *Thaxterogaster sp.2 Vernes, K* | *Cortinarius* | 1 | Vernes et al. 2015 |
| *Rattus fuscipes* | B | Agaricales | Cortinariaceae | *Thaxterogaster* | *Thaxterogaster sp.3 Vernes, K* | *Cortinarius* | 1 | Vernes et al. 2015 |
| *Rattus fuscipes* | B | Agaricales | unknown | *Timgrovea* | *Timgrovea* | *Timgrovea* | 1 | O’Malley 2012 |
| *Rattus fuscipes* | B | Hysterangiales | Mesophelliaceae | *Unknown* | *Unknown* |  | 0 | O’Malley 2012 |
| *Rattus fuscipes* | B | Russulales | Russulaceae | *Unknown* | *Unknown* |  | 0 | O’Malley 2012 |
| *Rattus fuscipes* | u | unknown | unknown | *Unknown* | *Unknown* |  | 0 | Tory et al. 1997 |
| *Rattus fuscipes* | A | unknown | unknown | *Unknown* | *Unknown* |  | 0 | Vernes and Dunn 2009 |
| *Rattus fuscipes* | B | Agaricales | Coprinaceae | *Unknown* | *Unknown* |  | 0 | Vernes et al. 2015 |
| *Rattus fuscipes* | B | Hysterangiales | Mesophelliaceae | *Unknown* | *Unknown* |  | 0 | Vernes et al. 2015 |
| *Rattus fuscipes* | u | unknown | unknown | *Unknown* | *Unknown* |  | 0 | Vernes et al. 2015 |
| *Rattus fuscipes* | Z | Endogonales | Endogonaceae | *Unknown* | *Unknown* |  | 0 | T. May |
| *Rattus fuscipes* | u | unknown | unknown | *Unknown* | *Unknown [epigeous mushroom] Vernes, K* |  | 0 | Vernes and Dunn 2009 |
| *Rattus fuscipes* | u | unknown | unknown | *Unknown* | *Unknown [hypogeous ECM] Vernes, K* |  | 0 | Vernes and Dunn 2009 |
| *Rattus fuscipes* | B | Agaricales | unknown | *Unknown* | *Unknown [spore class 1] Tory et al. 1997* |  | 0 | Tory et al. 1997 |
| *Rattus fuscipes* | B | unknown | unknown | *Unknown* | *Unknown [spore class 12] Tory et al. 1997* |  | 0 | Tory et al. 1997 |
| *Rattus fuscipes* | B | Phallales | unknown | *Unknown* | *Unknown [spore class 19] Tory et al. 1997* |  | 0 | Tory et al. 1997 |
| *Rattus fuscipes* | B | Boletales | unknown | *Unknown* | *Unknown [spore class 2] Tory et al. 1997* |  | 0 | Tory et al. 1997 |
| *Rattus fuscipes* | B | Agaricales | unknown | *Unknown* | *Unknown [spore class 26] Tory et al. 1997* |  | 0 | Tory et al. 1997 |
| *Rattus fuscipes* | B | Agaricales | Cortinariaceae | *Unknown* | *Unknown [spore class 27]Tory et al. 1997* |  | 0 | Tory et al. 1997 |
| *Rattus fuscipes* | B | Agaricales | unknown | *Unknown* | *Unknown [spore class 28]Tory et al. 1997* |  | 0 | Tory et al. 1997 |
| *Rattus fuscipes* | B | Russulales | Russulaceae | *Unknown* | *Unknown [spore class 31]Tory et al. 1997* |  | 0 | Tory et al. 1997 |
| *Rattus fuscipes* | B | Russulales | Russulaceae | *Unknown* | *Unknown [spore class 33]Tory et al. 1997* |  | 0 | Tory et al. 1997 |
| *Rattus fuscipes* | A | Pezizales | unknown | *Unknown* | *Unknown [spore class 36]Tory et al. 1997* |  | 0 | Tory et al. 1997 |
| *Rattus fuscipes* | B | Russulales | Russulaceae | *Unknown* | *Unknown [spore class 4] Tory et al. 1997* |  | 0 | Tory et al. 1997 |
| *Rattus fuscipes* | u | unknown | unknown | *Unknown* | *Unknown [spore class 44]Tory et al. 1997* |  | 0 | Tory et al. 1997 |
| *Rattus fuscipes* | B | unknown | unknown | *Unknown* | *Unknown [spore class 45]Tory et al. 1997* |  | 0 | Tory et al. 1997 |
| *Rattus fuscipes* | B | unknown | unknown | *Unknown* | *Unknown [spore class 48]Tory et al. 1997* |  | 0 | Tory et al. 1997 |
| *Rattus fuscipes* | B | unknown | unknown | *Unknown* | *Unknown [spore class 49]Tory et al. 1997* |  | 0 | Tory et al. 1997 |
| *Rattus fuscipes* | A | unknown | unknown | *Unknown* | *Unknown sp.1 Vernes, K* |  | 0 | Vernes et al. 2015 |
| *Rattus fuscipes* | B | Boletales | unknown | *Unknown* | *Unknown sp.1 Vernes, K* |  | 0 | Vernes et al. 2015 |
| *Rattus fuscipes* | B | Russulales | unknown | *Unknown* | *Unknown sp.1 Vernes, K* |  | 0 | Vernes et al. 2015 |
| *Rattus fuscipes* | u | unknown | unknown | *Unknown* | *Unknown sp.13 Vernes, K* |  | 0 | Vernes et al. 2015 |
| *Rattus fuscipes* | A | unknown | unknown | *Unknown* | *Unknown sp.2 Vernes, K* |  | 0 | Vernes et al. 2015 |
| *Rattus fuscipes* | B | Boletales | unknown | *Unknown* | *Unknown sp.2 Vernes, K* |  | 0 | Vernes et al. 2015 |
| *Rattus fuscipes* | B | Russulales | unknown | *Unknown* | *Unknown sp.2 Vernes, K* |  | 0 | Vernes et al. 2015 |
| *Rattus fuscipes* | B | Russulales | unknown | *Unknown* | *Unknown sp.3 Vernes, K* |  | 0 | Vernes et al. 2015 |
| *Rattus fuscipes* | B | Russulales | unknown | *Unknown* | *Unknown sp.4 Vernes, K* |  | 0 | Vernes et al. 2015 |
| *Rattus fuscipes* | u | unknown | unknown | *Unknown* | *Unknown sp.4 Vernes, K* |  | 0 | Vernes et al. 2015 |
| *Rattus fuscipes* | u | unknown | unknown | *Unknown* | *Unknown sp.6 Vernes, K* |  | 0 | Vernes et al. 2015 |
| *Rattus fuscipes* | u | unknown | unknown | *Unknown* | *Unknown sp.7 Vernes, K* |  | 0 | Vernes et al. 2015 |
| *Rattus fuscipes* | u | unknown | unknown | *Unknown* | *Unknown sp.8 Vernes, K* |  | 0 | Vernes et al. 2015 |
| *Rattus fuscipes* | B | Russulales | Russulaceae | *Zelleromyces* | *Zelleromyces daucinus* | *Zelleromyces daucinus* | 1 | Tory et al. 1997 |
| *Rattus fuscipes* | B | Russulales | Russulaceae | *Zelleromyces* | *Zelleromyces striatus* | *Zelleromyces striatus* | 1 | T. May |
| *Rattus rattus* | Z | Zygomycetes | unknown | *Densospora* | *Densospora tubiforme nom. Ined.* | *Densospora tubiforme* | 1 | McGee and Baczocha 1994 |
| *Rattus rattus* | Z | Endogonales | Endogonaceae | *Endogone* | *Endogone aggregata* | *Endogone aggregata* | 1 | McGee and Baczocha 1994 |
| *Rattus rattus* | G | Diversisporales | Gigasporaceae | *Gigaspora* | *Gigaspora* | *Gigaspora* | 1 | McGee and Baczocha 1994 |
| *Rattus rattus* | G | Glomerales | Glomeraceae | *Glomus* | *Glomus* | *Glomus* | 1 | McGee and Baczocha 1994 |
| *Rattus rattus* | G | Glomerales | Glomeraceae | *Glomus* | *Glomus affin. Cerebriforme* | *Glomus affin. Cerebriforme* | 1 | McGee and Baczocha 1994 |
| *Rattus rattus* | G | Glomerales | Glomeraceae | *Glomus* | *Glomus australe* | *Glomus australe* | 1 | McGee and Baczocha 1994 |
| *Rattus rattus* | G | Glomerales | Glomeraceae | *Glomus* | *Glomus fuegianum* | *Glomus fuegianum* | 1 | McGee and Baczocha 1994 |
| *Rattus rattus* | G | Glomerales | Glomeraceae | *Glomus* | *Glomus macrocarpus* | *Glomus macrocarpum* | 1 | McGee and Baczocha 1994 |
| *Rattus rattus* | G | Glomerales | Glomeraceae | *Glomus* | *Glomus sp.1 McGee and Baczocha 1994* | *Glomus* | 1 | McGee and Baczocha 1994 |
| *Rattus rattus* | G | Glomerales | Glomeraceae | *Glomus* | *Glomus sp.2 McGee and Baczocha 1994* | *Glomus* | 1 | McGee and Baczocha 1994 |
| *Rattus rattus* | G | Glomerales | Glomeraceae | *Glomus* | *Glomus sp.3 McGee and Baczocha 1994* | *Glomus* | 1 | McGee and Baczocha 1994 |
| *Rattus rattus* | G | Glomerales | Glomeraceae | *Glomus* | *Glomus sp.4 McGee and Baczocha 1994* | *Glomus* | 1 | McGee and Baczocha 1994 |
| *Rattus rattus* | G | Glomerales | Glomeraceae | *Glomus* | *Glomus sp.5 McGee and Baczocha 1994* | *Glomus* | 1 | McGee and Baczocha 1994 |
| *Rattus villosissimus* | G | Glomerales | Glomeraceae | *Glomus* | *Glomus sp.6 McGee and Baczocha 1994* | *Glomus* | 1 | McGee and Baczocha 1994 |
| *Thylogale stigmatica* | B | Boletales | Boletaceae | *Chamonixia* | *Chamonixia* | *Rossbeevera* | 1 | Vernes and Trappe 2007 |
| *Thylogale stigmatica* | B | Agaricales | Physalacriaceae | *Cribbea* | *Cribbea* | *Cribbea* | 1 | Vernes and Trappe 2007 |
| *Thylogale stigmatica* | B | Gomphales | Gomphaceae | *Gautieria* | *Gautieria* | *Gautieria* | 1 | Vernes and Trappe 2007 |
| *Thylogale stigmatica* | A | Pezizales | Discinaceae | *Gymnohydnotrya* | *Gymnohydnotrya* | *Gymnohydnotrya* | 1 | Vernes and Trappe 2007 |
| *Thylogale stigmatica* | A | Pezizales | Pezizaceae | *Hydnoplicata* | *Hydnoplicata* | *Hydnoplicata* | 1 | Vernes and Trappe 2007 |
| *Thylogale stigmatica* | B | Hysterangiales | Hysterangiaceae | *Hysterangium* | *Hysterangium* | *Hysterangium* | 1 | Vernes and Trappe 2007 |
| *Thylogale stigmatica* | A | Hypocreales | Ceratostomataceae | *Microthecium* | *Microthecium beatonii* | *Sphaerodes beatonii* | 1 | Vernes and Trappe 2007 |
| *Thylogale stigmatica* | A | Pezizales | Pyronemataceae | *Sphaerosoma* | *Sphaerosoma* | *Sphaerosoma* | 1 | Vernes and Trappe 2007 |
| *Thylogale stigmatica* | B | Agaricales | Stephanosporaceae | *Stephanospora* | *Stephanospora flava* | *Stephanospora flava* | 1 | Vernes and Trappe 2007 |
| *Thylogale stigmatica* | A | Pezizales | Tuberaceae | *Unknown* | *Unknown* |  | 0 | Vernes and Trappe 2007 |
| *Thylogale stigmatica* | B | Agaricales | Cortinariaceae | *Unknown* | *Unknown* |  | 0 | Vernes and Trappe 2007 |
| *Thylogale stigmatica* | B | Boletales | Octavianiaceae | *Unknown* | *Unknown* |  | 0 | Vernes and Trappe 2007 |
| *Thylogale stigmatica* | B | Russulales | Russulaceae | *Unknown* | *Unknown sp.1 (possibly Gymnomyces) Vernes, K* |  | 0 | Vernes and Trappe 2007 |
| *Thylogale stigmatica* | u | unknown | unknown | *Unknown* | *Unknown sp.1 Vernes, K* |  | 0 | Vernes and Trappe 2007 |
| *Thylogale stigmatica* | B | Russulales | Russulaceae | *Unknown* | *Unknown sp.2 Vernes, K* |  | 0 | Vernes and Trappe 2007 |
| *Thylogale stigmatica* | u | unknown | unknown | *Unknown* | *Unknown sp.2 Vernes, K* |  | 0 | Vernes and Trappe 2007 |
| *Thylogale stigmatica* | B | Russulales | Russulaceae | *Unknown* | *Unknown sp.3 Vernes, K* |  | 0 | Vernes and Trappe 2007 |
| *Thylogale stigmatica* | u | unknown | unknown | *Unknown* | *Unknown sp.3 Vernes, K* |  | 0 | Vernes and Trappe 2007 |
| *Thylogale stigmatica* | u | unknown | unknown | *Unknown* | *Unknown sp.4 Vernes, K* |  | 0 | Vernes and Trappe 2007 |
| *Thylogale stigmatica* | u | unknown | unknown | *Unknown* | *Unknown sp.5 (possibly Octaviania) Vernes, K* |  | 0 | Vernes and Trappe 2007 |
| *Thylogale stigmatica* | u | unknown | unknown | *Unknown* | *Unknown sp.6 Vernes, K* |  | 0 | Vernes and Trappe 2007 |
| *Thylogale stigmatica* | u | unknown | unknown | *Unknown* | *Unknown sp.7 Vernes, K* |  | 0 | Vernes and Trappe 2007 |
| *Thylogale thetis* | B | Agaricales | Agaricaceae | *Agaricus* | *Agaricus sp.1 Vernes, K* | *Agaricus* | 1 | Vernes 2010 |
| *Thylogale thetis* | B | Hysterangiales | Gallaceaceae | *Austrogautieria* | *Austrogautieria* | *Austrogautieria* | 1 | Vernes 2010 |
| *Thylogale thetis* | B | Boletales | Boletaceae | *Boletellus* | *Boletellus* | *Boletellus* | 1 | Vernes 2010 |
| *Thylogale thetis* | B | Boletales | Boletaceae | *Chamonixia* | *Chamonixia* | *Rossbeevera* | 1 | Vernes 2010 |
| *Thylogale thetis* | B | Agaricales | Cortinariaceae | *Descomyces* | *Descomyces sp.2 Vernes, K* | *Descomyces* | 1 | Vernes 2010 |
| *Thylogale thetis* | B | Agaricales | Cortinariaceae | *Descomyces* | *Descomyces sp.3 Vernes, K* | *Descomyces* | 1 | Vernes 2010 |
| *Thylogale thetis* | B | Agaricales | Cortinariaceae | *Descomyces* | *Descomyces stolatus* | *Descomyces stolatus* | 1 | Vernes 2010 |
| *Thylogale thetis* | A | Elaphomycetales | Elaphomycetaceae | *Elaphomyces* | *Elaphomyces* | *Elaphomyces* | 1 | Vernes 2010 |
| *Thylogale thetis* | G | Glomerales | Glomeraceae | *Glomus* | *Glomus* | *Glomus* | 1 | Vernes 2010 |
| *Thylogale thetis* | A | Pezizales | Pezizaceae | *Hydnoplicata* | *Hydnoplicata* | *Hydnoplicata* | 1 | Vernes 2010 |
| *Thylogale thetis* | B | Hysterangiales | Hysterangiaceae | *Hysterangium* | *Hysterangium* | *Hysterangium* | 1 | Vernes 2010 |
| *Thylogale thetis* | B | Agaricales | Strophariaceae | *Hysterogaster* | *Hysterogaster sp.1 Vernes, K* | *Hysterogaster* | 1 | Vernes 2010 |
| *Thylogale thetis* | A | Pezizales | Tuberaceae | *Labyrinthomyces* | *Labyrinthomyces sp.2 Vernes, K* | *Labyrinthomyces* | 1 | Vernes 2010 |
| *Thylogale thetis* | B | Boletales | Octavianiaceae | *Octaviania* | *Octaviania* | *Octaviania* | 1 | Vernes 2010 |
| *Thylogale thetis* | B | Boletales | Sclerodermataceae | *Scleroderma* | *Scleroderma sp.1 Vernes, K* | *Scleroderma* | 1 | Vernes 2010 |
| *Thylogale thetis* | B | Boletales | Sclerodermataceae | *Scleroderma* | *Scleroderma sp.2 Vernes, K* | *Scleroderma* | 1 | Vernes 2010 |
| *Thylogale thetis* | B | Boletales | Sclerodermataceae | *Scleroderma* | *Scleroderma sp.3 Vernes, K* | *Scleroderma* | 1 | Vernes 2010 |
| *Thylogale thetis* | B | Geastrales | Geastraceae | *Sclerogaster* | *Sclerogaster* | *Sclerogaster* | 1 | Vernes 2010 |
| *Thylogale thetis* | B | Agaricales | Cortinariaceae | *Thaxterogaster* | *Thaxterogaster* | *Cortinarius* | 1 | Vernes 2010 |
| *Thylogale thetis* | B | Agaricales | Coprinaceae | *Unknown* | *Unknown* |  | 0 | Vernes 2010 |
| *Thylogale thetis* | B | Boletales | unknown | *Unknown* | *Unknown sp.1 Vernes, K* |  | 0 | Vernes 2010 |
| *Thylogale thetis* | u | unknown | unknown | *Unknown* | *Unknown sp.1 Vernes, K* |  | 0 | Vernes 2010 |
| *Thylogale thetis* | B | Boletales | unknown | *Unknown* | *Unknown sp.2 Vernes, K* |  | 0 | Vernes 2010 |
| *Thylogale thetis* | B | Russulales | unknown | *Unknown* | *Unknown sp.2 Vernes, K* |  | 0 | Vernes 2010 |
| *Thylogale thetis* | u | unknown | unknown | *Unknown* | *Unknown sp.2 Vernes, K* |  | 0 | Vernes 2010 |
| *Thylogale thetis* | B | Russulales | unknown | *Unknown* | *Unknown sp.3 Vernes, K* |  | 0 | Vernes 2010 |
| *Thylogale thetis* | B | Russulales | unknown | *Unknown* | *Unknown sp.4 Vernes, K* |  | 0 | Vernes 2010 |
| *Trichosurus caninus* | B | Boletales | Boletaceae | *Chamonixia* | *Chamonixia vittatispora* | *Rossbeevera vittatispora* | 1 | Claridge and Lindenmayer 1993 |
| *Trichosurus caninus* | B | Boletales | Boletaceae | *Chamonixia* | *Chamonixia vittatispora* | *Rossbeevera vittatispora* | 1 | Claridge and Lindenmayer 1998 |
| *Trichosurus caninus* | A | Elaphomycetales | Elaphomycetaceae | *Elaphomyces* | *Elaphomyces* | *Elaphomyces* | 1 | Vernes et al. 2015 |
| *Trichosurus caninus* | Z | Endogonales | Endogonaceae | *Endogone* | *Endogone* | *Endogone* | 1 | Claridge and Lindenmayer 1998 |
| *Trichosurus caninus* | Z | Endogonales | Endogonaceae | *Endogone* | *Endogone [spore walls double-layered] Claridge, A W* | *Endogone* | 1 | Claridge and Lindenmayer 1993 |
| *Trichosurus caninus* | B | Gomphales | Gomphaceae | *Gautieria* | *Gautieria* | *Gautieria* | 1 | Claridge and Lindenmayer 1998 |
| *Trichosurus caninus* | B | Gomphales | Gomphaceae | *Gautieria* | *Gautieria sp.1 Claridge and Lindenmayer* | *Gautieria* | 1 | Claridge and Lindenmayer 1998 |
| *Trichosurus caninus* | B | Agaricales | Hydnangiaceae | *Hydnangium* | *Hydnangium* | *Hydnangium* | 1 | Claridge and Lindenmayer 1993 |
| *Trichosurus caninus* | B | Agaricales | Hydnangiaceae | *Hydnangium* | *Hydnangium carneum* | *Hydnangium carneum* | 1 | Claridge and Lindenmayer 1998 |
| *Trichosurus caninus* | B | Agaricales | Strophariaceae | *Hymenogaster* | *Hymenogaster* | *Hymenogaster* | 1 | Claridge and Lindenmayer 1998 |
| *Trichosurus caninus* | B | Agaricales | Strophariaceae | *Hymenogaster* | *Hymenogaster nanus* | *Hymenogaster nanus* | 1 | Claridge and Lindenmayer 1993 |
| *Trichosurus caninus* | B | Agaricales | Strophariaceae | *Hymenogaster* | *Hymenogaster zeylanicus* | *Descomyces albellus* | 1 | Claridge and Lindenmayer 1993 |
| *Trichosurus caninus* | A | Pezizales | Pyronemataceae | *Jafneadelphus* | *Jafneadelphus* | *Jafneadelphus* | 1 | Claridge and Lindenmayer 1993 |
| *Trichosurus caninus* | A | Pezizales | Pyronemataceae | *Jafneadelphus* | *Jafneadelphus* | *Jafneadelphus* | 1 | Claridge and Lindenmayer 1998 |
| *Trichosurus caninus* | A | Pezizales | Tuberaceae | *Labyrinthomyces* | *Labyrinthomyces* | *Labyrinthomyces* | 1 | Claridge and Lindenmayer 1998 |
| *Trichosurus caninus* | B | Hysterangiales | Mesophelliaceae | *Mesophellia* | *Mesophellia* | *Mesophellia* | 1 | Claridge and Lindenmayer 1993 |
| *Trichosurus caninus* | B | Hysterangiales | Mesophelliaceae | *Mesophellia* | *Mesophellia* | *Mesophellia* | 1 | Claridge and Lindenmayer 1998 |
| *Trichosurus caninus* | B | Boletales | Octavianiaceae | *Octaviania* | *Octaviania* | *Octaviania* | 1 | Claridge and Lindenmayer 1998 |
| *Trichosurus caninus* | B | Agaricales | Hydnangiaceae | *Podohydnangium* | *Podohydnangium* | *Podohydnangium* | 1 | Claridge and Lindenmayer 1998 |
| *Trichosurus caninus* | B | Boletales | Sclerodermataceae | *Scleroderma* | *Scleroderma* | *Scleroderma* | 1 | Vernes et al. 2015 |
| *Trichosurus caninus* | B | Agaricales | Stephanosporaceae | *Stephanospora* | *Stephanospora flava* | *Stephanospora flava* | 1 | Claridge and Lindenmayer 1993 |
| *Trichosurus caninus* | B | Agaricales | Stephanosporaceae | *Stephanospora* | *Stephanospora flava* | *Stephanospora flava* | 1 | Claridge and Lindenmayer 1998 |
| *Trichosurus caninus* | B | Agaricales | Cortinariaceae | *Thaxterogaster* | *Thaxterogaster* | *Cortinarius* | 1 | Claridge and Lindenmayer 1998 |
| *Trichosurus caninus* | B | Agaricales | Cortinariaceae | *Thaxterogaster* | *Thaxterogaster sp.1 Claridge, A W* | *Cortinarius* | 1 | Claridge and Lindenmayer 1993 |
| *Trichosurus caninus* | B | Agaricales | Cortinariaceae | *Thaxterogaster* | *Thaxterogaster sp.1 Vernes, K* | *Cortinarius* | 1 | Vernes et al. 2015 |
| *Trichosurus caninus* | B | Agaricales | Cortinariaceae | *Thaxterogaster* | *Thaxterogaster sp.2 Claridge and Lindenmayer* | *Cortinarius* | 1 | Claridge and Lindenmayer 1993 |
| *Trichosurus caninus* | B | Agaricales | Gasteromycetes | *Unknown* | *Unknown* |  | 0 | Claridge and Lindenmayer 1993 |
| *Trichosurus caninus* | Z | Endogonales | Endogonaceae | *Unknown* | *Unknown* |  | 0 | Claridge and Lindenmayer 1993 |
| *Trichosurus caninus* | u | unknown | unknown | *Unknown* | *Unknown* |  | 0 | Claridge and Lindenmayer 1998 |
| *Trichosurus caninus* | B | Agaricales | Coprinaceae | *Unknown* | *Unknown* |  | 0 | Vernes et al. 2015 |
| *Trichosurus caninus* | u | unknown | unknown | *Unknown* | *Unknown* |  | 0 | Vernes et al. 2015 |
| *Trichosurus caninus* | B | Russulales | Russulaceae | *Zelleromyces* | *Zelleromyces* | *Zelleromyces* | 1 | Claridge and Lindenmayer 1998 |
| *Uromys caudimaculatus* | Z | Endogonales | Endogonaceae | *Endogone* | *Endogone* | *Endogone* | 1 | Reddell et al. 1997 |
| *Uromys caudimaculatus* | B | Gomphales | Gomphaceae | *Gautieria* | *Gautieria amara* | *Austrogautieria amara* | 1 | Comport 2000 |
| *Uromys caudimaculatus* | G | Glomerales | Glomeraceae | *Glomus* | *Glomus* | *Glomus* | 1 | Comport 2000 |
| *Uromys caudimaculatus* | B | Hysterangiales | Mesophelliaceae | *Gummiglobus* | *Gummiglobus* | *Gummiglobus* | 1 | Comport 2000 |
| *Uromys caudimaculatus* | B | Hysterangiales | Mesophelliaceae | *Gummiglobus* | *Gummiglobus* | *Gummiglobus* | 1 | Gordon and Comport 1998 |
| *Uromys caudimaculatus* | B | Hysterangiales | Mesophelliaceae | *Gummiglobus* | *Gummiglobus* | *Gummiglobus* | 1 | Reddell et al. 1997 |
| *Uromys caudimaculatus* | B | Agaricales | Strophariaceae | *Hymenogaster* | *Hymenogaster* | *Hymenogaster* | 1 | Comport 2000 |
| *Uromys caudimaculatus* | B | Hysterangiales | Hysterangiaceae | *Hysterangium* | *Hysterangium* | *Hysterangium* | 1 | Reddell et al. 1997 |
| *Uromys caudimaculatus* | B | Hysterangiales | Mesophelliaceae | *Mesophellia* | *Mesophellia* | *Mesophellia* | 1 | Comport 2000 |
| *Uromys caudimaculatus* | B | unknown | unknown | *Pseudohysterangium* | *Pseudohysterangium* | *Pseudohysterangium* | 1 | Reddell et al. 1997 |
| *Uromys caudimaculatus* | B | Boletales | Sclerodermataceae | *Scleroderma* | *Scleroderma* | *Scleroderma* | 1 | Comport 2000 |
| *Uromys caudimaculatus* | B | Boletales | Sclerodermataceae | *Scleroderma* | *Scleroderma* | *Scleroderma* | 1 | Gordon and Comport 1998 |
| *Uromys caudimaculatus* | B | Agaricales | Cortinariaceae | *Unknown* | *Unknown* |  | 0 | Gordon and Comport 1998 |
| *Uromys caudimaculatus* | B | Agaricales | Cortinariaceae | *Unknown* | *Unknown* |  | 0 | Reddell et al. 1997 |
| *Uromys caudimaculatus* | B | Hysterangiales | Mesophelliaceae | *Unknown* | *Unknown* |  | 0 | Reddell et al. 1997 |
| *Uromys caudimaculatus* | B | Agaricales | Russulaceae | *Unknown* | *Unknown* |  | 0 | Reddell et al. 1997 |
| *Uromys caudimaculatus* | u | unknown | unknown | *Unknown* | *Unknown sp.1 Comport, SS* |  | 0 | Comport 2000 |
| *Uromys caudimaculatus* | u | unknown | unknown | *Unknown* | *Unknown sp.1 Gordon and Comport. 1998* |  | 0 | Gordon and Comport 1998 |
| *Uromys caudimaculatus* | u | unknown | unknown | *Unknown* | *Unknown sp.2 Comport, SS* |  | 0 | Comport 2000 |
| *Uromys caudimaculatus* | u | unknown | unknown | *Unknown* | *Unknown sp.2 Gordon and Comport. 1998* |  | 0 | Gordon and Comport 1998 |
| *Uromys caudimaculatus* | u | unknown | unknown | *Unknown* | *Unknown sp.3 Comport, SS* |  | 0 | Comport 2000 |
| *Uromys caudimaculatus* | u | unknown | unknown | *Unknown* | *Unknown sp.3 Gordon and Comport. 1998* |  | 0 | Gordon and Comport 1998 |
| *Uromys caudimaculatus* | u | unknown | unknown | *Unknown* | *Unknown sp.4 Gordon and Comport. 1998* |  | 0 | Gordon and Comport 1998 |
| *Uromys caudimaculatus* | B | Russulales | Russulaceae | *Zelleromyces* | *Zelleromyces* | *Zelleromyces* | 1 | Gordon and Comport 1998 |
| *Uromys caudimaculatus* | B | Russulales | Russulaceae | *Zelleromyces* | *Zelleromyces sp.1 Comport, SS* | *Zelleromyces* | 1 | Comport 2000 |
| *Uromys caudimaculatus* | B | Russulales | Russulaceae | *Zelleromyces* | *Zelleromyces sp.1 Gordon and Comport. 1998* | *Zelleromyces* | 1 | Gordon and Comport 1998 |
| *Uromys caudimaculatus* | B | Russulales | Russulaceae | *Zelleromyces* | *Zelleromyces sp.2 Comport, SS* | *Zelleromyces* | 1 | Comport 2000 |
| *Uromys caudimaculatus* | B | Russulales | Russulaceae | *Zelleromyces* | *Zelleromyces sp.2 Gordon and Comport. 1998* | *Zelleromyces* | 1 | Gordon and Comport 1998 |
| *Wallabia bicolor* | B | Agaricales | Agaricaceae | *Agaricus* | *Agaricus sp.1 Vernes, K* | *Agaricus* | 1 | Vernes 2010 |
| *Wallabia bicolor* | B | Agaricales | Agaricaceae | *Agaricus* | *Agaricus sp.2 Vernes, K* | *Agaricus* | 1 | Vernes 2010 |
| *Wallabia bicolor* | B | Russulales | Russulaceae | *Arcangeliella* | *Arcangeliella sp.1 O'Malley, A* | *Arcangeliella* | 1 | O’Malley 2012 |
| *Wallabia bicolor* | B | Russulales | Russulaceae | *Arcangeliella* | *Arcangeliella sp.2 O'Malley, A* | *Arcangeliella* | 1 | O’Malley 2012 |
| *Wallabia bicolor* | B | Russulales | Russulaceae | *Arcangeliella* | *Arcangeliella sp.3 O'Malley, A* | *Arcangeliella* | 1 | O’Malley 2012 |
| *Wallabia bicolor* | B | Agaricales | Cortinariaceae | *Aroramyces* | *Aroramyces sp.1 O'Malley, A* | *Aroramyces* | 1 | O’Malley 2012 |
| *Wallabia bicolor* | B | Agaricales | Cortinariaceae | *Aroramyces* | *Aroramyces sp.1 Vernes, K* | *Aroramyces* | 1 | O’Malley 2012 |
| *Wallabia bicolor* | B | Agaricales | Cortinariaceae | *Aroramyces* | *Aroramyces sp.1 Vernes, K* | *Aroramyces* | 1 | Vernes 2010 |
| *Wallabia bicolor* | B | Agaricales | Cortinariaceae | *Aroramyces* | *Aroramyces sp.3 Danks, M* | *Aroramyces* | 1 | Danks 2011 |
| *Wallabia bicolor* | B | Hysterangiales | Gallaceaceae | *Austrogautieria* | *Austrogautieria* | *Austrogautieria* | 1 | Claridge et al. 2001 |
| *Wallabia bicolor* | B | Hysterangiales | Gallaceaceae | *Austrogautieria* | *Austrogautieria* | *Austrogautieria* | 1 | Vernes 2010 |
| *Wallabia bicolor* | B | Hysterangiales | Gallaceaceae | *Austrogautieria* | *Austrogautieria aff manjimupana* | *Austrogautieria aff manjimupana* | 1 | Danks 2011 |
| *Wallabia bicolor* | B | Hysterangiales | Gallaceaceae | *Austrogautieria* | *Austrogautieria clelandii* | *Austrogautieria clelandii* | 1 | Danks 2011 |
| *Wallabia bicolor* | B | Hysterangiales | Gallaceaceae | *Austrogautieria* | *Austrogautieria sp.1 Danks, M* | *Austrogautieria* | 1 | Danks 2011 |
| *Wallabia bicolor* | B | Hysterangiales | Gallaceaceae | *Austrogautieria* | *Austrogautieria sp.1 O'Malley, A* | *Austrogautieria* | 1 | O’Malley 2012 |
| *Wallabia bicolor* | B | Hysterangiales | Gallaceaceae | *Austrogautieria* | *Austrogautieria sp.3 Danks, M* | *Austrogautieria* | 1 | Danks 2011 |
| *Wallabia bicolor* | B | Hysterangiales | Gallaceaceae | *Austrogautieria* | *Austrogautieria sp.5 Danks, M* | *Austrogautieria* | 1 | Danks 2011 |
| *Wallabia bicolor* | B | Hysterangiales | Gallaceaceae | *Austrogautieria* | *Austrogautieria sp.7 Danks, M* | *Austrogautieria* | 1 | Danks 2011 |
| *Wallabia bicolor* | B | Boletales | Boletaceae | *Boletellus* | *Boletellus* | *Boletellus* | 1 | Vernes 2010 |
| *Wallabia bicolor* | B | Boletales | Boletaceae | *Boletellus* | *Boletellus sp.1 Danks, M* | *Boletellus* | 1 | Danks 2011 |
| *Wallabia bicolor* | B | Boletales | Boletaceae | *Boletellus* | *Boletellus sp.2 Danks, M* | *Boletellus* | 1 | Danks 2011 |
| *Wallabia bicolor* | B | Boletales | Boletaceae | *Boletellus* | *Boletellus sp.3 Danks, M* | *Boletellus* | 1 | Danks 2011 |
| *Wallabia bicolor* | B | Hysterangiales | Mesophelliaceae | *Castoreum* | *Castoreum* | *Castoreum* | 1 | Claridge et al. 2001 |
| *Wallabia bicolor* | B | Boletales | Boletaceae | *Chamonixia* | *Chamonixia* | *Rossbeevera* | 1 | Claridge et al. 2001 |
| *Wallabia bicolor* | B | Boletales | Boletaceae | *Chamonixia* | *Chamonixia* | *Rossbeevera* | 1 | Danks 2011 |
| *Wallabia bicolor* | B | Boletales | Boletaceae | *Chamonixia* | *Chamonixia* | *Rossbeevera* | 1 | Vernes 2010 |
| *Wallabia bicolor* | B | Hysterangiales | Mesophelliaceae | *Chondrogaster* | *Chondrogaster sp.2 O'Malley, A* | *Chondrogaster* | 1 | O’Malley 2012 |
| *Wallabia bicolor* | B | Agaricales | Cortinariaceae | *Cortinarius* | *Cortinarius aff. globuliformis* | *Cortinarius aff. globuliformis* | 1 | Claridge et al. 2001 |
| *Wallabia bicolor* | B | Agaricales | Cortinariaceae | *Cortinarius* | *Cortinarius sp.1 Danks, M* | *Cortinarius* | 1 | Danks 2011 |
| *Wallabia bicolor* | B | Agaricales | Cortinariaceae | *Cortinarius* | *Cortinarius sp.1 O'Malley, A* | *Cortinarius* | 1 | Danks 2011 |
| *Wallabia bicolor* | B | Agaricales | Cortinariaceae | *Cortinarius* | *Cortinarius sp.1 O'Malley, A* | *Cortinarius* | 1 | O’Malley 2012 |
| *Wallabia bicolor* | B | Agaricales | Cortinariaceae | *Cortinarius* | *Cortinarius sp.10 Danks, M* | *Cortinarius* | 1 | Danks 2011 |
| *Wallabia bicolor* | B | Agaricales | Cortinariaceae | *Cortinarius* | *Cortinarius sp.11 Danks, M* | *Cortinarius* | 1 | Danks 2011 |
| *Wallabia bicolor* | B | Agaricales | Cortinariaceae | *Cortinarius* | *Cortinarius sp.13 Danks, M* | *Cortinarius* | 1 | Danks 2011 |
| *Wallabia bicolor* | B | Agaricales | Cortinariaceae | *Cortinarius* | *Cortinarius sp.14 Danks, M* | *Cortinarius* | 1 | Danks 2011 |
| *Wallabia bicolor* | B | Agaricales | Cortinariaceae | *Cortinarius* | *Cortinarius sp.15 Danks, M* | *Cortinarius* | 1 | Danks 2011 |
| *Wallabia bicolor* | B | Agaricales | Cortinariaceae | *Cortinarius* | *Cortinarius sp.2 O'Malley, A* | *Cortinarius* | 1 | O’Malley 2012 |
| *Wallabia bicolor* | B | Agaricales | Cortinariaceae | *Cortinarius* | *Cortinarius sp.3 O'Malley, A* | *Cortinarius* | 1 | O’Malley 2012 |
| *Wallabia bicolor* | B | Agaricales | Cortinariaceae | *Cortinarius* | *Cortinarius sp.4 Danks, M* | *Cortinarius* | 1 | Danks 2011 |
| *Wallabia bicolor* | B | Agaricales | Cortinariaceae | *Cortinarius* | *Cortinarius sp.6 Danks, M* | *Cortinarius* | 1 | Danks 2011 |
| *Wallabia bicolor* | B | Agaricales | Cortinariaceae | *Cortinarius* | *Cortinarius sp.7 Danks, M* | *Cortinarius* | 1 | Danks 2011 |
| *Wallabia bicolor* | B | Agaricales | Cortinariaceae | *Cortinarius* | *Cortinarius sp.8 Danks, M* | *Cortinarius* | 1 | Danks 2011 |
| *Wallabia bicolor* | B | Agaricales | Cortinariaceae | *Cortinarius* | *Cortinarius sp.9 Danks, M* | *Cortinarius* | 1 | Danks 2011 |
| *Wallabia bicolor* | Z | Zygomycetes | unknown | *Densospora* | *Densospora sp.1 O'Malley, A* | *Densospora* | 1 | O’Malley 2012 |
| *Wallabia bicolor* | B | Agaricales | Cortinariaceae | *Descolea* | *Descolea* | *Descolea* | 1 | Danks 2011 |
| *Wallabia bicolor* | B | Agaricales | Cortinariaceae | *Descomyces* | *Descomyces* | *Descomyces* | 1 | Claridge et al. 2001 |
| *Wallabia bicolor* | B | Agaricales | Cortinariaceae | *Descomyces* | *Descomyces aff lebelii* | *Descomyces aff lebelii* | 1 | Danks 2011 |
| *Wallabia bicolor* | A | Elaphomycetales | Elaphomycetaceae | *Elaphomyces* | *Elaphomyces* | *Elaphomyces* | 1 | Vernes 2010 |
| *Wallabia bicolor* | A | Elaphomycetales | Elaphomycetaceae | *Elaphomyces* | *Elaphomyces sp.1 O'Malley, A* | *Elaphomyces* | 1 | O’Malley 2012 |
| *Wallabia bicolor* | A | Elaphomycetales | Elaphomycetaceae | *Elaphomyces* | *Elaphomyces sp.3 O'Malley, A* | *Elaphomyces* | 1 | O’Malley 2012 |
| *Wallabia bicolor* | B | Gomphales | Gomphaceae | *Gautieria* | *Gautieria* | *Gautieria* | 1 | Claridge et al. 2001 |
| *Wallabia bicolor* | B | Gomphales | Gomphaceae | *Gautieria* | *Gautieria* | *Gautieria* | 1 | Vernes 2010 |
| *Wallabia bicolor* | B | Gomphales | Gomphaceae | *Gautieria* | *Gautieria monospora* | *Gautieria monospora* | 1 | O’Malley 2012 |
| *Wallabia bicolor* | G | Glomerales | Glomeraceae | *Glomus* | *Glomus* | *Glomus* | 1 | Danks 2011 |
| *Wallabia bicolor* | B | Russulales | Russulaceae | *Gymnomyces* | *Gymnomyces* | *Gymnomyces* | 1 | Claridge et al. 2001 |
| *Wallabia bicolor* | B | Boletales | Serpulaceae | *Gymnopaxillus* | *Gymnopaxillus* | *Gymnopaxillus* | 1 | Claridge et al. 2001 |
| *Wallabia bicolor* | B | Agaricales | Hydnangiaceae | *Hydnangium* | *Hydnangium carneum* | *Hydnangium carneum* | 1 | Claridge et al. 2001 |
| *Wallabia bicolor* | A | Pezizales | Pezizaceae | *Hydnoplicata* | *Hydnoplicata convoluta* | *Hydnoplicata convoluta* | 1 | Danks 2011 |
| *Wallabia bicolor* | A | Pezizales | Pezizaceae | *Hydnoplicata* | *Hydnoplicata convoluta* | *Hydnoplicata convoluta* | 1 | O’Malley 2012 |
| *Wallabia bicolor* | B | Agaricales | Strophariaceae | *Hymenogaster* | *Hymenogaster* | *Hymenogaster* | 1 | Claridge et al. 2001 |
| *Wallabia bicolor* | B | Hysterangiales | Hysterangiaceae | *Hysterangium* | *Hysterangium* | *Hysterangium* | 1 | Claridge et al. 2001 |
| *Wallabia bicolor* | B | Hysterangiales | Hysterangiaceae | *Hysterangium* | *Hysterangium* | *Hysterangium* | 1 | Vernes 2010 |
| *Wallabia bicolor* | B | Hysterangiales | Hysterangiaceae | *Hysterangium* | *Hysterangium sp.1 Danks, M* | *Hysterangium* | 1 | Danks 2011 |
| *Wallabia bicolor* | B | Hysterangiales | Hysterangiaceae | *Hysterangium* | *Hysterangium sp.2 Danks, M* | *Hysterangium* | 1 | Danks 2011 |
| *Wallabia bicolor* | B | Hysterangiales | Hysterangiaceae | *Hysterangium* | *Hysterangium sp.2 O'Malley, A* | *Hysterangium* | 1 | O’Malley 2012 |
| *Wallabia bicolor* | B | Hysterangiales | Hysterangiaceae | *Hysterangium* | *Hysterangium sp.3 Danks, M* | *Hysterangium* | 1 | Danks 2011 |
| *Wallabia bicolor* | B | Hysterangiales | Hysterangiaceae | *Hysterangium* | *Hysterangium sp.5 O'Malley, A* | *Hysterangium* | 1 | O’Malley 2012 |
| *Wallabia bicolor* | B | Agaricales | Strophariaceae | *Hysterogaster* | *Hysterogaster* | *Hysterogaster* | 1 | Claridge et al. 2001 |
| *Wallabia bicolor* | B | Agaricales | Strophariaceae | *Hysterogaster* | *Hysterogaster sp.1 Vernes, K* | *Hysterogaster* | 1 | Vernes 2010 |
| *Wallabia bicolor* | B | Agaricales | Strophariaceae | *Hysterogaster* | *Hysterogaster sp.2 Vernes, K* | *Hysterogaster* | 1 | Vernes 2010 |
| *Wallabia bicolor* | A | Pezizales | Tuberaceae | *Labyrinthomyces* | *Labyrinthomyces* | *Labyrinthomyces* | 1 | Danks 2011 |
| *Wallabia bicolor* | A | Pezizales | Tuberaceae | *Labyrinthomyces* | *Labyrinthomyces sp.1 Vernes, K* | *Labyrinthomyces* | 1 | Vernes 2010 |
| *Wallabia bicolor* | A | Pezizales | Tuberaceae | *Labyrinthomyces* | *Labyrinthomyces sp.2 Vernes, K* | *Labyrinthomyces* | 1 | O’Malley 2012 |
| *Wallabia bicolor* | A | Pezizales | Tuberaceae | *Labyrinthomyces* | *Labyrinthomyces sp.2 Vernes, K* | *Labyrinthomyces* | 1 | Vernes 2010 |
| *Wallabia bicolor* | A | Pezizales | Tuberaceae | *Labyrinthomyces* | *Labyrinthomyces sp.3 Vernes, K* | *Labyrinthomyces* | 1 | Vernes 2010 |
| *Wallabia bicolor* | B | Russulales | Albatrellaceae | *Leucogaster* | *Leucogaster meridionalis* | *Leucogaster meridionalis* | 1 | O’Malley 2012 |
| *Wallabia bicolor* | B | Hysterangiales | Mesophelliaceae | *Mesophellia* | *Mesophellia* | *Mesophellia* | 1 | Vernes 2010 |
| *Wallabia bicolor* | B | Hysterangiales | Mesophelliaceae | *Mesophellia* | *Mesophellia sp.1 O'Malley, A* | *Mesophellia* | 1 | O’Malley 2012 |
| *Wallabia bicolor* | B | Hysterangiales | Mesophelliaceae | *Mesophellia* | *Mesophellia sp.2 O'Malley, A* | *Mesophellia* | 1 | O’Malley 2012 |
| *Wallabia bicolor* | B | Boletales | Octavianiaceae | *Octaviania* | *Octaviania* | *Octaviania* | 1 | Vernes 2010 |
| *Wallabia bicolor* | B | Boletales | Octavianiaceae | *Octaviania* | *Octaviania sp.1 Danks, M* | *Octaviania* | 1 | Danks 2011 |
| *Wallabia bicolor* | B | Boletales | Octavianiaceae | *Octaviania* | *Octaviania sp.2 O'Malley, A* | *Octaviania* | 1 | O’Malley 2012 |
| *Wallabia bicolor* | B | unknown | unknown | *Pogisperma* | *Pogisperma sp.1 O'Malley, A* | *Pogisperma* | 1 | O’Malley 2012 |
| *Wallabia bicolor* | B | unknown | unknown | *Pogisperma* | *Pogisperma sp.2 O'Malley, A* | *Pogisperma* | 1 | O’Malley 2012 |
| *Wallabia bicolor* | B | Agaricales | Cortinariaceae | *Protoglossum* | *Protoglossum* | *Cortinarius* | 1 | Vernes 2010 |
| *Wallabia bicolor* | B | Agaricales | Cortinariaceae | *Protoglossum* | *Protoglossum sp.1 O'Malley, A* | *Cortinarius* | 1 | O’Malley 2012 |
| *Wallabia bicolor* | B | Agaricales | Cortinariaceae | *Quadrispora* | *Quadrispora musispora* | *Cortinarius musisporus* | 1 | Danks 2011 |
| *Wallabia bicolor* | B | Agaricales | Cortinariaceae | *Quadrispora* | *Quadrispora sp.1 O'Malley, A* | *Cortinarius* | 1 | O’Malley 2012 |
| *Wallabia bicolor* | B | Boletales | Boletaceae | *Rossbeevera* | *Rossbeevera vittatispora* | *Rossbeevera vittatispora* | 1 | O’Malley 2012 |
| *Wallabia bicolor* | B | Russulales | Russulaceae | *Russula* | *Russula aff brunneonigra* | *Russula aff brunneonigra* | 1 | Danks 2011 |
| *Wallabia bicolor* | B | Boletales | Russulaceae | *Russulaceae* | *Russulaceae sp.1 O'Malley, A* |  | 0 | O’Malley 2012 |
| *Wallabia bicolor* | B | Boletales | Russulaceae | *Russulaceae* | *Russulaceae sp.2 O'Malley, A* |  | 0 | O’Malley 2012 |
| *Wallabia bicolor* | B | Boletales | Russulaceae | *Russulaceae* | *Russulaceae sp.3 O'Malley, A* |  | 0 | O’Malley 2012 |
| *Wallabia bicolor* | B | Boletales | Russulaceae | *Russulaceae* | *Russulaceae sp.4 O'Malley, A* |  | 0 | O’Malley 2012 |
| *Wallabia bicolor* | B | Boletales | Sclerodermataceae | *Scleroderma* | *Scleroderma* | *Scleroderma* | 1 | Claridge et al. 2001 |
| *Wallabia bicolor* | B | Boletales | Sclerodermataceae | *Scleroderma* | *Scleroderma aff paradoxum* | *Scleroderma* | 1 | Danks 2011 |
| *Wallabia bicolor* | B | Boletales | Sclerodermataceae | *Scleroderma* | *Scleroderma aff. mcalpinei* | *Scleroderma* | 1 | Claridge et al. 2001 |
| *Wallabia bicolor* | B | Boletales | Sclerodermataceae | *Scleroderma* | *Scleroderma sp.1 Vernes, K* | *Scleroderma* | 1 | Danks 2011 |
| *Wallabia bicolor* | B | Boletales | Sclerodermataceae | *Scleroderma* | *Scleroderma sp.1 Vernes, K* | *Scleroderma* | 1 | Vernes 2010 |
| *Wallabia bicolor* | B | Boletales | Sclerodermataceae | *Scleroderma* | *Scleroderma sp.2 Vernes, K* | *Scleroderma* | 1 | Vernes 2010 |
| *Wallabia bicolor* | B | Boletales | Sclerodermataceae | *Scleroderma* | *Scleroderma sp.3 Vernes, K* | *Scleroderma* | 1 | Danks 2011 |
| *Wallabia bicolor* | B | Boletales | Sclerodermataceae | *Scleroderma* | *Scleroderma sp.3 Vernes, K* | *Scleroderma* | 1 | Vernes 2010 |
| *Wallabia bicolor* | B | Boletales | Sclerodermataceae | *Scleroderma* | *Scleroderma tommayi* | *Scleroderma tommayi* | 1 | Vernes 2010 |
| *Wallabia bicolor* | B | Geastrales | Geastraceae | *Sclerogaster* | *Sclerogaster* | *Sclerogaster* | 1 | Vernes 2010 |
| *Wallabia bicolor* | A | Pezizales | Pyronemataceae | *Sphaerosoma* | *Sphaerosoma* | *Sphaerosoma* | 1 | Vernes 2010 |
| *Wallabia bicolor* | B | Agaricales | Cortinariaceae | *Thaxterogaster* | *Thaxterogaster* | *Cortinarius* | 1 | Vernes 2010 |
| *Wallabia bicolor* | B | Boletales | Boletaceae | *Tylopilus* | *Tylopilus* | *Tylopilus* | 1 | Danks 2011 |
| *Wallabia bicolor* | B | Agaricales | Cortinariaceae | *Unknown* | *Unknown* |  | 0 | Claridge et al. 2001 |
| *Wallabia bicolor* | B | Russulales | Russulaceae | *Unknown* | *Unknown* |  | 0 | Claridge et al. 2001 |
| *Wallabia bicolor* | B | Hysterangiales | Mesophelliaceae | *Unknown* | *Unknown* |  | 0 | Claridge et al. 2001 |
| *Wallabia bicolor* | B | Boletales | Boletaceae | *Unknown* | *Unknown* |  | 0 | Claridge et al. 2001 |
| *Wallabia bicolor* | A | unknown | unknown | *Unknown* | *Unknown* |  | 0 | Danks 2011 |
| *Wallabia bicolor* | B | Agaricales | unknown | *Unknown* | *Unknown* |  | 0 | Danks 2011 |
| *Wallabia bicolor* | B | Hysterangiales | Mesophelliaceae | *Unknown* | *Unknown* |  | 0 | Danks 2011 |
| *Wallabia bicolor* | B | Agaricales | Entolomataceae | *Unknown* | *Unknown* |  | 0 | Danks 2011 |
| *Wallabia bicolor* | B | Boletales | Sclerodermataceae | *Unknown* | *Unknown* |  | 0 | Danks 2011 |
| *Wallabia bicolor* | u | unknown | unknown | *Unknown* | *Unknown* |  | 0 | Danks 2011 |
| *Wallabia bicolor* | B | Hysterangiales | Mesophelliaceae | *Unknown* | *Unknown* |  | 0 | O’Malley 2012 |
| *Wallabia bicolor* | B | Agaricales | Coprinaceae | *Unknown* | *Unknown* |  | 0 | Vernes 2010 |
| *Wallabia bicolor* | B | Hysterangiales | Mesophelliaceae | *Unknown* | *Unknown* |  | 0 | Vernes 2010 |
| *Wallabia bicolor* | B | Agaricales | Strophariaceae | *Unknown* | *Unknown* |  | 0 | Vernes 2010 |
| *Wallabia bicolor* | u | unknown | unknown | *Unknown* | *Unknown [epigeous mushroom] Danks, M* |  | 0 | Danks 2011 |
| *Wallabia bicolor* | u | unknown | unknown | *Unknown* | *Unknown [sequestrate] Danks, M* |  | 0 | Danks 2011 |
| *Wallabia bicolor* | B | Agaricales | Cortinariaceae | *Unknown* | *Unknown sp.1 Claridge, A W* |  | 0 | Claridge et al. 2001 |
| *Wallabia bicolor* | B | Hysterangiales | Mesophelliaceae | *Unknown* | *Unknown sp.1 Claridge, A W* |  | 0 | Claridge et al. 2001 |
| *Wallabia bicolor* | B | Agaricales | Cortinariaceae | *Unknown* | *Unknown sp.1 Danks, M* |  | 0 | Danks 2011 |
| *Wallabia bicolor* | u | unknown | unknown | *Unknown* | *Unknown sp.1 Danks, M* |  | 0 | Danks 2011 |
| *Wallabia bicolor* | B | Boletales | unknown | *Unknown* | *Unknown sp.1 Vernes, K* |  | 0 | Danks 2011 |
| *Wallabia bicolor* | B | Russulales | unknown | *Unknown* | *Unknown sp.1 Vernes, K* |  | 0 | Danks 2011 |
| *Wallabia bicolor* | B | Boletales | unknown | *Unknown* | *Unknown sp.1 Vernes, K* |  | 0 | Vernes 2010 |
| *Wallabia bicolor* | B | Russulales | unknown | *Unknown* | *Unknown sp.1 Vernes, K* |  | 0 | Vernes 2010 |
| *Wallabia bicolor* | u | unknown | unknown | *Unknown* | *Unknown sp.1 Vernes, K* |  | 0 | Vernes 2010 |
| *Wallabia bicolor* | B | Agaricales | Cortinariaceae | *Unknown* | *Unknown sp.2 Claridge, A W* |  | 0 | Claridge et al. 2001 |
| *Wallabia bicolor* | B | Hysterangiales | Mesophelliaceae | *Unknown* | *Unknown sp.2 Claridge, A W* |  | 0 | Claridge et al. 2001 |
| *Wallabia bicolor* | B | unknown | unknown | *Unknown* | *Unknown sp.2 Danks, M* |  | 0 | Danks 2011 |
| *Wallabia bicolor* | B | Agaricales | Cortinariaceae | *Unknown* | *Unknown sp.2 Danks, M* |  | 0 | Danks 2011 |
| *Wallabia bicolor* | B | Boletales | unknown | *Unknown* | *Unknown sp.2 Vernes, K* |  | 0 | Danks 2011 |
| *Wallabia bicolor* | B | Boletales | unknown | *Unknown* | *Unknown sp.2 Vernes, K* |  | 0 | Vernes 2010 |
| *Wallabia bicolor* | B | Russulales | unknown | *Unknown* | *Unknown sp.2 Vernes, K* |  | 0 | Vernes 2010 |
| *Wallabia bicolor* | u | unknown | unknown | *Unknown* | *Unknown sp.2 Vernes, K* |  | 0 | Vernes 2010 |
| *Wallabia bicolor* | B | Agaricales | Agaricaceae | *Unknown* | *Unknown sp.3 Danks, M* |  | 0 | Danks 2011 |
| *Wallabia bicolor* | B | Boletales | unknown | *Unknown* | *Unknown sp.3 Danks, M* |  | 0 | Danks 2011 |
| *Wallabia bicolor* | B | Russulales | unknown | *Unknown* | *Unknown sp.3 Vernes, K* |  | 0 | Danks 2011 |
| *Wallabia bicolor* | B | Russulales | unknown | *Unknown* | *Unknown sp.3 Vernes, K* |  | 0 | Vernes 2010 |
| *Wallabia bicolor* | u | unknown | unknown | *Unknown* | *Unknown sp.3 Vernes, K* |  | 0 | Vernes 2010 |
| *Wallabia bicolor* | B | Agaricales | Agaricaceae | *Unknown* | *Unknown sp.4 Danks, M* |  | 0 | Danks 2011 |
| *Wallabia bicolor* | B | Agaricales | Cortinariaceae | *Unknown* | *Unknown sp.4 Danks, M* |  | 0 | Danks 2011 |
| *Wallabia bicolor* | B | Russulales | unknown | *Unknown* | *Unknown sp.4 Vernes, K* |  | 0 | Danks 2011 |
| *Wallabia bicolor* | B | Russulales | unknown | *Unknown* | *Unknown sp.4 Vernes, K* |  | 0 | Vernes 2010 |
| *Wallabia bicolor* | u | unknown | unknown | *Unknown* | *Unknown sp.4 Vernes, K* |  | 0 | Vernes 2010 |
| *Wallabia bicolor* | B | Agaricales | Agaricaceae | *Unknown* | *Unknown sp.5 Danks, M* |  | 0 | Danks 2011 |
| *Wallabia bicolor* | B | Russulales | unknown | *Unknown* | *Unknown sp.5 Vernes, K* |  | 0 | Danks 2011 |
| *Wallabia bicolor* | B | Agaricales | Agaricaceae | *Unknown* | *Unknown sp.6 Danks, M* |  | 0 | Danks 2011 |
| *Wallabia bicolor* | B | Russulales | unknown | *Unknown* | *Unknown sp.6 Vernes, K* |  | 0 | Danks 2011 |
| *Wallabia bicolor* | B | Russulales | unknown | *Unknown* | *Unknown sp.7 Danks, M* |  | 0 | Danks 2011 |
| *Wallabia bicolor* | B | Russulales | Russulaceae | *Zelleromyces* | *Zelleromyces* | *Zelleromyces* | 1 | Claridge et al. 2001 |
| *Wallabia bicolor* | B | Russulales | Russulaceae | *Zelleromyces* | *Zelleromyces microsporus* | *Zelleromyces microsporus* | 1 | Danks 2011 |
| *Wallabia bicolor* | B | Russulales | Russulaceae | *Zelleromyces* | *Zelleromyces striatus* | *Zelleromyces striatus* | 1 | Danks 2011 |
